# Supplementary material for: Relationships of gut microbiota, short-chain fatty acids, inflammation, and the gut barrier in Parkinson’s disease
Source: Mol Neurodegener. 2021 Feb 8;16:6. doi: 10.1186/s13024-021-00427-6 (PMC7869249; doi:10.1186/s13024-021-00427-6)
Supplement: Supplementary file 1 — Additional file 1: Supplementary Methods. Extended descriptions of measurements for SCFAs and inflammatory and permeability markers and details of statistical analyses. [file 13024_2021_427_MOESM1_ESM.docx]

**Supplementary Methods**

## Short-chain fatty acid measurements

Detection and quantification of SCFAs in the stool samples were performed at the Norwegian University of Life Sciences using a Trace 1310 gas chromatograph (Thermo Fisher Scientific, USA) equipped with an autosampler, a split/splitless injector, a flame ionization detector, and a Stabilwax DA column (30 m, 0.25 mm ID, 0.25 µm film; Thermo Fisher Scientific, USA). Samples were suspended in MilliQ water (1:1 w/v) and subsequently homogenized by vortexing using glass beads (size: 2.5-3.5 mm) in the solution. The samples were then diluted with 0.2% formic acid and 500 µM of an internal standard (2-methylvaleric acid; 1:1 w/v) before a 5 min centrifugation step at 13 000 rpm. The internal standard was included to control equal sampling volume and was used for normalization of the samples. 300 µl of the supernatant was applied to spin columns (0.2 µm filter; VWR, USA) and centrifuged at 10 000 rpm for 5 min. The eluate was transferred to 300 µl GC vials (VWR) and analyzed in the GC-FID instrument. A standard solution was made with final concentrations of 0.1% formic acid and 1000 µM of each of the acids (acetic acid, propionic acid, butyric acid, isobutyric acid, valeric acid, and 2-methylvaleric acid). All acids were purchased from Sigma-Aldrich, Germany. Samples were injected into the GC system in volumes of 0.2 µl. Split mode was set to 2.5:200, and injector and detector temperatures were set to 250 and 275 °C respectively. Helium was used as a carrier gas. Temperature intervals comprised a 2-min step at 90 °C followed by a 6-min increase to 150 °C and a 2-min increase to 245 °C followed by a hold at this temperature for 4.9 min. The software Chromeleon 7.2 (Thermo Fisher Scientific) was used for instrument control, quantification, and data analysis.

## Inflammatory and permeability marker measurements

To prepare stool for the Zonulin Stool ELISA (ALPCO catalog # 30-ZONHU-E01) kit, the dilution/extraction buffer was prepared according to the manufacturer’s instructions and added to thawed raw stool samples at a ratio of 1 mg stool to 50 mL buffer. The assay was then performed according to the manufacturer’s protocol. Reference absorbance values (absorbance at 620 nm) were subtracted from absorbance values at 450 nm, and zonulin concentrations were interpolated from a standard curve using a quadratic regression according to manufacturer’s recommendations and multiplied by the dilution factor (50) to obtain actual concentrations in the stool (ng/mg).

For measurements of stool calprotectin, neutrophil gelatinase-associated lipocalin (NGAL), and cytokines, frozen stool samples were suspended in homogenization buffer (125 mM Tris, 15 mM MgCl2, 2.5 mM EDTA pH 7.2, 1% Triton X-100, 1 tablet protease inhibitors [1697498, Roche] per 10 mL buffer) and homogenized in a TissueLyser-II (QIAGEN, 20 Hz) with a 5 mm metal bead (QIAGEN). Debris were pelleted, and supernatants were collected. The total protein concentration in each supernatant was measured by BCA assay (Pierce) according to the manufacturer’s protocol, and samples were diluted to 6 mg/mL in homogenization buffer.

For the LEGEND MAX™ Human MRP8/14 (Calprotectin) ELISA Kit (Biolegend catalog # 439707), 6 mg/mL stool samples were diluted 1:20 in assay buffer A, and 50 μL (15 μg protein) were loaded into wells. The assay was performed according to the manufacturer’s protocol. Reference absorbance values (absorbance at 570 nm) were subtracted from absorbance values at 450 nm, and calprotectin concentrations were interpolated from a standard curve using a quadratic regression according to manufacturer’s recommendations and multiplied by the dilution factor (20) to obtain actual concentrations in the stool lysate (ng/mL).

Stool NGAL levels were measured using the LEGEND MAX™ Human NGAL (Lipocalin-2) ELISA Kit (Biolegend catalog # 443407). 6 mg/mL stool samples were diluted 1:50 in assay buffer B, and 50 μL (6 μg protein) were loaded into wells. The assay was performed according to the manufacturer’s protocol and analyzed in the same way as calprotectin.

The cytokines interferon gamma (IFNγ), interleukin (IL)-1β, IL-2, IL-4, IL-6, CXCL8, IL-10, IL-12p70, IL-13, and tumor necrosis factor (TNF) were measured in stool and plasma by multiplexed immunoassay using the V-PLEX Proinflammatory Panel 1 Human Kit (Meso Scale Discovery, MSD, Rockville, MD, catalog # K15049D). 6 mg/mL stool lysates were diluted 1:1 in diluent 2, and 50 μL (150 μg) of each sample were loaded. Fifty microliters of undiluted plasma were loaded for each test. The multiplexed immunoassay was performed according to the manufacturer’s protocol using the MSD QuickPlex instrument, and the results were evaluated on the MSD software platform. Resulting values were multiplied by the dilution factor to obtain actual concentrations in the stool lysates and plasma (pg/mL).

Lipopolysaccharide binding protein (LBP) concentrations (μg/mL) were measured in plasma using the Human LBP Kit (MSD catalog # K151IYC) on the MSD platform according to the manufacturer’s protocol.

All samples were run in duplicate in each assay, and roughly equal numbers of control and PD subject samples were included on each plate. In the multiplexed immunoassays, stool and plasma samples from each subject were run on the same plate. No systematic differences were observed in values obtained from different runs.

## Statistical analyses

We performed all statistical analyses in R,^32^ and managed microbiota data with the phyloseq package.^33^ The full analysis workflow is available as a supplementary file (Supplementary R Markdown). For statistical comparisons between SCFAs/markers, clinical variables, enterotypes, and alpha diversity, we used the Kruskal-Wallis test, Wilcoxon rank sum test, or Pearson correlations depending on the types of variables. When multiple comparison corrections were included, we used the Benjamini & Hochberg false discovery rate, correcting SCFAs, stool markers, and plasma markers separately. Principal Component Analysis (PCA) was used to merge intercorrelated inflammatory markers.

In all microbiome-related comparisons, we tested the full data (patients and control subjects together), and PD-only and control-only subsets. Additionally, we ran confounder-corrected comparisons specific to each test. For alpha and beta diversity comparisons, we used OTU-level data subsampled to the lowest number of reads in a sample (7165). Alpha diversity comparisons included two measures: Shannon and inverse Simpson (both indices include richness and evenness); after preliminary screening with correlations, we explored the most interesting cases with linear regression - corrected for PD/control and sex - with the SCFA or marker variables log-transformed to address heteroskedasticity. Beta diversity was evaluated with PERMANOVAs on Bray-Curtis distance matrices, run with the adonis (univariable) and adonis2 (multivariable) tests from the vegan package^34^, and visualized with Non-Metric Multidimensional scaling (NMDS). The variables in confounder-corrected adonis2 models were sex, Rome III 9-15 sum score, and BMI. For differential abundance testing, we used DESeq2^35^ on trimmed data (genera and families with > 5 reads in > 50% of the samples), and ran four models: full data, PD-only, control-only, and full data corrected for PD/control status, Rome III 9-15 sum score, and sex.
