# Supplementary material for: Relationships of gut microbiota, short-chain fatty acids, inflammation, and the gut barrier in Parkinson’s disease
Source: Mol Neurodegener. 2021 Feb 8;16:6. doi: 10.1186/s13024-021-00427-6 (PMC7869249; doi:10.1186/s13024-021-00427-6)
Supplement: Supplementary file 2 — Additional file 2: R Markdown. The full R code used for the analyses with output. [file 13024_2021_427_MOESM2_ESM.pdf]

R analyses on Parkinson's disease, gut microbiota, inflammatory markers and short-chain fatty acids

Velma T. E. Aho

08 December 2020

Contents

1 Setup **2**
- 1.1 Data import . . . 2
- 1.2 Outlier check . . . 4
- 1.3 Subjects and demographics . . . 7

2 Comparisons without microbiota data **15**
- 2.1 Intercorrelations of inflammatory markers and SCFAs . . . 15
- 2.2 PCA with inflammatory markers . . . 18
  - 2.2.1 Stool . . . 18
  - 2.2.2 Plasma . . . 21
- 2.3 Correlations between clinical variables . . . 23
  - 2.3.1 SCFAs, markers vs other variables . . . 24

3 Comparisons with microbiota data **38**
- 3.1 Alpha diversity . . . 40
  - 3.1.1 Full data (PD + C together) . . . 40
  - 3.1.2 Control only / PD only . . . 41
  - 3.1.3 Corrected for PD vs C . . . 43
  - 3.1.4 Analyses with additional confounders . . . 46
- 3.2 Beta diversity . . . 53
  - 3.2.1 Full data (PD + C together) . . . 53
  - 3.2.2 Corrected for PD vs control . . . 54
  - 3.2.3 Control only / PD only . . . 55
  - 3.2.4 Analyses with additional confounders . . . 56
  - 3.2.5 Interaction effects . . . 59
- 3.3 Revision: Additional comparisons of PD-related variables and diversity . . . 61
- 3.4 Enterotypes . . . 66
- 3.5 Differential abundance with DESeq2 . . . 70
  - 3.5.1 SCFAs . . . 71
  - 3.5.2 Inflammatory markers . . . 77

4 Revision: Summary table of key results / Additional file 13 **83**

5 Additional analyses **87**
- 5.1 Sample storage time . . . 87

6 Session info **89**

1 Setup

```
# Load required packages
options(kableExtra.latex.load_packages=FALSE)
library("kableExtra")
library("ggplot2")
library("gridExtra")
library("reshape2")
library("Hmisc")
library("factoextra")
library("gtools")
library("ggcorrplot")
library("GGally")
library("phyloseq")
library("vegan")
library("DESeq2")
library("mgsub")

# Dimensions for figures:
maxwd <- 170
maxhi <- 225
```

1.1 Data import

Import inflammatory marker data, mark as NA the values that are over the measurement range for calprotectin and NGAL, and trim subjects that only have values for LBP:

```
inf_df <- read.csv("Inputs/cytokine_data.csv", row.names = 1)

# Save the variable names for further use:
inf_vars <- colnames(inf_df)
inf_vars_s <- grep("^S", inf_vars, value = TRUE) # Stool variables
inf_vars_p <- grep("^p", inf_vars, value = TRUE) # Plasma variables

# Change the "TOO HIGH" values in stool NGAL to NA
inf_df$SNGAL <- as.character(inf_df$SNGAL)
inf_df$SNGAL[inf_df$SNGAL == "TOO HIGH"] <- NA
inf_df$SNGAL <- as.numeric(inf_df$SNGAL)

# Change the "TOO HIGH" values in stool calprotectin to NA
inf_df$Scalprotectin <- as.character(inf_df$Scalprotectin)
inf_df$Scalprotectin[inf_df$Scalprotectin == "TOO HIGH"] <- NA
inf_df$Scalprotectin <- as.numeric(inf_df$Scalprotectin)

# Samples with more than one missing value?
print(inf_df[names(which(rowSums(is.na(inf_df)) > 1)), ])
```

```
##            pLBP SNGAL Scalprotectin Szonulin SIFNg SIL10 SIL12p70 SIL13 SIL1b
## C0052 4.653704    NA            NA       NA    NA    NA       NA    NA    NA
## C0124 4.283837    NA            NA       NA    NA    NA       NA    NA    NA
##       SIL2 SIL4 SIL6 SCXCL8 STNF pIFNg pIL10 pIL12p70 pIL13 pIL1b pIL2 pIL4
## C0052   NA   NA   NA     NA   NA    NA    NA       NA    NA    NA   NA   NA
## C0124   NA   NA   NA     NA   NA    NA    NA       NA    NA    NA   NA   NA
##       pIL6 pCXCL8 pTNF
```

```
## C0052   NA      NA   NA
## C0124   NA      NA   NA
```

```
# Two samples have NA for everything except pLBP. Drop these two:
inf_df <- inf_df[-(which(rowSums(is.na(inf_df)) > 1)), ]
```

Import clinical metadata and SCFA measurements, trim to subjects fit to use based on the follow-up analyses (Aho et al. 2019):

```
clin_df <- read.csv("Inputs/pdcy_meta_v5.csv", row.names = 1,
                    stringsAsFactors = TRUE)

# Subset to follow-up timepoint samples deemed "OK to use"
clin_df <- subset(clin_df, Timepoint == "followup" & OK_to_use == "yes")

# Remove timepoint identifier from row names
rownames(clin_df) <- sub("N", "", rownames(clin_df))

# Make sure factor variables are in correct form
for(i in colnames(clin_df)){
  if(identical(names(table(clin_df[, i])), c("0", "1")) | is.factor(clin_df[, i])){
    clin_df[, i] <- factor(clin_df[, i])}}

# Two additional variables need to be fixed manually:
clin_df$tobacco_when_last <- factor(clin_df$tobacco_when_last)
clin_df$meds_rasagiline_mg <- as.numeric(as.character(clin_df$meds_rasagiline_mg))

# Remove some redundant variables
clin_df$OK_to_use <- NULL
clin_df$Timepoint <- NULL

# Remove variables with a lot of missing values
clin_df <- clin_df[, -which(colSums(is.na(clin_df)) > 100)]

# Remove variables with a lot of zeros
clin_df <- clin_df[, -which(colSums(clin_df == 0, na.rm = TRUE) > 120)]
```

Import enterotype variable and add to clinical data:

```
pd_et <- read.csv("Inputs/enterotyping_results.txt", row.names = 1)

# Trim to follow-up only
pd_et <- pd_et[grep("N", rownames(pd_et)), ]
rownames(pd_et) <- sub("N", "", rownames(pd_et))

# Add to main metadata df
clin_df$Enterotype <- pd_et[rownames(clin_df), "ET"]
```

Trim subjects with missing values for any of the big three SCFAs:

```
# Make a list of the SCFA variables
scfa_vars <- grep("_acid", colnames(clin_df), value = TRUE)
# Reorder for convenience
scfa_vars <- scfa_vars[c(1, 2, 4, 3, 5:6)]

# Exclude subjects with missing values for any of the big 3 SCFAs
clin_df <- subset(clin_df, !rowSums(is.na(clin_df[, scfa_vars[1:3]])) > 0)
```

Add a "total SCFAs" sum variable:

```
clin_df$total_SCFAs <- rowSums(clin_df[, scfa_vars], na.rm = TRUE)
scfa_vars <- c("total_SCFAs", scfa_vars)
```

Combine the immune marker data and the rest of the metadata:

```
# Match the immune marker data to the trimmed clinical data
inf_df <- inf_df[rownames(clin_df), ]

# Combine the two dfs
pdcy_meta <- cbind(clin_df, inf_df)
```

1.2 Outlier check

Plot values of the variables of interest in all samples to see how they are distributed.

SCFAs

Plot all SCFA measurement values (dashed line is the 3rd quartile + 1.5*IQR):

```
# Plotting function
ol_plot <- function(df, var){
  ggplot(df, aes_string(x = "Subject", y = var)) +
      geom_point() +
      theme_bw(base_size = 8) +
      ylab(NULL) +
      ggtitle(var) +
    geom_hline(yintercept = (quantile(df[,var], 0.75, na.rm = TRUE) +
                                1.5*IQR(df[,var], na.rm = TRUE)),
               linetype = 2, color = "red") +
      theme(panel.grid = element_blank(),
            axis.text.x = element_blank(),
            axis.ticks.x = element_blank(),
            plot.title = element_text(size = 9))
}

scfa_ol <- lapply(scfa_vars, function(x) ol_plot(pdcy_meta, x))
do.call("grid.arrange", c(scfa_ol, ncol = 4))
```

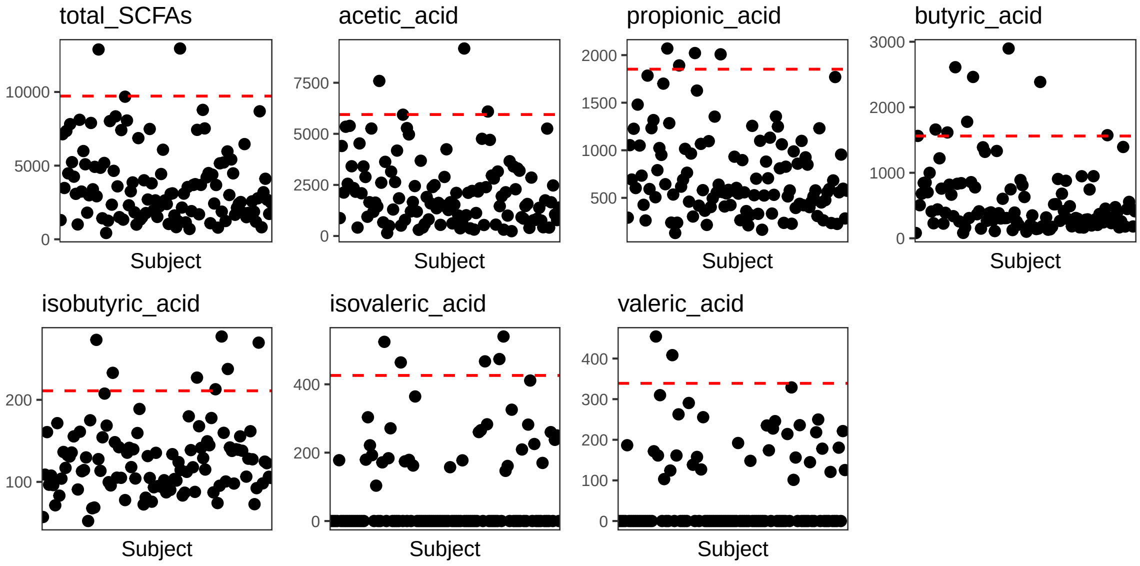

The SCFA data seemed quite evenly distributed. Since most subjects' value for valeric and isovaleric acid was zero, convert these two into binary variables (0 vs > 0):

```
# Make new binary variables for the two SCFAs
pdcy_meta$isovaleric_acid_binary <- as.numeric(pdcy_meta$isovaleric_acid > 0)
pdcy_meta$valeric_acid_binary <- as.numeric(pdcy_meta$isovaleric_acid > 0)

# Replace the numeric variables in the list of SCFA vars with the binary ones
scfa_vars[grep("valeric", scfa_vars)] <- paste(scfa_vars[grep("valeric", scfa_vars)],
                                               "binary", sep = "_")
```

Inflammatory markers

Plot all inflammatory marker values:

```
inf_ol <- lapply(c(inf_vars_s, inf_vars_p), function(x) ol_plot(pdcy_meta, x))
do.call("grid.arrange", c(inf_ol[1:13], ncol = 4))
```

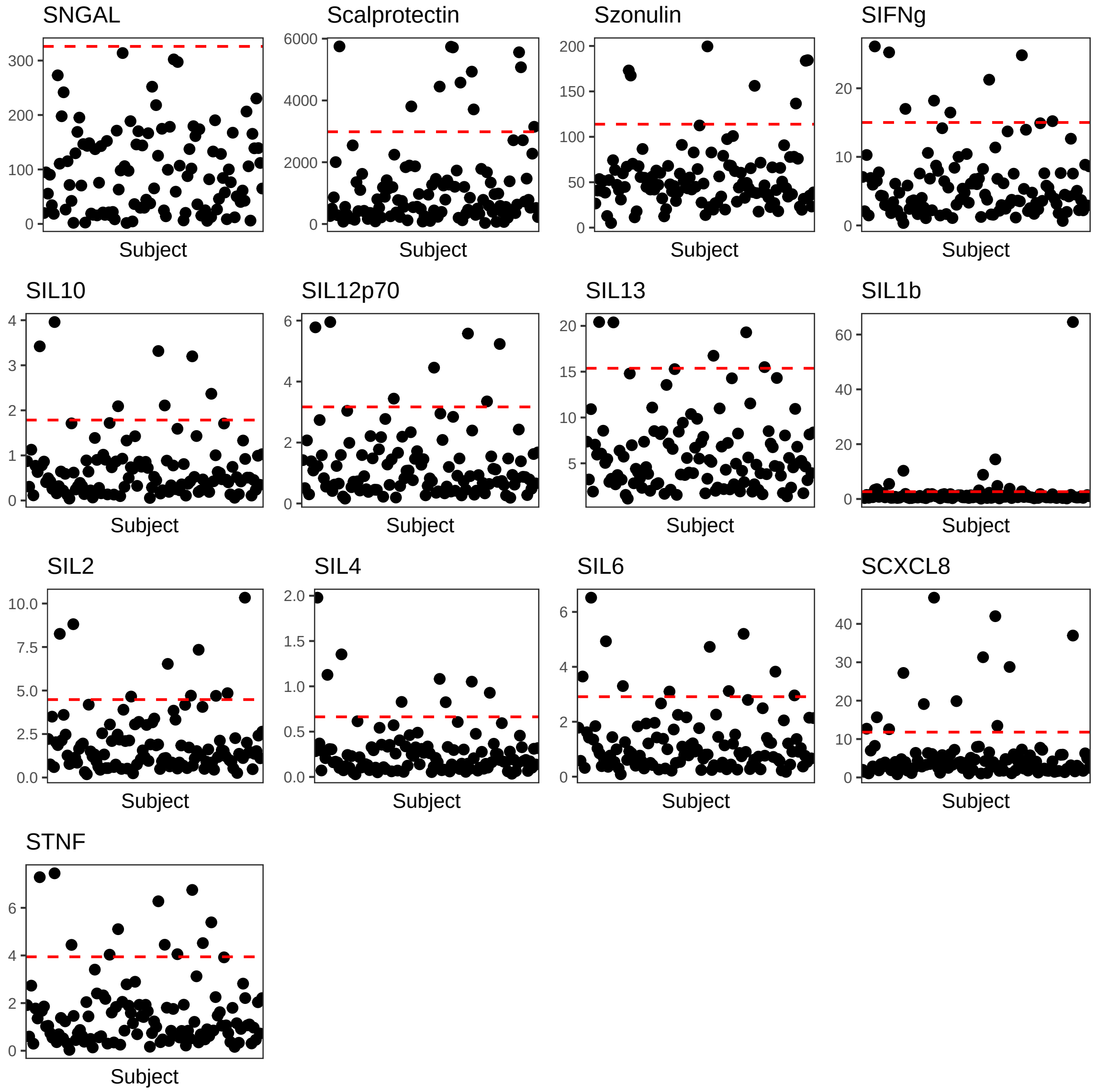


```
do.call("grid.arrange", c(inf_ol[14:24], ncol = 4))
```

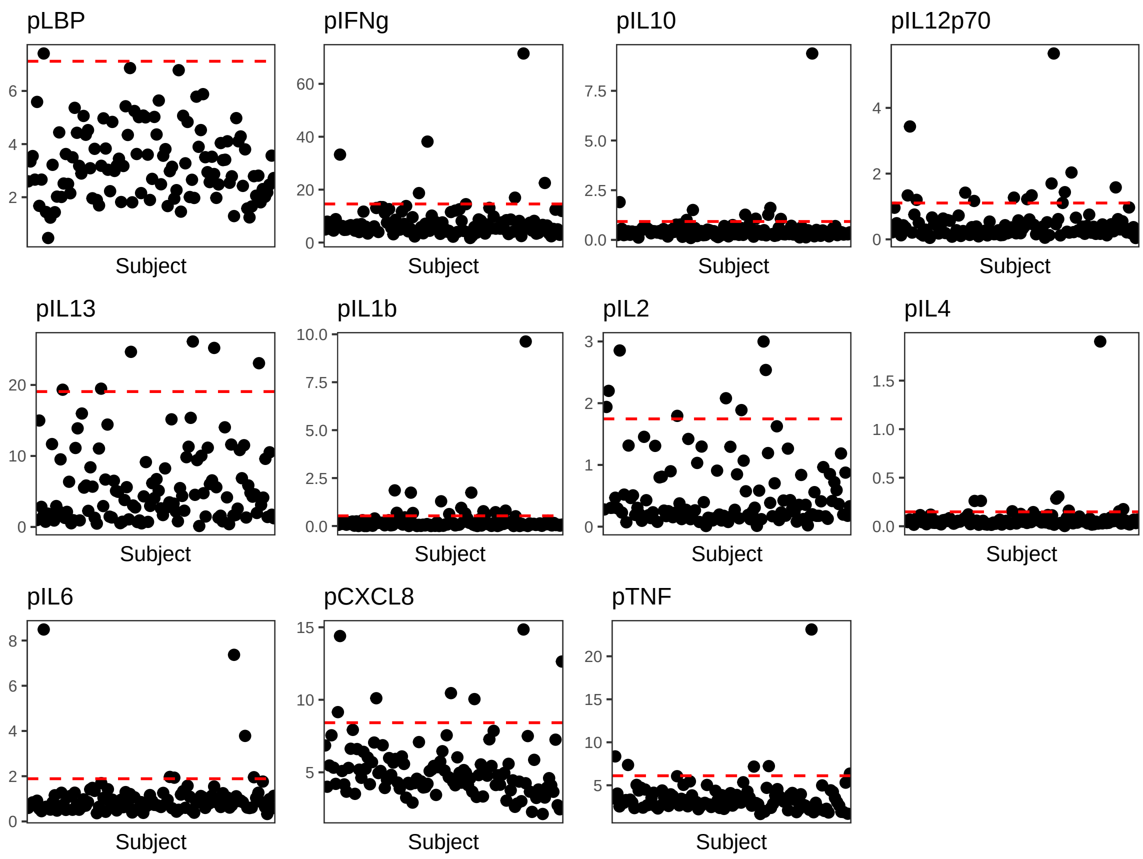

There was a single, extreme outlier point in SIL1b, pIL10, pIL1b, pIL4 and pTNF. The subjects of these points were:

```
sapply(c("SIL1b", "pIL10", "pIL1b", "pIL4", "pTNF"), function(x)
  as.vector(pdcy_meta[which(pdcy_meta[, x] == max(pdcy_meta[, x])), "Subject"]))
```

```
##   SIL1b   pIL10   pIL1b    pIL4    pTNF
## "P0099" "P0071" "P0071" "P0071" "P0071"
```

For plasma measurements, the outlier was the same subject. To make sure this subject would not cause problems, exclude them from the data. Replace the one outlier SIL1b value with NA.

```
pdcy_meta[pdcy_meta$Subject == "P0099", "SIL1b"] <- NA
pdcy_meta <- subset(pdcy_meta, Subject != "P0071")
```

1.3 Subjects and demographics

After the exclusions, the number of samples remaining for the analyses was as follows:

```
kable(table(pdcy_meta$Group), col.names = c("Group", "n"), booktabs = TRUE)
```

Group	n
C	56
P	55

```
# Export the final metadata for possible future use
write.csv(pdcy_meta, "Outputs/pdcy_meta_from_R.csv")
```

Table 1

Make tables of basic demographics and measures for the samples:

Categorical confounders

```
# List all binary variables
clin_table_cat_vars <- names(which(lengths(sapply(pdcy_meta, levels)) == 2))
# Exclude PD vs C
clin_table_cat_vars <- grep("Group", clin_table_cat_vars, invert = TRUE, value = TRUE)

# Summarize
clin_table_cat <- sapply(clin_table_cat_vars,
              function(x){
                out <- c(paste(levels(pdcy_meta[, x])[2]),
                         prop.table(
                           table(pdcy_meta[,c("Group", x)]), 1)[1,2]*100,
                         prop.table(
                           table(pdcy_meta[,c("Group", x)]), 1)[2,2]*100,
                         fisher.test(
                           table(pdcy_meta[,c("Group", x)]))$p.value)
                names(out) <- c("var", "C", "P", "pVal")
                return(out)})

# Reorganize
clin_table_cat <- as.data.frame(t(clin_table_cat))
clin_table_cat$var <- capitalize(
  gsub("_", " ", paste0(rownames(clin_table_cat), " (% ",
                        sub(1, "yes", clin_table_cat$var), ")")))
clin_table_cat[, 2:4] <- sapply(clin_table_cat[, 2:4], function(x)
  as.numeric(as.character(x)))

# Trim to significant variables + sex
clin_table_cat_sig <- rbind(clin_table_cat["sex",],
                            subset(clin_table_cat, pVal < 0.05))

# Show
kable_styling(kable(clin_table_cat_sig, digits = 4,
                    booktabs = TRUE, row.names = FALSE,
                    linesep = ""), font_size = 8)
```

var	C	P	pVal
Sex (% M)	48.2143	52.7273	0.7056
History TIA ischemic stroke (% yes)	37.5000	5.5556	0.0000
Meds ca antagonist (% yes)	19.6429	5.4545	0.0424
Meds COMT inhibitor (% yes)	0.0000	27.2727	0.0000
Meds dopa (% yes)	0.0000	76.3636	0.0000
Meds dopamine agonist (% yes)	0.0000	80.0000	0.0000
Meds MAO inhibitor (% yes)	0.0000	70.9091	0.0000
Meds statin (% yes)	48.2143	18.1818	0.0012
Rome III IBS criteria fulfilled (% yes)	7.1429	36.3636	0.0002
Rome III IBS possible (% yes)	7.1429	38.1818	0.0001

```
# Export full results
write.csv(clin_table_cat, "Outputs/table1cat.csv", row.names = FALSE)
```

Numeric confounders

```r
# Function for summarizing
meanSdP <- function(df, x){
  out <- c(
    paste0(
      round(mean(subset(df, Group == "C")[, x], na.rm = TRUE), digits = 2), " ± ",
      round(sd(subset(df, Group == "C")[, x], na.rm = TRUE), digits = 2)),
    paste0(
      round(mean(subset(df, Group == "P")[, x], na.rm = TRUE), digits = 2), " ± ",
      round(sd(subset(df, Group == "P")[, x], na.rm = TRUE), digits = 2)),
    round(wilcox.test(as.formula(paste(x, "~ Group")), df)$p.value, digits = 4))
  names(out) <- c("C", "P", "pVal")
  return(out)
}

# Grab all numeric variables
clin_table_num_vars <- colnames(pdcy_meta)[sapply(
  pdcy_meta, class) %in% c("numeric", "integer")]
# Exclude variables of interest
clin_table_num_vars <- clin_table_num_vars[1:(grep("acid", clin_table_num_vars)[1]-1)]
# Drop some PD-specific variables
clin_table_num_vars <- clin_table_num_vars[!(clin_table_num_vars %in% c(
  grep("onset", clin_table_num_vars, value = TRUE),
  grep("jankovic", clin_table_num_vars, value = TRUE),
  grep("poletti", clin_table_num_vars, value = TRUE),
  grep("UPDRS", clin_table_num_vars, value = TRUE)))]

# Summarize & rearrange
clin_table_num <- sapply(clin_table_num_vars, function(x) meanSdP(pdcy_meta, x))
clin_table_num <- as.data.frame(t(clin_table_num))
clin_table_num$pVal <- as.numeric(as.character(clin_table_num$pVal))
clin_table_num <- data.frame(var = capitalize(
  gsub("_", " ", rownames(clin_table_num))), clin_table_num)

# Show
kable_styling(kable(clin_table_num, booktabs = TRUE, row.names = FALSE,
                    linesep = ""), font_size = 8)
```

var	C	P	pVal
Age at stool collection	66.38 ± 6.73	67.63 ± 5.21	0.2930
BMI	26.7 ± 3.56	27.63 ± 4.74	0.2612
GDS15	0.84 ± 1.39	4.36 ± 3.72	0.0000
LED	0 ± 0	608.61 ± 301.46	0.0000
Meds entacapone mg	0 ± 0	238.18 ± 413.88	0.0000
Meds levodopa entacapone mg	0 ± 0	130.45 ± 243.94	0.0000
Meds levodopa IR mg	0 ± 0	152.73 ± 209.81	0.0000
Meds pramipexole mg	0 ± 0	0.6 ± 0.9	0.0000
Meds rasagiline mg	0 ± 0	0.36 ± 0.49	0.0000
Meds ropinirole mg	0 ± 0	3.05 ± 5.76	0.0000
Meds selegeline mg	0 ± 0	3.19 ± 4.64	0.0000
MMSE total	28.82 ± 1.49	28.15 ± 1.7	0.0160
NMSQuest total	2.45 ± 2.1	10.91 ± 5.14	0.0000
NMSS total	7.09 ± 6.35	48.36 ± 36.55	0.0000
RBDSQ	1.73 ± 1.29	5.07 ± 3.07	0.0000
RLS	0.16 ± 0.63	1.64 ± 1.77	0.0000
Rome III constip defec sumscore 9.15	2.46 ± 3.16	7.45 ± 5.22	0.0000
SCS PD total	0.18 ± 0.47	4.38 ± 4.24	0.0000
SDQ total	0.5 ± 0.89	5.73 ± 4.82	0.0000
Sniffinsticks	13.52 ± 1.55	7.35 ± 2.95	0.0000
Wexner total	2.2 ± 2.14	6.38 ± 4.69	0.0000

```
# Export full results
write.csv(clin_table_num, "Outputs/table1num.csv", row.names = FALSE)
```

Additional file 3

Descriptive values and p-values for PD/control and the variables of interest (SCFAs + inflammatory markers):

```
# List variables of interest (with isovaleric and valeric acid as numeric)
basic_pdc_vars <- c(scfa_vars[1:5], "isovaleric_acid", "valeric_acid",
                    inf_vars_s, inf_vars_p)

# Summarize
table_basic_pdc <- sapply(basic_pdc_vars, function(x) meanSdP(pdcy_meta, x))

# Function for fixing variable names
varLabelFix <- function(x){
  capitalize(
    gsub("_", " ", sub("^p([A-Z]{1})", "Plasma \\1", sub("^S", "Stool ", x))))
}

# Reorganize and make nicer row labels
table_basic_pdc <- as.data.frame(t(table_basic_pdc))
table_basic_pdc <- data.frame(var = varLabelFix(rownames(table_basic_pdc)),
                              table_basic_pdc)

# Show
kable_styling(kable(table_basic_pdc, booktabs = TRUE, row.names = FALSE,
                    linesep = ""), font_size = 8)
```

var	C	P	pVal
Total SCFAs	4209.14 ± 2688.93	3288.72 ± 2388.22	0.0506
Acetic acid	2390.65 ± 1699.99	1938.61 ± 1669.64	0.0922
Propionic acid	840.13 ± 509.61	644.38 ± 351.39	0.0422
Butyric acid	736.26 ± 633.78	424.61 ± 405.92	0.0013
Isobutyric acid	123.99 ± 43.24	131.02 ± 46.37	0.6149
Isovaleric acid	65.59 ± 125.07	92.63 ± 149.41	0.4013
Valeric acid	60.24 ± 113.12	65.82 ± 99.15	0.5337
Stool NGAL	93.88 ± 78.46	100.92 ± 74.89	0.5313
Stool calprotectin	872.01 ± 1000.65	1499.67 ± 1616.72	0.0233
Stool zonulin	52.86 ± 30.38	58.53 ± 42.73	0.8066
Stool IFNg	6.35 ± 5.5	5.68 ± 4.8	0.5416
Stool IL10	0.73 ± 0.73	0.67 ± 0.69	0.4846
Stool IL12p70	1.36 ± 1.17	1.13 ± 1.06	0.1778
Stool IL13	6.26 ± 4.32	5.73 ± 3.99	0.4773
Stool IL1b	1.24 ± 1.53	1.38 ± 2.28	0.505
Stool IL2	1.98 ± 1.69	1.9 ± 1.88	0.4809
Stool IL4	0.31 ± 0.34	0.25 ± 0.24	0.2818
Stool IL6	1.29 ± 1.18	1.15 ± 1.05	0.3779
Stool CXCL8	5.82 ± 7.53	5.96 ± 8.6	0.5299
Stool TNF	1.63 ± 1.56	1.54 ± 1.51	0.6434
Plasma LBP	3.38 ± 1.45	3.21 ± 1.26	0.5455
Plasma IFNg	8.04 ± 6.33	6.77 ± 4.04	0.2513
Plasma IL10	0.45 ± 0.31	0.45 ± 0.3	0.7975
Plasma IL12p70	0.46 ± 0.53	0.58 ± 0.82	0.2637
Plasma IL13	5.35 ± 5.64	6.48 ± 5.99	0.1188
Plasma IL1b	0.23 ± 0.38	0.21 ± 0.31	0.906
Plasma IL2	0.56 ± 0.64	0.56 ± 0.61	0.6518
Plasma IL4	0.06 ± 0.05	0.07 ± 0.06	0.3747
Plasma IL6	0.96 ± 1.07	0.99 ± 0.55	0.1816
Plasma CXCL8	5.46 ± 1.87	4.81 ± 2.03	0.008
Plasma TNF	3.53 ± 1.23	3.25 ± 1.31	0.1119

Additional file 3: Table split by sex

Check for differences between PD and control when data is split to male or female.

```r
# Summarize F-only and M-only subsets
table_basic_pdc_F <- sapply(basic_pdc_vars, function(x)
  meanSdP(subset(pdcy_meta, sex == "F"), x))
table_basic_pdc_M <- sapply(basic_pdc_vars, function(x)
  meanSdP(subset(pdcy_meta, sex == "M"), x))

# Reorganize and make nicer row labels
table_basic_pdc_F <- as.data.frame(t(table_basic_pdc_F))
colnames(table_basic_pdc_F) <- paste(colnames(table_basic_pdc_F),
                                     "female", sep = "_")
table_basic_pdc_F$pVal_female <- as.numeric(
  as.character(table_basic_pdc_F$pVal_female))

table_basic_pdc_M <- as.data.frame(t(table_basic_pdc_M))
colnames(table_basic_pdc_M) <- paste(colnames(table_basic_pdc_M),
                                     "male", sep = "_")
table_basic_pdc_M$pVal_male <- as.numeric(
  as.character(table_basic_pdc_M$pVal_male))

# Combine dfs
table_basic_pdc_sex <- cbind(table_basic_pdc_F, table_basic_pdc_M)
```

```r
# Fix rownames
table_basic_pdc_sex <- data.frame(var = varLabelFix(rownames(table_basic_pdc_sex)),
                                  table_basic_pdc_sex)

# Significant results?
kable_styling(kable(subset(table_basic_pdc_sex, pVal_female < 0.1 | pVal_male < 0.1),
      digits = 3, booktabs = TRUE, row.names = FALSE), font_size = 7)
```

var	C_female	P_female	pVal_female	C_male	P_male	pVal_male
Total SCFAs	3670.79 ± 2214.48	3301.34 ± 2513.4	0.335	4787.37 ± 3056.69	3277.41 ± 2314.98	0.052
Acetic acid	2012.88 ± 1374.1	1965.12 ± 1849.39	0.487	2796.4 ± 1936.46	1914.84 ± 1523.55	0.064
Propionic acid	781.83 ± 480.18	640.39 ± 283.66	0.388	902.75 ± 541.49	647.95 ± 407.75	0.057
Butyric acid	658.33 ± 661.11	436.15 ± 461.58	0.100	819.95 ± 604.11	414.26 ± 356.8	0.003
Stool calprotectin	665.37 ± 772.91	1502.36 ± 1696.54	0.019	1102.49 ± 1178.53	1497.26 ± 1571.93	0.489
Stool IFNg	6.41 ± 4.01	4.85 ± 3.83	0.078	6.27 ± 6.83	6.42 ± 5.49	0.282
Stool IL10	0.76 ± 0.49	0.6 ± 0.61	0.090	0.71 ± 0.94	0.73 ± 0.77	0.353
Stool IL12p70	1.44 ± 0.86	1.02 ± 0.89	0.040	1.27 ± 1.45	1.22 ± 1.19	0.558
Stool IL13	6.73 ± 3.63	5.15 ± 3.86	0.065	5.76 ± 4.98	6.24 ± 4.11	0.233
Stool IL2	2.09 ± 1.23	1.52 ± 1.24	0.070	1.85 ± 2.09	2.24 ± 2.27	0.312
Stool IL4	0.29 ± 0.18	0.23 ± 0.23	0.078	0.33 ± 0.45	0.26 ± 0.25	0.591
Stool IL6	1.35 ± 0.9	0.94 ± 0.8	0.047	1.23 ± 1.44	1.34 ± 1.22	0.304
Plasma IL13	7.03 ± 6.64	6.53 ± 6.78	0.795	3.55 ± 3.65	6.44 ± 5.3	0.005
Plasma IL4	0.08 ± 0.06	0.07 ± 0.06	0.498	0.04 ± 0.03	0.07 ± 0.06	0.057
Plasma CXCL8	5.25 ± 1.37	4.67 ± 1.94	0.017	5.68 ± 2.29	4.95 ± 2.13	0.145

```r
# Combine with the results for full data
table_basic_pdc_final <- cbind(table_basic_pdc, table_basic_pdc_sex[, 2:7])
```

Revision: P-values for contrasting sex within PD or C subgroups

As suggested by a reviewer, instead of contrasting each variable between PD/C within males or females only, contrast each variable between F/M in PD-only or C-only subgroups.

```r
# P-values for PD-only and C-only subsets
p_basic_sex <- data.frame(
  pVal_FvsM_PD = sapply(basic_pdc_vars, function(x)
  round(wilcox.test(as.formula(paste(x, "~ sex")),
                    subset(pdcy_meta, Group == "P"))$p.value,
        digits = 4)),
  pVal_FvsM_C = sapply(basic_pdc_vars, function(x)
  round(wilcox.test(as.formula(paste(x, "~ sex")),
                    subset(pdcy_meta, Group == "C"))$p.value,
        digits = 4)))

# Significant results?
kable_styling(kable(subset(p_basic_sex, pVal_FvsM_PD < 0.1 | pVal_FvsM_C < 0.1),
      digits = 3, booktabs = TRUE, row.names = FALSE), font_size = 7)
```

pVal_FvsM_PD	pVal_FvsM_C
0.967	0.051
0.783	0.071
0.287	0.066
0.335	0.041
0.141	0.059
0.056	0.935
0.112	0.071
0.417	0.066
0.031	0.105
0.900	0.071
0.530	0.064
0.538	0.014
0.927	0.010
0.335	0.038

```
# Combine with the results for full data
table_basic_pdc_final <- cbind(table_basic_pdc_final, p_basic_sex)

# Export
write.csv(table_basic_pdc_final, "Outputs/add3_scfa_inf_table.csv", row.names = FALSE)
```

Fig 1

Boxplots for the variables that differ significantly in the full data and one of the subsets:

```
# Data frame for figure
basic_pdc_fig_vars <- row.names(subset(table_basic_pdc_final,
                              as.numeric(as.character(pVal)) < 0.05 &
                      (pVal_female < 0.05 | pVal_male < 0.05)))
basic_pdc_fig_df <- melt(pdcy_meta[, c("sex", "Group", basic_pdc_fig_vars)])

# Better scales
basic_pdc_fig_df[basic_pdc_fig_df$variable == "butyric_acid", "value"] <-
  basic_pdc_fig_df[basic_pdc_fig_df$variable == "butyric_acid", "value"]/1000
basic_pdc_fig_df[basic_pdc_fig_df$variable == "Scalprotectin", "value"] <-
  basic_pdc_fig_df[basic_pdc_fig_df$variable == "Scalprotectin", "value"]/1000

# Data frame for p-value annotations
pdc_sig_pstars <- subset(data.frame(Var = rownames(table_basic_pdc_final),
                                    pVal_F = table_basic_pdc_final$pVal_female,
                                    pVal_M = table_basic_pdc_final$pVal_male),
                         Var %in% basic_pdc_fig_vars)
pdc_sig_pstars <- subset(melt(pdc_sig_pstars), value < 0.05)
pdc_sig_pstars$sex <- sub(".*_", "", pdc_sig_pstars$variable)
pdc_sig_pstars$stars <- stars.pval(pdc_sig_pstars$value)
pdc_sig_pstars$y <- c(6.2, 13.5, 2.8)
pdc_sig_pstars$ytext <- c(6.3, 13.8, 2.9)
colnames(pdc_sig_pstars)[1:2] <- c("variable", "pVariable")
pdc_sig_pstars$variable <- factor(pdc_sig_pstars$variable,
                                  levels = c("butyric_acid",
                                             "Scalprotectin",
                                             "pCXCL8"))

# Control/PD labels
pdc_labels <- setNames(c("control", "Parkinson"), c("C", "P"))
```

```r
# Plot
pdc_sig_plots <- ggplot(basic_pdc_fig_df, aes(x = Group, y = value)) +
  geom_boxplot(width = 0.7, outlier.size = 0.5) +
  theme_bw(base_size = 12) +
  scale_x_discrete(labels = pdc_labels) +
  scale_y_continuous(expand = expansion(mult = 0.1)) +
  facet_grid(variable ~ sex, scales = "free", labeller = labeller(
    variable = setNames(c("Butyric acid\n(mmol/l)",
                          "Stool calprotectin\n(ng/mg total protein)",
                          "Plasma CXCL8\n(pg/mg total protein)"),
                        c("butyric_acid", "Scalprotectin", "pCXCL8")),
    sex = setNames(c("Female", "Male"), c("F", "M")))) +
  geom_text(aes(x = 1.5, y = ytext, label = stars),
            pdc_sig_pstars, inherit.aes = FALSE, size = 5) +
  geom_segment(aes(x = 1, xend = 2, y = y, yend = y),
               pdc_sig_pstars, inherit.aes = FALSE) +
  theme(panel.grid = element_blank(),
        axis.text = element_text(color = "black"),
        axis.title = element_blank(),
        strip.background = element_rect(fill = "white"))
pdc_sig_plots
```

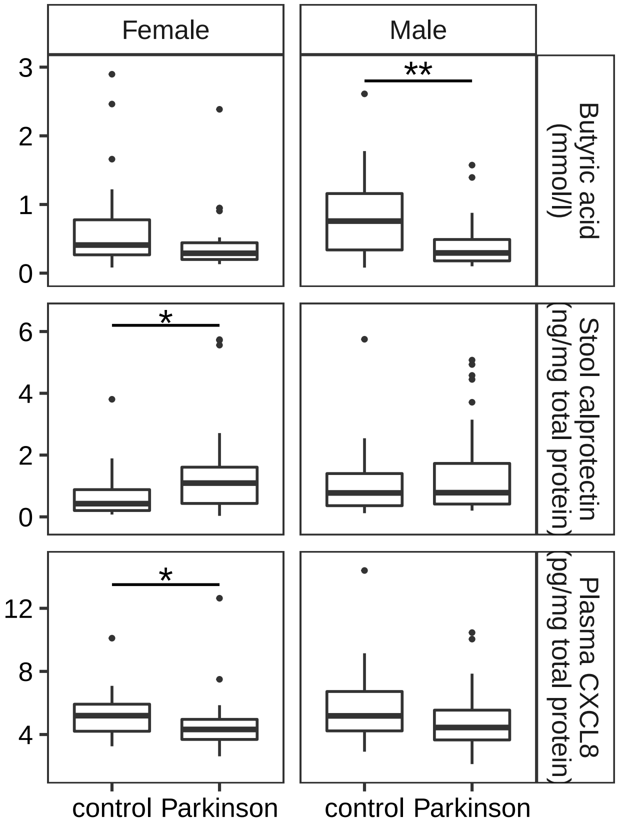


```r
# Export
ggsave(pdc_sig_plots, filename = "Outputs/fig1_pdc_sigs.pdf",
      width = maxwd/2, height = 120, units = "mm",
      device = cairo_pdf)
```

2 Comparisons without microbiota data

2.1 Intercorrelations of inflammatory markers and SCFAs

Fig 2

Run Pearson correlations for pairs of inflammatory markers as well as the SCFAs to test whether they are intercorrelated. Do this for full data as well as control-only and PD-only subsets. Make combined plots of the results.

```
# Full data
ims_cors <- rcorr(as.matrix(
  pdcy_meta[, c(scfa_vars, inf_vars_p, inf_vars_s)]), type = "pearson")

# PD-only data
ims_cors_pd <- rcorr(as.matrix(
  subset(pdcy_meta, Group == "P")[, c(scfa_vars, inf_vars_p, inf_vars_s)]),
  type = "pearson")

# C-only data
ims_cors_c <- rcorr(as.matrix(
  subset(pdcy_meta, Group == "C")[, c(scfa_vars, inf_vars_p, inf_vars_s)]),
  type = "pearson")

# Better variable labels
ims_cor_labels <- varLabelFix(c(scfa_vars, inf_vars_p, inf_vars_s))

custom_corrplot <- function(cmat, pmat){
  ggcorrplot(cmat, type = "upper", p.mat = pmat,
             tl.cex = 5, colors = c("black", "white", "firebrick"),
             insig = "pch", pch = 30) +
    geom_point(aes(shape = factor(pvalue < 0.05)), size = 0.5) +
    scale_shape_manual(values = c(32, 8), name = "p < 0.05",
                       labels = c("no", "yes")) +
    scale_x_discrete(position = "top",
                     labels = ims_cor_labels[1:(length(ims_cor_labels)-1)]) +
    scale_y_discrete(labels = ims_cor_labels[2:length(ims_cor_labels)]) +
    geom_segment(inherit.aes = FALSE,
                 data = data.frame(
                   x = c(0, 0, 7.5, 18.5),
                   y = c(6.5, 17.5, 30.5, 30.5),
                   xend = c(7.5, 18.5, 7.5, 18.5),
                   yend = c(6.5, 17.5, 6.5, 17.5), size = 1),
                 aes(x = x, y = y, xend = xend, yend = yend)) +
    geom_text(inherit.aes = FALSE,
              data = data.frame(
                x = c(4, 10, 22),
                y = c(1, 7, 19),
                label = c("SCFAs", "Plasma markers", "Stool markers")),
              aes(x = x, y = y, label = label),
              hjust = 0, angle = 45, size = 2) +
    theme(axis.text.x = element_text(hjust = 0, vjust = 0.5, color = "black",
                                     angle = 60),
          axis.text.y = element_text(color = "black"),
          axis.ticks = element_line(color = "black"),
```

```
          panel.grid = element_blank(),
          legend.key = element_rect(color = "gray30", size = 0.1),
          legend.key.size = unit(0.3, "cm"),
          legend.box.margin = margin(l = -2, t = 2, unit = "cm"),
          legend.box = "horizontal",
          legend.text = element_text(size = 6),
          legend.title = element_text(size = 7),
          legend.spacing.x = unit(0.1, "cm"))
  }

marker_scfa_cor_plots <- arrangeGrob(
  custom_corrplot(ims_cors$r, ims_cors$P) +
    ggtitle("A. Full data") +
    theme(plot.title = element_text(vjust = -1, hjust = -0.5, size = 10)),
  custom_corrplot(ims_cors_c$r, ims_cors_c$P) +
    ggtitle("B. Control only") +
    theme(plot.title = element_text(vjust = -1, hjust = -0.65, size = 10)),
  custom_corrplot(ims_cors_pd$r, ims_cors_pd$P) +
    ggtitle("C. PD only") +
    theme(plot.title = element_text(vjust = -1, hjust = -0.5, size = 10)),
  ncol = 1)
grid.arrange(marker_scfa_cor_plots)
```

A. Full data

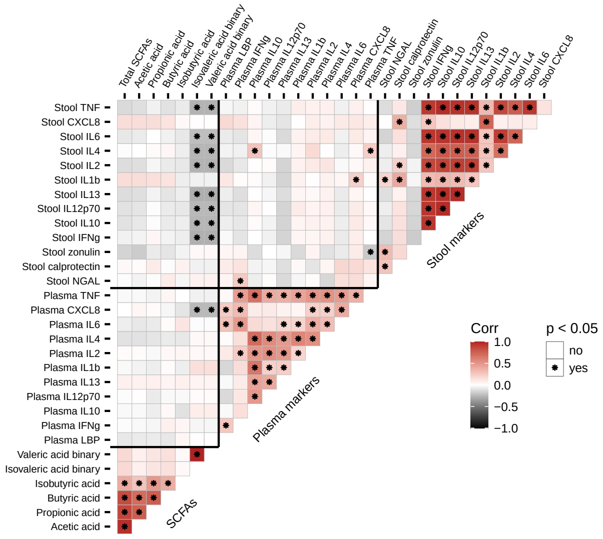

B. Control only

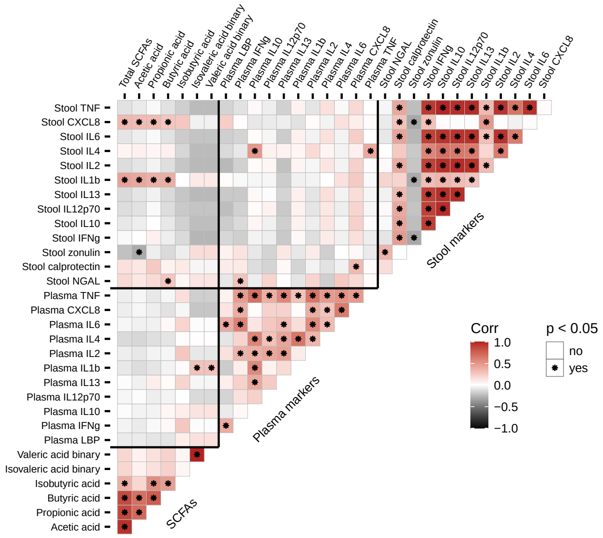

C. PD only

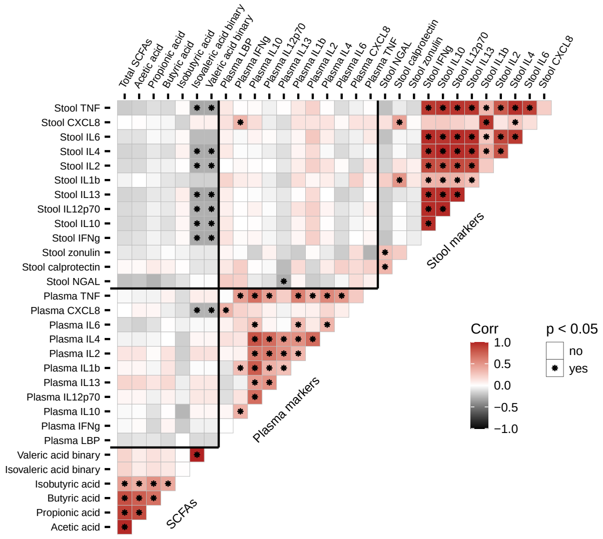

```r
ggsave(marker_scfa_cor_plots, filename = "Outputs/fig2_scfa_marker_cors.pdf",
      width = maxwd/2, height = maxhi, units = "mm", device = cairo_pdf)
```

2.2 PCA with inflammatory markers

Since many of the markers were intercorrelated, use Principal Component Analysis to condense those variables (goal: decrease the number of variables in comparisons and avoid collinearity issues).

2.2.1 Stool

For stool, leave out NGAL, calprotectin, zonulin, IL1b and CXCL8 (which were less strongly intercorrelated with other variables) and include the rest of the variables.

```r
infs_PCA_vars <- inf_vars_s[!(inf_vars_s %in% c("SNGAL", "Scalprotectin", "Szonulin",
                                                "SIL1b", "SCXCL8"))]

# Scale the values
infs_scaled <- scale(pdcy_meta[, infs_PCA_vars])
infs_scaled <- as.data.frame(infs_scaled[1:nrow(infs_scaled), ])

# Calculate PCA
infs_PCA <- prcomp(as.formula(paste("~", paste(infs_PCA_vars, collapse = " + "))),
                   data = infs_scaled, na.action = na.omit)
```

The proportion of variance explained by the PCA axes as a plot:

```r
fviz_screeplot(infs_PCA, ncp = 15) +
  theme_bw(base_size = 9) +
  ylab("Percentage of\nexplained variances") +
  theme(panel.grid.major.x = element_blank())
```

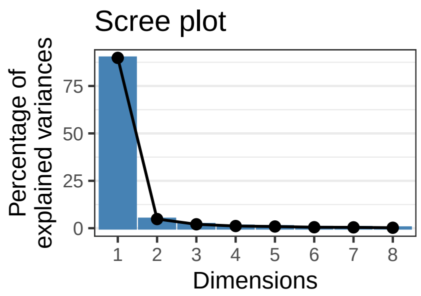

Table of metrics for all principal components:

```r
kable(summary(infs_PCA)$importance, digits = 3, booktabs = TRUE)
```

	PC1	PC2	PC3	PC4	PC5	PC6	PC7	PC8
Standard deviation	2.681	0.622	0.406	0.304	0.268	0.203	0.191	0.140
Proportion of Variance	0.898	0.048	0.021	0.012	0.009	0.005	0.005	0.002
Cumulative Proportion	0.898	0.947	0.967	0.979	0.988	0.993	0.998	1.000

The first PC captured almost 90% of all variance. It's probably enough to just keep the first axis for further analyses.

Plots of the variables contributing to the first three principal components:

```r
fviz_contrib(infs_PCA, choice = "var", axes = 1) + ggtitle("PC1")
```

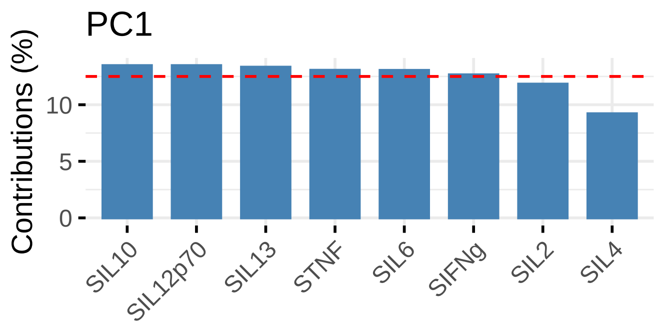

PC1 had significant contributions from most ILs, TNF and IFNg, with IL2 and IL4 falling beneath the reference line.

```
fviz_contrib(infs_PCA, choice = "var", axes = 2) + ggtitle("PC2")
```

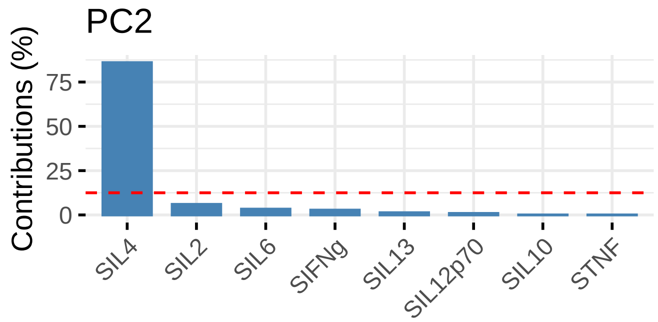

PC2 corresponded to IL4.

```
fviz_contrib(infs_PCA, choice = "var", axes = 3) + ggtitle("PC3")
```

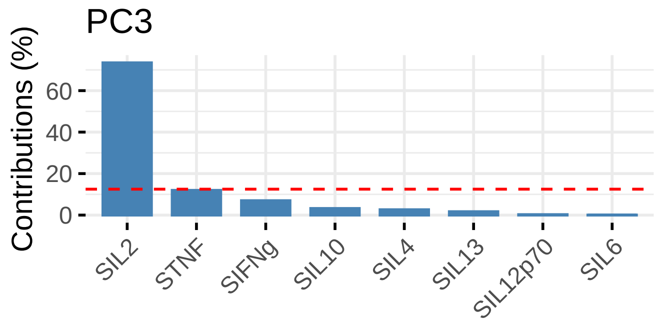

PC3 corresponded to IL2.

Plots of the first three axes, with the PD and control subjects marked:

```
library("ggfortify")

pcaPlotPD <- function(pcdat, df, x, y){
  autoplot(pcdat, data = df, x = x, y = y, colour = "Group", shape = "Group",
           loadings = TRUE, loadings.colour = "gray50", loadings.label = TRUE,
           loadings.label.size = 2.75, scale = 0) +
    theme_bw() +
    coord_fixed() +
    stat_ellipse(aes(group = Group, color = Group, lty = Group), level = 0.9) +
    scale_color_manual(values = c("gray60", "gray30"), name = "Group") +
    scale_shape_manual(values = c(2, 19), name = "Group") +
    scale_linetype_manual(values = c(2, 1), name = "Group") +
```

```
    theme(panel.grid = element_blank())
}

pcaPlotPD(infs_PCA, pdcy_meta[rownames(infs_PCA$x), ], 1, 2)
```

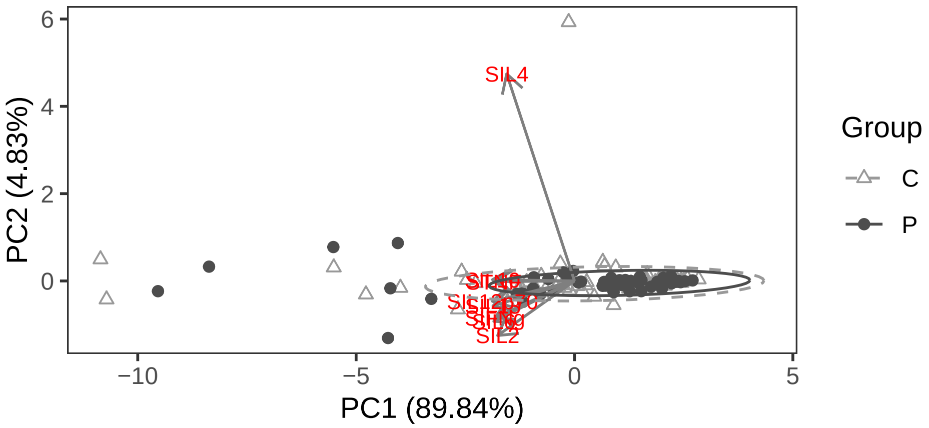


```
pcaPlotPD(infs_PCA, pdcy_meta[rownames(infs_PCA$x), ], 1, 3)
```

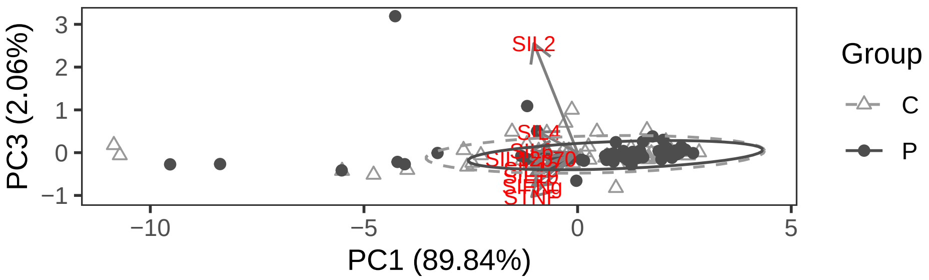


```
pcaPlotPD(infs_PCA, pdcy_meta[rownames(infs_PCA$x), ], 2, 3)
```

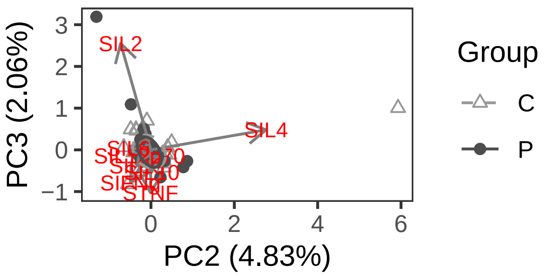

Export PC1 and add to the metadata as "StoolPC" - a summary variable representing IFNg, IL10, IL12p70, IL13, IL6 and TNF. Keep IL4 and IL2 as individual variables.

```
infs_PC1 <- data.frame(infs_PCA$x[, 1])

# Export
write.csv(infs_PC1, "Outputs/infs_pca.csv")

# Add to full data
pdcy_meta$StoolPC <- infs_PC1[rownames(pdcy_meta), ]

# New list of variables of interest for stool (stool markers + PC1)
infs_vars <- c("SNGAL", "Scalprotectin", "Szonulin", "SCXCL8", "SIL1b",
```

```
               "SIL4", "SIL2", "StoolPC")
```

2.2.2 Plasma

For plasma marker PCA, leave out LBP, IFNg, IL6 and CXCL8, which were not as strongly intercorrelated as the rest of the variables, and run the analysis with the remaining variables.

```
infp_PCA_vars <- inf_vars_p[!(inf_vars_p %in% c("pLBP", "pIFNg", "pCXCL8", "pIL6"))]

# Scale the values
infp_scaled <- scale(pdcy_meta[, infp_PCA_vars])
infp_scaled <- as.data.frame(infp_scaled[1:nrow(infp_scaled), ])

# Calculate PCA
infp_PCA <- prcomp(as.formula(paste("~", paste(infp_PCA_vars, collapse = " + "))),
                   data = infp_scaled, na.action = na.omit)
```

The proportion of variance explained by the resulting PCA axes as a plot:

```
fviz_screeplot(infp_PCA, ncp = 15) +
  theme_bw(base_size = 9) +
  ylab("Percentage of\nexplained variances") +
  theme(panel.grid.major.x = element_blank())
```

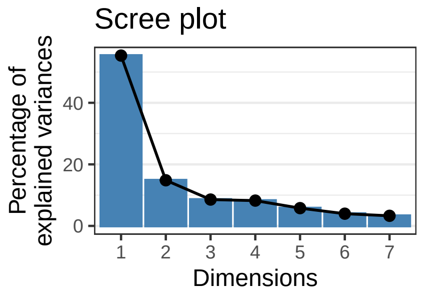

Table of metrics for all principal components:

```
kable(summary(infp_PCA)$importance[1:3, ], digits = 3, booktabs = TRUE)
```

	PC1	PC2	PC3	PC4	PC5	PC6	PC7
Standard deviation	1.968	1.019	0.774	0.759	0.636	0.527	0.480
Proportion of Variance	0.553	0.148	0.086	0.082	0.058	0.040	0.033
Cumulative Proportion	0.553	0.702	0.787	0.870	0.927	0.967	1.000

The situation was not as clear as for the stool variables, but PC1 did explain 55% of all variance. One could consider keeping either just PC1, or the first two PCs.

Plots of the variables contributing to the first four principal components:

```
fviz_contrib(infp_PCA, choice = "var", axes = 1) + ggtitle("PC1")
```

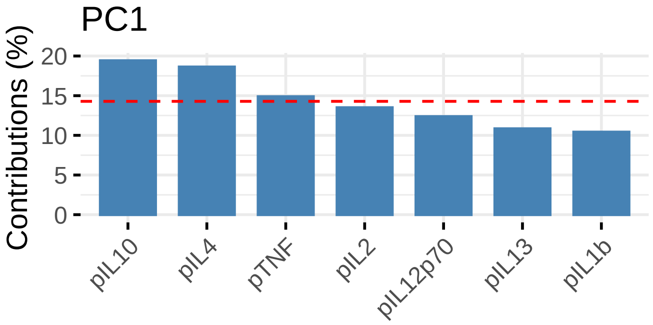

PC1 had contributions mostly from IL10, IL4 and TNF.

```
fviz_contrib(infp_PCA, choice = "var", axes = 2) + ggtitle("PC2")
```

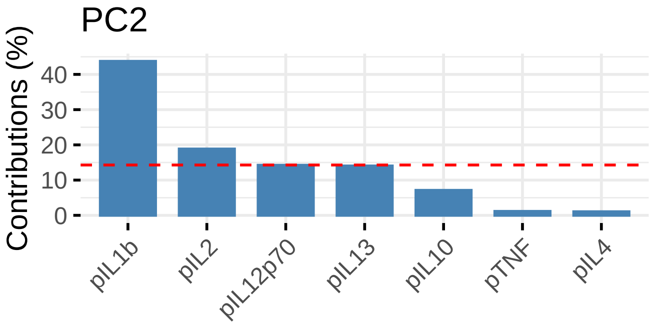

PC2 was mainly IL1b, with minor contributions from IL2, IL12p70 and IL13.

```
fviz_contrib(infp_PCA, choice = "var", axes = 3) + ggtitle("PC3")
```

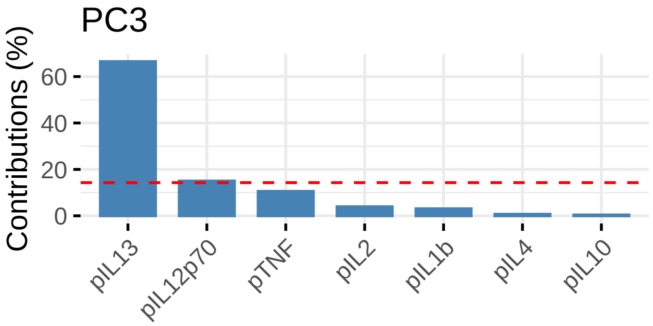

PC3 was mostly IL13 and a minor contribution from IL12p70.

Plot of PC1 and PC2, with the PD and control subjects marked:

```
pcaPlotPD(infp_PCA, pdcy_meta[rownames(infp_PCA$x), ], 1, 2)
```

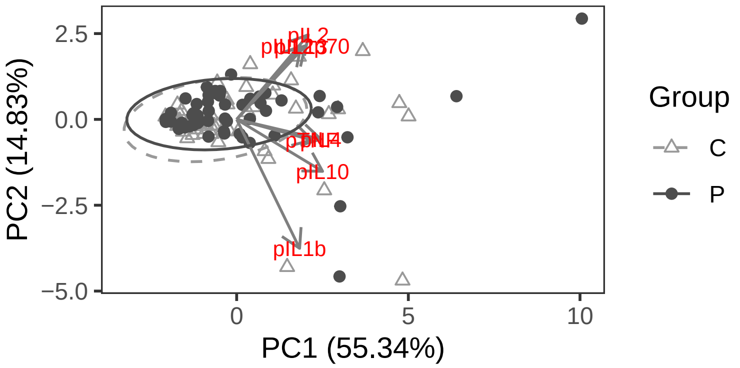

Export the first two axes for downstream use:

```
infp_PCs <- data.frame(infp_PCA$x[, 1:2])

# Export
write.csv(infp_PCs, "Outputs/infp_pca.csv")

# Add to data
infp_PCs <- infp_PCs[rownames(pdcy_meta), ]
pdcy_meta$pPC1 <- infp_PCs$PC1
pdcy_meta$pPC2 <- infp_PCs$PC2

# New list of variables of interest for plasma
infp_vars <- c("pLBP", "pIFNg", "pCXCL8", "pIL6", "pPC1", "pPC2")

# Final list of marker/SCFA variables of interest
inf_scfa_vars <- c(scfa_vars, infs_vars, infp_vars)

# Full list of all marker/SCFA variables (including redundant ones)
inf_scfa_all_vars <- unique(c(inf_scfa_vars, inf_vars_s, inf_vars_p,
                              "valeric_acid", "isovaleric_acid"))
```

2.3 Correlations between clinical variables

To test for correlations between variables, run a correlation matrix for the inflammatory markers, SCFAs, and potential confounding variables that are either numeric or binary factors. Run tests for full data and control-only and PD-only subsets, with the PD-only comparisons including additional variables that are only relevant to PD patients.

```
# Numeric variables
cor_vars <- names(which(sapply(pdcy_meta, is.numeric)))

# Binary variables
cor_vars_b <- names(which(sapply(pdcy_meta, function(x) length(levels(x))) == 2))
# Make binary numeric variables out of the factor ones
cor_vars_b_num <- sapply(pdcy_meta[, cor_vars_b], as.numeric)

# Put together data matrix
cor_data <- cbind(as.matrix(pdcy_meta[, cor_vars]), cor_vars_b_num)
cor_data <- cor_data[, order(colnames(cor_data))]

# Full list of all confounding variables
conf_vars <- colnames(cor_data)
```

```r
# Exclude some non-relevant or too uncommon variables +
# the PD vs control, SCFA and marker variables
conf_vars <- conf_vars[!(conf_vars %in%
                           c("Group", "LED", "DBS",
                             "history_gyn_prolapse",
                             "history_gyn_prolapse_repair",
                             "BPS_defecations_per_week" ,
                             "functional_constipation_criteria_fulfilled",
                             "history_endometriosis",
                             "Rome_III_IBS_possible",
                             "functional_constipation_possible",
                             inf_scfa_all_vars))]

# Trim PD-only relevant confounders (hand-picked) from full data confounders
conf_vars_no_pd <- conf_vars[!(conf_vars %in% unique(c(
  grep("onset", conf_vars, value = TRUE),
  grep("poletti", conf_vars, value = TRUE),
  grep("jankovic", conf_vars, value = TRUE),
  grep("dopa", conf_vars, value = TRUE, ignore.case = TRUE),
  grep("mg", conf_vars, value = TRUE),
  "meds_COMT_inhibitor", "meds_MAO_inhibitor", "Hoehn_and_Yahr_OFF",
  grep("UPDRS", conf_vars, value = TRUE))))]

# Run correlations for full data (trimmed list of variables)
meta_cors <- rcorr(cor_data[, c(conf_vars_no_pd, "Group", inf_scfa_vars)],
                   type = "pearson")

# Run correlations for C-only (trimmed list of variables)
meta_cors_c <- rcorr(cor_data[rownames(subset(pdcy_meta, Group == "C")),
                               c(conf_vars_no_pd, inf_scfa_vars)],
                   type = "pearson")

# Run correlations for PD-only (all variables)
meta_cors_pd <- rcorr(cor_data[rownames(subset(pdcy_meta, Group == "P")),
                               c(conf_vars, inf_scfa_vars)],
                      type = "pearson")
```

2.3.1 SCFAs, markers vs other variables

Look for significant correlations between SCFAs, immune markers and clinical variables.

Fig 3

Plot full data, control-only and PD-only comparisons side by side, excluding the clinical variables that have no correlations with $p < 0.05$ for any comparison in any subset.

```r
# Data frame of p-values
meta_cors_P <- rbind(
  melt(data.frame(meta_cors$P[conf_vars_no_pd, c("Group", inf_scfa_vars)],
             Confounder = conf_vars_no_pd,
             Set = "All subjects")),
  melt(data.frame(meta_cors_c$P[conf_vars_no_pd, inf_scfa_vars],
             Confounder = conf_vars_no_pd,
             Set = "Control subjects")),
  melt(data.frame(meta_cors_pd$P[conf_vars, inf_scfa_vars],
```

```r
            Confounder = conf_vars,
            Set = "PD patients")))
# Add NAs for the variables not in control/full data
# (purely for plotting purposes)
pd_only_vars <- setdiff(conf_vars, conf_vars_no_pd)
meta_cors_P <- rbind(meta_cors_P,
      expand.grid(
        Confounder = pd_only_vars,
        Set = c("All subjects", "Control subjects"),
        variable = inf_scfa_vars,
        value = 1),
      data.frame(
        Confounder = pd_only_vars,
        Set = "All subjects",
        variable = "Group",
        value = 1))

# Data frame of r values
meta_cors_r <- rbind(
  melt(data.frame(meta_cors$r[conf_vars_no_pd, c("Group", inf_scfa_vars)],
            Confounder = conf_vars_no_pd,
            Set = "All subjects")),
  melt(data.frame(meta_cors_c$r[conf_vars_no_pd, inf_scfa_vars],
            Confounder = conf_vars_no_pd,
            Set = "Control subjects")),
  melt(data.frame(meta_cors_pd$r[conf_vars, inf_scfa_vars],
            Confounder = conf_vars,
            Set = "PD patients")))
# Add NAs for the variables not in control/full data
meta_cors_r <- rbind(meta_cors_r,
      expand.grid(
        Confounder = pd_only_vars,
        Set = c("All subjects", "Control subjects"),
        variable = inf_scfa_vars,
        value = NA),
      data.frame(
        Confounder = pd_only_vars,
        Set = "All subjects",
        variable = "Group",
        value = NA))

# Fix labels

# Main variables
inf_scfa_vars2 <- sub(" binary", "",
                             sub(" tool", " ",
                                 varLabelFix(inf_scfa_vars)))
names(inf_scfa_vars2) <- inf_scfa_vars
inf_scfa_vars2 <- c('Group' = "PD vs control", inf_scfa_vars2)

# Confounders
meta_cors_P$Confounder_simple <- meta_cors_P$Confounder
meta_cors_P$Confounder <- mgsub(
```

```r
  gsub("_", " ", capitalize(as.character(meta_cors_P$Confounder))),
  c(" dopa$", "nms", "constip defec.*", "janko", "polet", "pigd", " ar ",
    "meds", " ca ", " OFF", "mg", " average", "lactoseintolerance", "betablocker", "characteristic"),
  c(" levodopa", "NMS", "9-15 sum score", "Janko", "Polet", "PIGD", " AR ",
    "Medication", " Ca ", "", "(mg)", "", "lactose intolerance", "beta blocker", "consistency"),
  ignore.case = TRUE)

meta_cors_P$Confounder <- factor(meta_cors_P$Confounder,
                                 levels = unique(meta_cors_P$Confounder))
# Better label for the tobacco variable
levels(meta_cors_P$Confounder)[grep("Tobacco", levels(meta_cors_P$Confounder))] <-
  "Smoking (>100 cigarettes in life)"

# Same fixes to the df with r values
meta_cors_r$Confounder <- meta_cors_P$Confounder

# Separate subsets per type of variable

# SCFAs
scfa_cor_sigs <- names(which(table(
  subset(meta_cors_P, variable %in% scfa_vars)$Confounder,
  subset(meta_cors_P, variable %in% scfa_vars)$value < 0.05)[,2]>0))
meta_cors_sc_P <- subset(meta_cors_P, Confounder %in% scfa_cor_sigs &
                           variable %in% c("Group", scfa_vars))
meta_cors_sc_P$value <- meta_cors_sc_P$value < 0.05
meta_cors_sc_r <- subset(meta_cors_r, Confounder %in% scfa_cor_sigs &
                           variable %in% c("Group", scfa_vars))

# Plasma markers
p_cor_sigs <- names(which(table(
  subset(meta_cors_P, variable %in% infp_vars)$Confounder,
  subset(meta_cors_P, variable %in% infp_vars)$value < 0.05)[,2]>0))
meta_cors_pl_P <- subset(meta_cors_P, Confounder %in% p_cor_sigs &
                           variable %in% c("Group", infp_vars))
meta_cors_pl_P$value <- meta_cors_pl_P$value < 0.05
meta_cors_pl_r <- subset(meta_cors_r, Confounder %in% p_cor_sigs &
                           variable %in% c("Group", infp_vars))

# Stool markers
s_cor_sigs <- names(which(table(
  subset(meta_cors_P, variable %in% infs_vars)$Confounder,
  subset(meta_cors_P, variable %in% infs_vars)$value < 0.05)[,2]>0))
meta_cors_st_P <- subset(meta_cors_P, Confounder %in% s_cor_sigs &
                           variable %in% c("Group", infs_vars))
meta_cors_st_P$value <- meta_cors_st_P$value < 0.05
meta_cors_st_r <- subset(meta_cors_r, Confounder %in% s_cor_sigs &
                           variable %in% c("Group", infs_vars))

# Plotting function
custom_corrplot2 <- function(cmat, pmat){
  ggplot(cmat, aes(x = variable, y = Confounder,
                           fill = value)) +
  geom_tile(color = "gray30") +
```

```r
  theme_bw(base_size = 6) +
  facet_grid(~Set, space = "free", scales = "free", switch = "x",
             labeller = labeller(Set = setNames(c("All subjects", "Control\nsubjects",
                                                  "PD patients"),
                                            c("All subjects", "Control subjects",
                                              "PD patients")))) +
  scale_x_discrete(position = "top", labels = inf_scfa_vars2) +
  xlab(NULL) + ylab(NULL) +
  scale_fill_gradient2(low = "gray10", mid = "white",
                       high = "firebrick", name = "Corr",
                       limits = c(-1, 1), na.value = "white") +
  geom_point(data = pmat,
             aes(x = variable, y = Confounder,
                 shape = factor(value)),
             inherit.aes = FALSE, size = 0.75, color = "black") +
  scale_shape_manual(values = c(32, 8), name = "p < 0.05",
                     labels = c("no", "yes")) +
  theme(axis.text = element_text(color = "black"),
        axis.text.x.top = element_text(angle = 65,
                                       hjust = 0, vjust = 0),
        axis.ticks = element_line(color = "black"),
        legend.key.size = unit(0.2, "cm"),
        plot.margin = margin(l = 1, r = 0.5, b = 1, unit = "mm"),
        legend.key = element_rect(color = "gray30", size = 0.1),
        panel.grid = element_blank(),
        aspect.ratio = 1)
}

# Make plots
# (title positions adjusted manually by trial and error)
clin_cors_plots <- grid.arrange(
  custom_corrplot2(meta_cors_sc_r, meta_cors_sc_P) +
    ggtitle("A") +
    theme(plot.title = element_text(face = "bold", size = 11,
                                    vjust = -1, hjust = -0.51)),
  custom_corrplot2(meta_cors_pl_r, meta_cors_pl_P) +
    ggtitle("B") +
    theme(plot.title = element_text(face = "bold", size = 11,
                                    vjust = -1, hjust = -0.52)),
  custom_corrplot2(meta_cors_st_r, meta_cors_st_P) +
    ggtitle("C") +
    theme(plot.title = element_text(face = "bold", size = 11,
                                    vjust = -1, hjust = -0.3)),
  ncol = 1, heights = c(0.8, 1, 1))
```

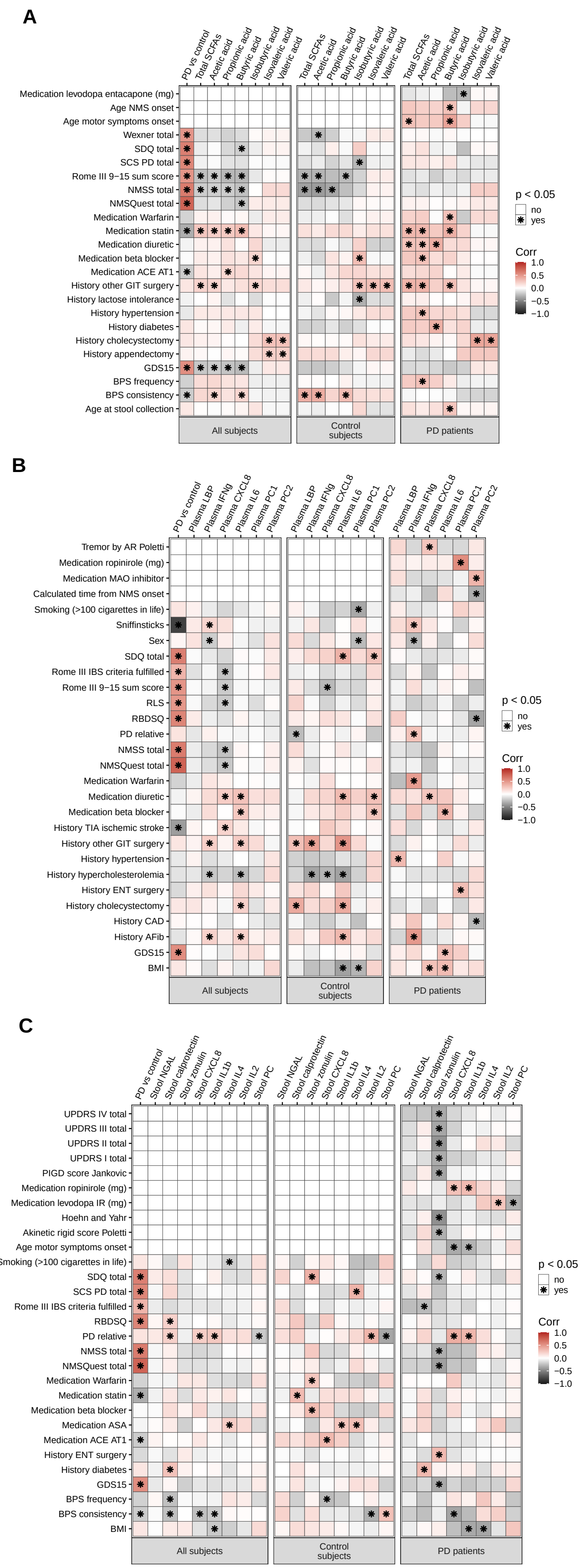
A
PD vs control
Total SCFAs
Acetic acid
Propionic acid
Butyric acid
Isobutyric acid
Isovaleric acid
Valeric acid
Medication levodopa entacapone (mg)
Age NMS onset
Age motor symptoms onset
Wexner total
SDQ total
SCS PD total
Rome III 9–15 sum score
NMSS total
NMSQuest total
Medication Warfarin
Medication statin
Medication diuretic
Medication beta blocker
Medication ACE AT1
History other GIT surgery
History lactose intolerance
History hypertension
History diabetes
History cholecystectomy
History appendectomy
GDS15
BPS frequency
BPS consistency
Age at stool collection
All subjects
Control subjects
PD patients
p < 0.05
no
yes
Corr
1.0
0.5
0.0
−0.5
−1.0
B
PD vs control
Plasma LBP
Plasma IFNg
Plasma CXCL8
Plasma IL6
Plasma PC1
Plasma PC2
Tremor by AR Poletti
Medication ropinirole (mg)
Medication MAO inhibitor
Calculated time from NMS onset
Smoking (>100 cigarettes in life)
Sniffinsticks
Sex
SDQ total
Rome III IBS criteria fulfilled
Rome III 9–15 sum score
RLS
RBDSQ
PD relative
NMSS total
NMSQuest total
Medication Warfarin
Medication diuretic
Medication beta blocker
History TIA ischemic stroke
History other GIT surgery
History hypertension
History hypercholesterolemia
History ENT surgery
History cholecystectomy
History CAD
History AFib
GDS15
BMI
All subjects
Control subjects
PD patients
p < 0.05
no
yes
Corr
1.0
0.5
0.0
−0.5
−1.0
C
PD vs control
Stool NGAL
Stool calprotectin
Stool zonulin
Stool CXCL8
Stool IL1b
Stool IL4
Stool IL2
Stool PC
UPDRS IV total
UPDRS III total
UPDRS II total
UPDRS I total
PIGD score Jankovic
Medication ropinirole (mg)
Medication levodopa IR (mg)
Hoehn and Yahr
Akinetic rigid score Poletti
Age motor symptoms onset
Smoking (>100 cigarettes in life)
SDQ total
SCS PD total
Rome III IBS criteria fulfilled
RBDSQ
PD relative
NMSS total
NMSQuest total
Medication Warfarin
Medication statin
Medication beta blocker
Medication ASA
Medication ACE AT1
History ENT surgery
History diabetes
GDS15
BPS frequency
BPS consistency
BMI
All subjects
Control subjects
PD patients
p < 0.05
no
yes
Corr
1.0
0.5
0.0
−0.5
−1.0

```r
# Export
ggsave(clin_cors_plots, filename = "Outputs/fig3_clin_cors.pdf",
      width = maxwd/2, height = maxhi*0.9, units = "mm",
      device = cairo_pdf)
```

Additional file 4

Scatterplots for the most interesting pairs of variables:

```r
# Basic scatterplot function
basic_scatter <- function(df, xvar, yvar){
  ggplot(df, aes_string(x = xvar, y = yvar,
                        color = "Group", shape = "Group")) +
    geom_point(size = 0.5) +
    scale_color_manual(values = c("gray50", "black"),
                       labels = c("control", "Parkinson")) +
    scale_shape_manual(values = c(16, 17),
                       labels = c("control", "Parkinson")) +
    theme_bw(base_size = 4.5) +
    theme(panel.grid = element_blank(),
          axis.text = element_text(color = "black"),
          aspect.ratio = 1)
}

# Basic boxplot for categorical variables
basic_box <- function(df, xvar, yvar){
  ggplot(df, aes_string(x = xvar, y = yvar, color = "Group")) +
    geom_boxplot(outlier.size = 0.5, lwd = 0.3) +
    scale_x_discrete(labels = c("no", "yes")) +
    scale_color_manual(values = c("gray50", "black"),
                       labels = c("control", "Parkinson")) +
    theme_bw(base_size = 4.5) +
    theme(panel.grid = element_blank(),
          axis.text = element_text(color = "black"),
          aspect.ratio = 1)
}

# Basic scatterplot function (PD only)
basic_scatter_pd <- function(df, xvar, yvar){
  df <- subset(df, Group == "P")
  ggplot(df, aes_string(x = xvar, y = yvar)) +
    geom_point(shape = 17, size = 0.5) +
    theme_bw(base_size = 4.5) +
    theme(panel.grid = element_blank(),
          axis.text = element_text(color = "black"),
          aspect.ratio = 1)
}

# Basic boxplot function (PD only)
basic_box_pd <- function(df, xvar, yvar){
  df <- subset(df, Group == "P")
  ggplot(df, aes_string(x = xvar, y = yvar)) +
    geom_boxplot(outlier.size = 0.5, lwd = 0.3) +
    scale_x_discrete(labels = c("no", "yes")) +
    theme_bw(base_size = 4.5) +
```

```
    theme(panel.grid = element_blank(),
          axis.text = element_text(color = "black"),
          aspect.ratio = 1)
}

# Get all pairs of significant correlations from full data and PD-only comparisons
sig_cors <- subset(meta_cors_P, value < 0.05 & variable != "Group" &
                     Set != "Control subjects")
# Rearrange
sig_cors <- dcast(sig_cors, variable + Confounder + Confounder_simple ~ Set)
# Trim to PD-related confounders of interest
sig_cors <- subset(sig_cors,
                   Confounder_simple %in% c("age_NMS_onset", "NMSS_total",
                                            "Rome_III_constip_defec_sumscore_9.15",
                                            "Rome_III_IBS_criteria_fulfilled",
                                            "GDS15",
                                            "UPDRS_III_total_OFF", "Hoehn_and_Yahr_OFF",
                                            "SDQ_total", "age_motor_symptoms_onset",
                                            "meds_statin", "history_TIA_ischemic_stroke"))
# Exclude propionic and acetic acid, since these
# tend to be similar to the total SCFAs and butyric acid
sig_cors <- subset(sig_cors, variable != "propionic_acid" &
                     variable != "acetic_acid")

# Plot variables significant for full data
conf_cors_full <- apply(subset(sig_cors, `All subjects` < 0.05), 1,
                        function(x){
                          if(is.numeric(pdcy_meta[, x["Confounder_simple"]])) {
                            basic_scatter(pdcy_meta, x["Confounder_simple"],
                                          x["variable"]) +
                              xlab(x["Confounder"]) +
                              ylab(inf_scfa_vars2[x["variable"]]) +
                              theme(legend.position = "none")
                          } else {
                            basic_box(
                              subset(pdcy_meta, !is.na(
                                pdcy_meta[, as.character(
                                  as.matrix(x["Confounder_simple"]))])),
                              x["Confounder_simple"],
                              x["variable"]) +
                              xlab(x["Confounder"]) +
                              ylab(inf_scfa_vars2[x["variable"]]) +
                              theme(legend.position = "none")
                          }})

# Legend-grabbing function
gLegend <- function(a.gplot){
  tmp <- ggplot_gtable(ggplot_build(a.gplot))
  leg <- which(sapply(tmp$grobs, function(x) x$name) == "guide-box")
  legend <- tmp$grobs[[leg]]
  legend
}
```

```r
# Get legend for the full-data plots
clin_plot_legend <- gLegend(
      basic_scatter(pdcy_meta, "GDS15", "total_SCFAs") +
        geom_point(size = 1.5) +
        theme_bw(base_size = 6) +
        theme(legend.background = element_rect(color = "black", size = 0.1),
              legend.key.height = unit(3, "mm"),
              legend.position = "bottom")
)

grid.arrange(
  do.call("arrangeGrob",
          c(conf_cors_full[1:(length(conf_cors_full)-1)], ncol = 4)),
  arrangeGrob(clin_plot_legend, conf_cors_full[[length(conf_cors_full)]],
              nrow = 1, widths = c(3, 1)),
  nrow = 2, heights = c(3, 1))
```

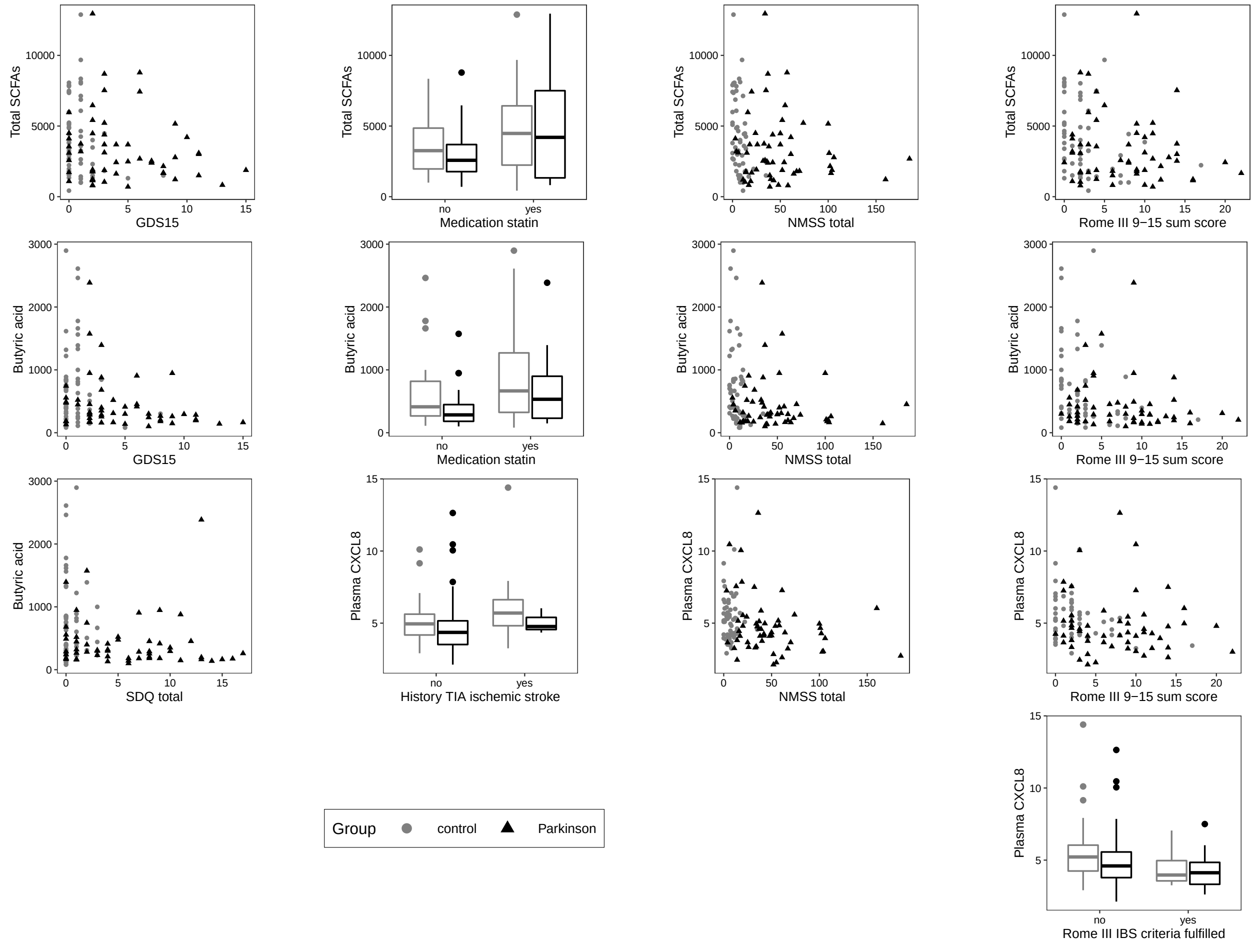


```r
# Plot variables significant for PD-only
conf_cors_pd <- apply(subset(sig_cors, `PD patients`< 0.05 &
                                      is.na(`All subjects`)), 1,
                        function(x){
                          if(is.numeric(pdcy_meta[, x["Confounder_simple"]])) {
                            basic_scatter_pd(pdcy_meta, x["Confounder_simple"],
                                             x["variable"]) +
```

```
                              xlab(x["Confounder"]) +
                              ylab(inf_scfa_vars2[x["variable"]])
                          } else {
                            basic_box_pd(
                              pdcy_meta, x["Confounder_simple"],
                              x["variable"]) +
                              xlab(x["Confounder"]) +
                              ylab(inf_scfa_vars2[x["variable"]])
                          }})

do.call("grid.arrange", c(conf_cors_pd, ncol = 4))
```

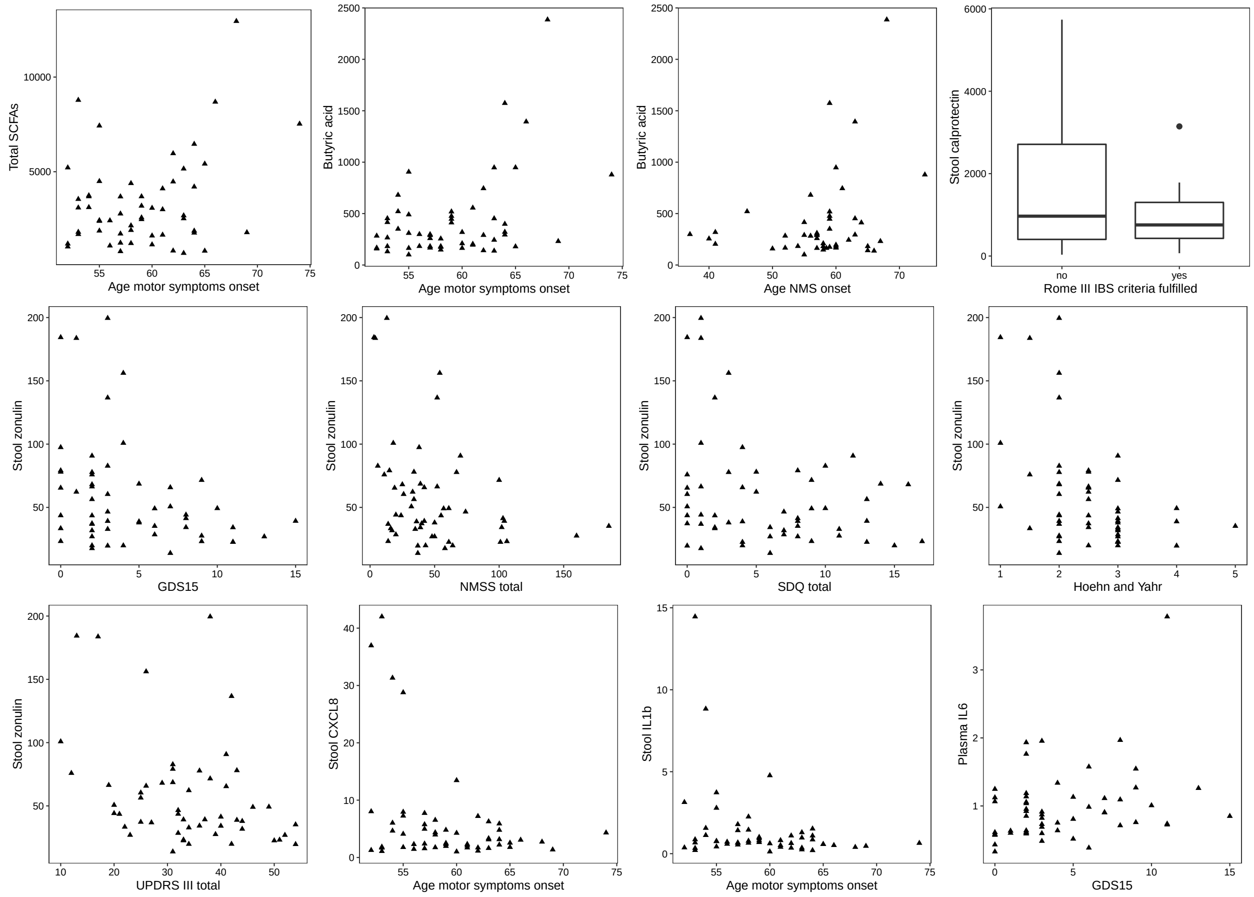


```
# Export both figures together

library("grid")

pdf("Outputs/add4_clin_cors_scatters.pdf", width = 4.5, height = 7)
grid.arrange(
  textGrob("A", x = unit(0.025, "npc"), y = unit(0.4, "npc"),
           gp = gpar(fontface = "bold", fontsize = 12)),
  arrangeGrob(
    do.call("arrangeGrob",
            c(conf_cors_full[1:(length(conf_cors_full)-1)], ncol = 4)),
    arrangeGrob(clin_plot_legend, conf_cors_full[[length(conf_cors_full)]],
                nrow = 1, widths = c(3, 1)),
```

```
    nrow = 2, heights = c(3, 1)),
  textGrob("B", x = unit(0.025, "npc"), y = unit(0.4, "npc"),
           gp = gpar(fontface = "bold", fontsize = 12)),
  do.call("arrangeGrob", c(conf_cors_pd, ncol = 4)),
  ncol = 1, heights = c(0.1, 1.35, 0.1, 1))
dev.off()
```

Linear regression corrected for age and/or time from disease onset

The PD-related correlations are interesting, but should be corrected for some confounding factors, mainly age and disease duration. Prior to corrections, check the potential confounding variables for collinearity.

Butyric acid ~ age motor symptoms onset:

```
ggpairs(subset(
    pdcy_meta, Group == "P")[,c("age_motor_symptoms_onset",
                                "age_NMS_onset",
                                "age_at_stool_collection",
                                "calculated_time_from_motor_onset")]) +
    theme_bw(base_size = 8) +
    theme(panel.grid = element_blank())
```

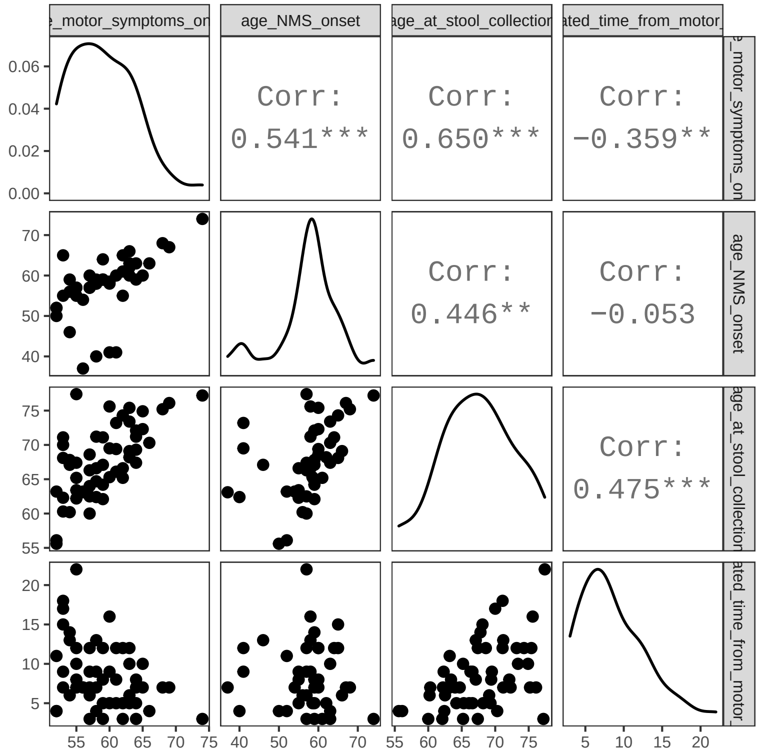

All three variables are somewhat correlated; use the less collinear one (calculated time from motor onset) for correction.

Model for butyric acid ~ time from motor onset: run and plot residuals.

```
buty_motors_regr <- lm(butyric_acid ~ age_motor_symptoms_onset +
                    calculated_time_from_motor_onset,
                data = subset(pdcy_meta, Group == "P"))

autoplot(buty_motors_regr, label.size = 2, nrow = 1) +
```

```r
  theme_bw(base_size = 8)
```

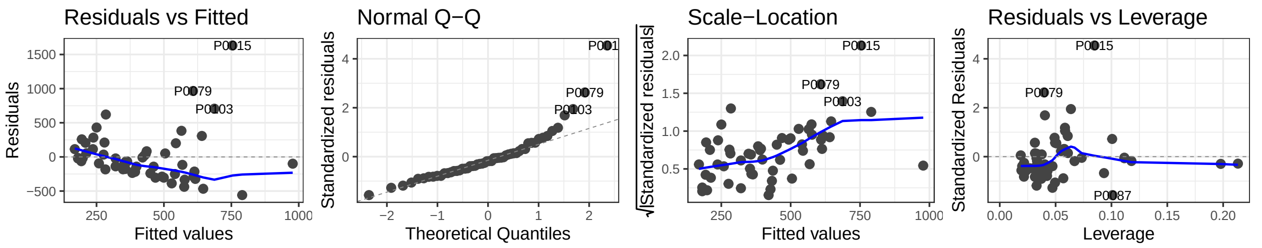

The diagnostic plots don't look very good - log-transform butyric acid and plot again:

```r
buty_motors_regr <- lm(log(butyric_acid) ~ age_motor_symptoms_onset +
                        calculated_time_from_motor_onset,
                    data = subset(pdcy_meta, Group == "P"))

autoplot(buty_motors_regr, label.size = 2, nrow = 1) +
  theme_bw(base_size = 8)
```

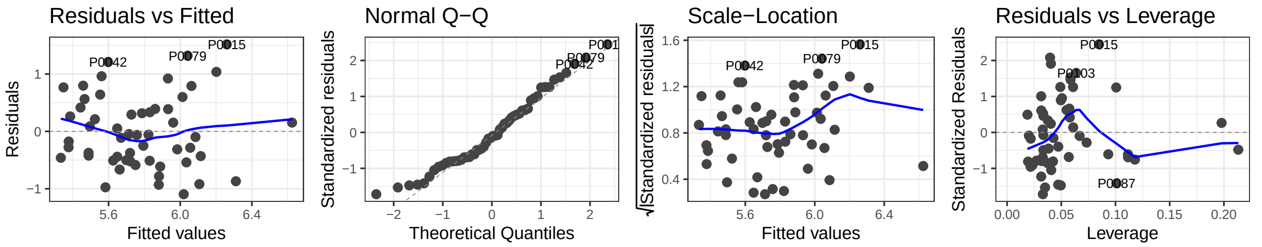

This looks better; use the log-transformed variable. Show model summary:

```r
summary(buty_motors_regr)
```

```
##
## Call:
## lm(formula = log(butyric_acid) ~ age_motor_symptoms_onset + calculated_time_from_motor_onset,
##     data = subset(pdcy_meta, Group == "P"))
##
## Residuals:
##      Min       1Q   Median       3Q      Max
## -1.09495 -0.49156 -0.07992  0.39073  1.51757
##
## Coefficients:
##                                  Estimate Std. Error t value Pr(>|t|)
## (Intercept)                       2.89638    1.24023   2.335   0.0235 *
## age_motor_symptoms_onset          0.05098    0.01958   2.604   0.0121 *
## calculated_time_from_motor_onset -0.01471    0.02253  -0.653   0.5166
## ---
## Signif. codes:  0 '***' 0.001 '**' 0.01 '*' 0.05 '.' 0.1 ' ' 1
##
## Residual standard error: 0.6478 on 51 degrees of freedom
##   (1 observation deleted due to missingness)
## Multiple R-squared:  0.1595, Adjusted R-squared:  0.1265
## F-statistic: 4.838 on 2 and 51 DF,  p-value: 0.01192
```

Age motor symptoms onset is still significant after correction.

What about age NMS onset (still using log-transformed butyric acid)?

```r
summary(lm(log(butyric_acid) ~ age_NMS_onset +
             calculated_time_from_motor_onset,
           data = subset(pdcy_meta, Group == "P")))
```

```
##
## Call:
## lm(formula = log(butyric_acid) ~ age_NMS_onset + calculated_time_from_motor_onset,
##     data = subset(pdcy_meta, Group == "P"))
##
## Residuals:
##     Min      1Q  Median      3Q     Max
## -1.0909 -0.5257 -0.1332  0.3247  1.7834
##
## Coefficients:
##                                 Estimate Std. Error t value Pr(>|t|)
## (Intercept)                      4.88412    0.84704   5.766  9.3e-07 ***
## age_NMS_onset                    0.01996    0.01399   1.427    0.161
## calculated_time_from_motor_onset -0.03539    0.02517  -1.406    0.167
## ---
## Signif. codes:  0 '***' 0.001 '**' 0.01 '*' 0.05 '.' 0.1 ' ' 1
##
## Residual standard error: 0.687 on 41 degrees of freedom
##   (11 observations deleted due to missingness)
## Multiple R-squared:  0.09366,    Adjusted R-squared:  0.04945
## F-statistic: 2.119 on 2 and 41 DF,  p-value: 0.1332
```

Age NMS onset is not significant in this model.

Stool zonulin and PD-related variables + potential confounders: check for collinearity:

```r
  ggpairs(subset(
    pdcy_meta, Group == "P")[,c("age_motor_symptoms_onset",
                                "age_NMS_onset",
                                "age_at_stool_collection",
                                "calculated_time_from_motor_onset",
                                "calculated_time_from_nms_onset",
                                "UPDRS_III_total_OFF",
                                "Hoehn_and_Yahr_OFF",
                                "SDQ_total",
                                "NMSS_total",
                                "GDS15")]) +
    theme_bw(base_size = 8) +
    theme(panel.grid = element_blank())
```

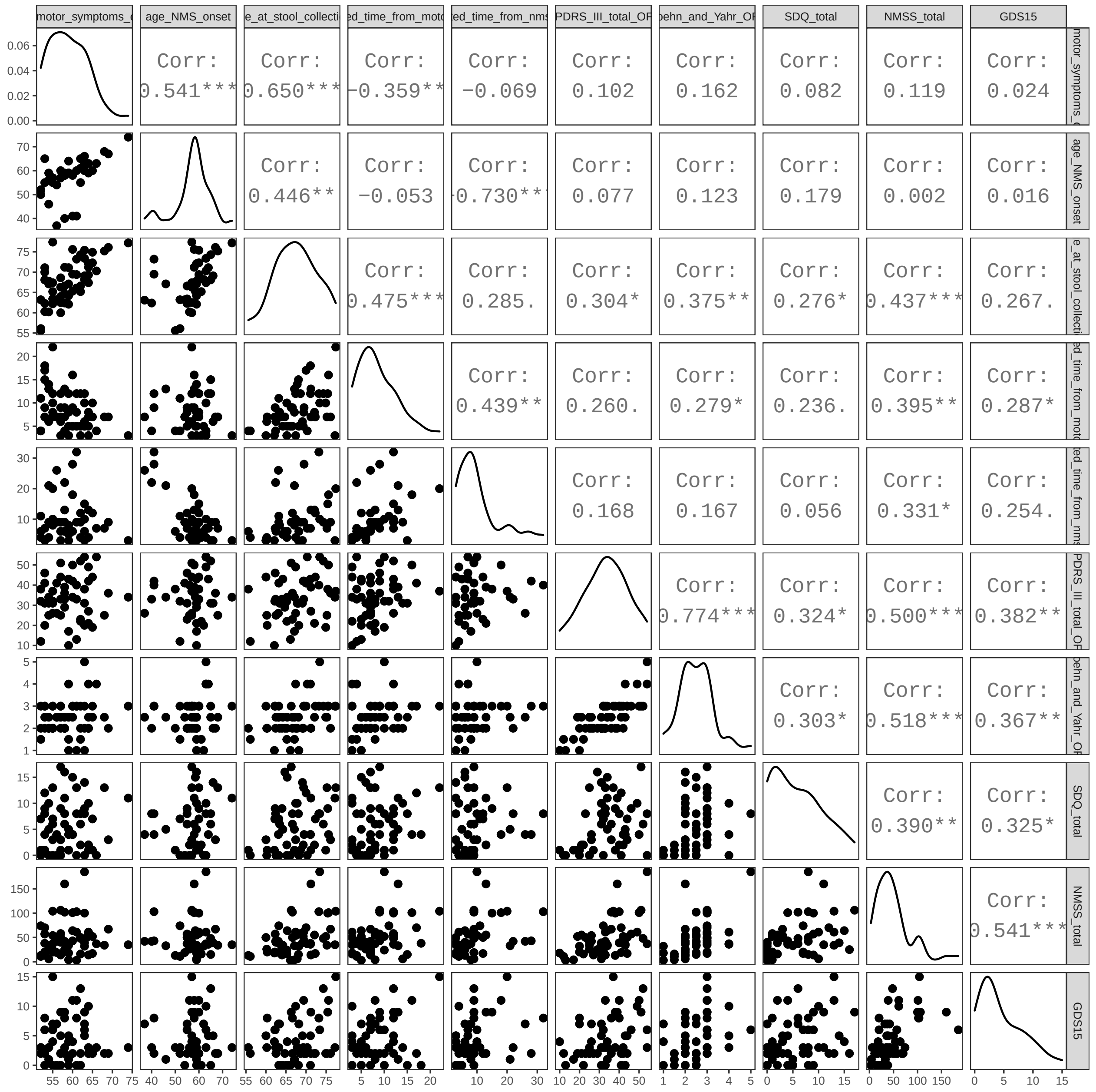

The variables of interest are slightly correlated with the age variables, but not very highly.

Check residual plots for one example model:

```
autoplot(lm(Szonulin ~ GDS15 +
              calculated_time_from_motor_onset,
           data = subset(pdcy_meta, Group == "P")),
         label.size = 2, nrow = 1) +
  theme_bw(base_size = 8)
```

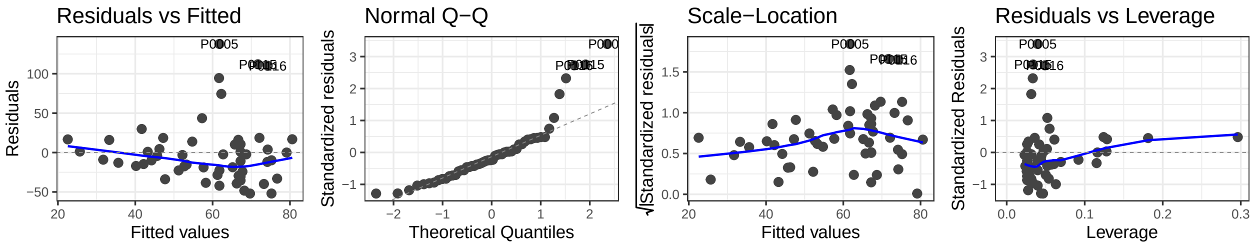

Similar issues to above; try with log transformation of zonulin:

```
autoplot(lm(log(Szonulin) ~ GDS15 +
              calculated_time_from_motor_onset,
            data = subset(pdcy_meta, Group == "P")),
         label.size = 2, nrow = 1) +
  theme_bw(base_size = 8)
```

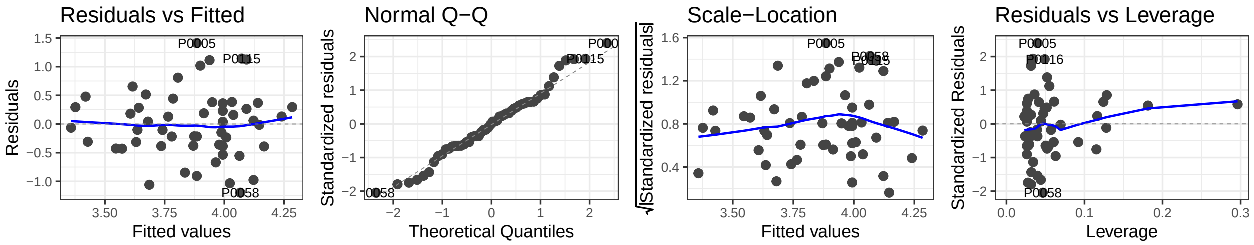

Though not perfect, this is better; use log-transformed zonulin in all models.

Run regression for all variables of interest with log-transformed zonulin, corrected for calculated time from motor onset:

```
szonulin_motors_regr <- as.data.frame(matrix(ncol = 4, nrow = 0))
colnames(szonulin_motors_regr) <- c("Model", "pValModel",
                                    "pValVar", "pValConf")

for(i in c("UPDRS_III_total_OFF", "Hoehn_and_Yahr_OFF",
           "SDQ_total", "NMSS_total", "GDS15")){
  m <- as.formula(paste("log(Szonulin) ~", i,
                        "+calculated_time_from_motor_onset"))
  res <- summary(lm(m, subset(pdcy_meta, Group == "P")))
  szonulin_motors_regr <- rbind(szonulin_motors_regr,
                                data.frame(Model = deparse(m),
                                           pValModel =
                                             unname(pf(res$fstatistic[1],
                                                       res$fstatistic[2],
                                                       res$fstatistic[3],
                                                       lower.tail=FALSE)),
                                           pValVar1 = res$coefficients[2, 4],
                                           pValVar2 = res$coefficients[3, 4]))
}

# Show results
kable_styling(kable(szonulin_motors_regr, digits = 4,
                    booktabs = TRUE, linesep = "",
                    row.names = FALSE), font_size = 8)
```

Model	pValModel	pValVar1	pValVar2
log(Szonulin) ~ UPDRS_III_total_OFF + calculated_time_from_motor_onset	0.0166	0.0045	0.6006
log(Szonulin) ~ Hoehn_and_Yahr_OFF + calculated_time_from_motor_onset	0.0068	0.0017	0.5023
log(Szonulin) ~ SDQ_total + calculated_time_from_motor_onset	0.1517	0.0534	0.6846
log(Szonulin) ~ NMSS_total + calculated_time_from_motor_onset	0.0465	0.0137	0.3459
log(Szonulin) ~ GDS15 + calculated_time_from_motor_onset	0.0309	0.0087	0.4725

All five variables remain significant after correction (and calculated time from motor onset is not significant).

Same, but corrected for age from non-motor symptom onset instead:

```
szonulin_nms_regr <- as.data.frame(matrix(ncol = 4, nrow = 0))
colnames(szonulin_nms_regr) <- c("Model", "pValModel",
                                 "pValVar", "pValConf")

for(i in c("UPDRS_III_total_OFF", "Hoehn_and_Yahr_OFF",
           "SDQ_total", "NMSS_total", "GDS15")){
    m <- as.formula(paste("log(Szonulin) ~", i,
                          "+calculated_time_from_nms_onset"))
    res <- summary(lm(m, subset(pdcy_meta, Group == "P")))
    szonulin_nms_regr <- rbind(szonulin_nms_regr,
                               data.frame(Model = deparse(m),
                                          pValModel =
                                            unname(pf(res$fstatistic[1],
                                                      res$fstatistic[2],
                                                      res$fstatistic[3],
                                                      lower.tail=FALSE)),
                                pValVar1 = res$coefficients[2, 4],
                                pValVar2 = res$coefficients[3, 4]))
}

# Show results
kable_styling(kable(szonulin_nms_regr, digits = 4,
                    booktabs = TRUE, linesep = "",
                    row.names = FALSE), font_size = 8)
```

Model	pValModel	pValVar1	pValVar2
log(Szonulin) ~ UPDRS_III_total_OFF + calculated_time_from_nms_onset	0.0133	0.0079	0.3955
log(Szonulin) ~ Hoehn_and_Yahr_OFF + calculated_time_from_nms_onset	0.0159	0.0096	0.3902
log(Szonulin) ~ SDQ_total + calculated_time_from_nms_onset	0.1762	0.1473	0.2710
log(Szonulin) ~ NMSS_total + calculated_time_from_nms_onset	0.0429	0.0270	0.6807
log(Szonulin) ~ GDS15 + calculated_time_from_nms_onset	0.0821	0.0575	0.4954

3 Comparisons with microbiota data

Import microbiota data and combine with the metadata:

```
# Import
pd_otus <- as.matrix(read.csv("Inputs/pdfu_16s_OTU_counts.csv", row.names = 1))
pd_tax <- as.matrix(read.csv("Inputs/pdfu_16s_taxonomy.csv", row.names = 1))

# Trim the OTU table to follow-up only
pd_otus <- pd_otus[grep("N", rownames(pd_otus)), ]
```

```r
# Remove timepoint identifier from row names
rownames(pd_otus) <- sub("N", "", rownames(pd_otus))

# Match to the samples that are in the metadata
pd_otus <- pd_otus[rownames(pdcy_meta), ]

# Combine data into a phyloseq object
pdcy_phy <- phyloseq(otu_table(t(pd_otus), taxa_are_rows = TRUE),
                     tax_table(pd_tax),
                     sample_data(pdcy_meta))

# Trim out OTUs that have no reads with this subset of samples
pdcy_phy <- subset_taxa(pdcy_phy, taxa_sums(pdcy_phy) > 0)
```

Make summarized objects on different taxonomic levels:

```r
# Function for merging phyloseq data to different taxonomic levels:
collapseTaxLevel <- function(phylo_obj, level){

  collapseOtuTable <- function(phylo_obj, level){
    tax <- as.data.frame(as(tax_table(phylo_obj), "matrix"))
    otu <- as.data.frame(as(otu_table(phylo_obj), "matrix"))
    otu[, level] <- tax[, level]
    otu_collapsed <- melt(otu, id = (level))
    otu_collapsed <- acast(otu_collapsed,
                           as.formula(paste0(level, "~variable")),  sum)
    otu_collapsed <- otu_collapsed[order(rownames(otu_collapsed)), ]
    return(otu_collapsed)
  }

  levelNum <- grep(level, colnames(tax_table(phylo_obj)))

  tax <- as(tax_table(phylo_obj), "matrix")
  tax <- apply(tax, 2, function(x) gsub(".*_unclassified", "unclassified", x))
  phylo_obj <- phyloseq(otu_table(phylo_obj),
                        sample_data(phylo_obj),
                        tax_table(tax))

  otu_collapsed <- collapseOtuTable(phylo_obj, level)

  tax <- as(tax_table(phylo_obj), "matrix")
  tax_collapsed <- unique(tax[, 1:levelNum])

  tax_collapsed <- tax_collapsed[-grep("unclassified", tax_collapsed[,levelNum]),]
  tax_collapsed <- rbind(tax_collapsed, rep("unclassified", (levelNum)))

  rownames(tax_collapsed) <- tax_collapsed[, level]
  tax_collapsed <- tax_collapsed[order(rownames(tax_collapsed)), ]

  new_phylo_obj <- phyloseq(otu_table(otu_collapsed,  taxa_are_rows = TRUE),
                            tax_table(tax_collapsed),
                            sample_data(phylo_obj))
  return(new_phylo_obj)
}
```

```r
# Make genus and family level summarized phyloseq objects:
pdcy_phy_gen <- collapseTaxLevel(pdcy_phy, "Genus")
pdcy_phy_fam <- collapseTaxLevel(pdcy_phy, "Family")

# Subsets of data for downstream comparisons:

# PD-only
pdcy_phy_P <- subset_samples(pdcy_phy, Group == "P")
pdcy_phy_P_gen <- collapseTaxLevel(pdcy_phy_P, "Genus")
pdcy_phy_P_fam <- collapseTaxLevel(pdcy_phy_P, "Family")

# Control-only
pdcy_phy_C <- subset_samples(pdcy_phy, Group == "C")
pdcy_phy_C_gen <- collapseTaxLevel(pdcy_phy_C, "Genus")
pdcy_phy_C_fam <- collapseTaxLevel(pdcy_phy_C, "Family")
```

3.1 Alpha diversity

Look for associations between the markers/SCFAs and measures of alpha diversity (within-sample diversity) for two different indices: Shannon and inverse Simpson (both measuring richness and evenness).

3.1.1 Full data (PD + C together)

Calculate alpha diversity index values for full data (subsampled to an even number of reads per sample), and plot the values to check for outliers (dashed line: 3rd quartile + 1.5*IQR).

```r
# Rarefy data for alpha diversity calculations
pdcy_phy_r <- rarefy_even_depth(pdcy_phy, rngseed = 2441662)

# Calculate alpha diversity values and add to metadata
pdcy_meta <- cbind(pdcy_meta,
                   estimate_richness(pdcy_phy_r, measures = c("Shannon", "InvSimpson")))

# Check what the values look like (to screen for potential outliers etc)
adiv_check <- lapply(c("Shannon", "InvSimpson"), function(x) ol_plot(pdcy_meta, x))
do.call("grid.arrange", c(adiv_check, nrow = 1))
```

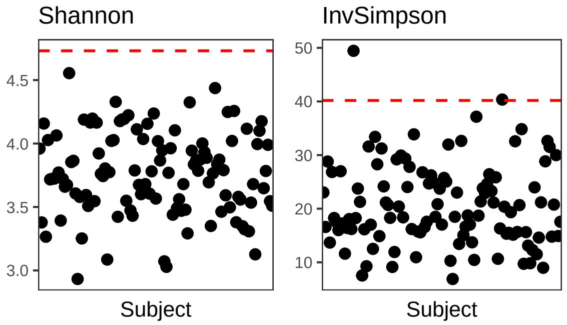

One subject had a clearly higher inverse Simpson value than everyone else; replace this subjects' alpha diversity estimates with NAs to exclude them from the comparisons.

```r
pdcy_meta[pdcy_meta$InvSimpson == max(pdcy_meta$InvSimpson),
          c("Shannon", "InvSimpson")] <- NA
```

Calculate correlations for alpha diversity vs markers/SCFAs and show results significant for at least one

diversity measure:

```
# Function for calculating correlations
divCors <- function(df, varlist){
  res <- t(sapply(varlist, function(x) c(
    unname(unlist(cor.test(y = df[,x], x = df$Shannon)[c("estimate", "p.value")])),
    unname(unlist(cor.test(y = df[,x], x = df$InvSimpson)[c("estimate", "p.value")])))))
  colnames(res) <- c("ShanCor", "ShanP", "InvSCor", "InvSP")
  res <- data.frame(Var = varlist, res)
  return(res)
}

# Calculate correlations
adiv_cors_full <- divCors(pdcy_meta, inf_scfa_vars)

# Significant results
kable_styling(kable(subset(adiv_cors_full, rowSums(adiv_cors_full[, c(3, 5)] < 0.05) > 0),
      booktabs = TRUE, linesep = "", digits = 4, row.names = FALSE), font_size = 8)
```

Var	ShanCor	ShanP	InvSCor	InvSP
total_SCFAs	-0.2091	0.0283	-0.1722	0.0720
propionic_acid	-0.2363	0.0129	-0.1694	0.0768
butyric_acid	-0.1950	0.0412	-0.1907	0.0459
SNGAL	-0.3643	0.0001	-0.2787	0.0040
Scalprotectin	-0.2780	0.0034	-0.2325	0.0150
Szonulin	-0.2935	0.0019	-0.2279	0.0167
SIL1b	-0.1890	0.0490	-0.1898	0.0481

3.1.2 Control only / PD only

Calculate similar correlations separately for control only and PD only subsets and show significant results:

```
# C-only correlations
adiv_cors_C <- data.frame(divCors(subset(pdcy_meta, Group == "C"), inf_scfa_vars),
                          Set = "C_only")
# PD-only correlations
adiv_cors_PD <- data.frame(divCors(subset(pdcy_meta, Group == "P"), inf_scfa_vars),
                           Set = "P_only")
# Combine
adiv_cors_sep <- rbind(adiv_cors_C, adiv_cors_PD)

# Order by variable
adiv_cors_sep <- adiv_cors_sep[order(adiv_cors_sep$Var), ]

# Variables with significant hits for either subset
adiv_cors_sep_sigs <- as.character(unique(
  adiv_cors_sep[which(rowSums(adiv_cors_sep[, c(3, 5)] < 0.05) > 0), "Var"]))

# Results for the variables that are significant for either subset
kable_styling(kable(subset(adiv_cors_sep, Var %in% adiv_cors_sep_sigs), booktabs = TRUE,
                    linesep = "", digits = 4, row.names = FALSE), font_size = 8)
```

Var	ShanCor	ShanP	InvSCor	InvSP	Set
acetic_acid	-0.3717	0.0052	-0.3233	0.0160	C_only
acetic_acid	-0.0175	0.8993	0.0001	0.9995	P_only
butyric_acid	-0.3165	0.0186	-0.2873	0.0334	C_only
butyric_acid	-0.1097	0.4254	-0.1203	0.3818	P_only
propionic_acid	-0.4445	0.0007	-0.3755	0.0047	C_only
propionic_acid	-0.0119	0.9313	0.0456	0.7410	P_only
Scalprotectin	-0.2191	0.1115	-0.2476	0.0711	C_only
Scalprotectin	-0.3075	0.0224	-0.2298	0.0914	P_only
SCXCL8	0.0427	0.7569	-0.0867	0.5291	C_only
SCXCL8	-0.2825	0.0366	-0.2453	0.0711	P_only
SNGAL	-0.4994	0.0001	-0.4213	0.0015	C_only
SNGAL	-0.2047	0.1496	-0.1497	0.2943	P_only
Szonulin	-0.3648	0.0062	-0.1952	0.1533	C_only
Szonulin	-0.2412	0.0760	-0.2473	0.0687	P_only
total_SCFAs	-0.4105	0.0019	-0.3513	0.0085	C_only
total_SCFAs	-0.0285	0.8364	-0.0210	0.8788	P_only

Fig 4

Make a figure summarizing the alpha diversity correlation results:

```
# Combine the results for full + subset
adiv_cors_full$Set <- "Full"
adiv_cors <- melt(rbind(adiv_cors_full, adiv_cors_sep))

# Split some variables
adiv_cors$Value <- sub(".*[nS]", "", adiv_cors$variable)
adiv_cors$Div <- sub("[PC].*", "", adiv_cors$variable)
adiv_cors$Set <- factor(adiv_cors$Set, levels = c("Full", "C_only", "P_only"))

# Reorganize data.frame
adiv_cors_df <- dcast(adiv_cors, Var + Set + Div ~ Value)

# Reorder variables for nicer plotting
adiv_cors_df$Var <- factor(adiv_cors_df$Var, levels = rev(inf_scfa_vars))
adiv_cors_df$Div <- factor(adiv_cors_df$Div, levels = c("Shan", "InvS"))
levels(adiv_cors_df$Div) <- c("Shannon", "Inverse Simpson")
levels(adiv_cors_df$Set) <- c("Full\ndata", "Controls\nonly", "PD\nonly")
levels(adiv_cors_df$Var) <- sub(" tool", " ", varLabelFix(levels(adiv_cors_df$Var)))

adiv_cors_plot <- ggplot(adiv_cors_df, aes(x = Set, y = Var, fill = Cor)) +
  geom_tile(color = "gray30") +
  theme_bw(base_size = 9) +
  facet_grid(~Div) +
  scale_fill_gradient2(low = "gray33", mid = "white", high = "firebrick",
                       name = "r", limits = c(-0.5, 0.5)) +
  geom_text(aes(label = stars.pval(P)), size = 4) +
  theme(legend.position = "bottom",
        axis.text = element_text(color = "black"),
        axis.title = element_blank(),
        panel.grid = element_blank())
adiv_cors_plot
```

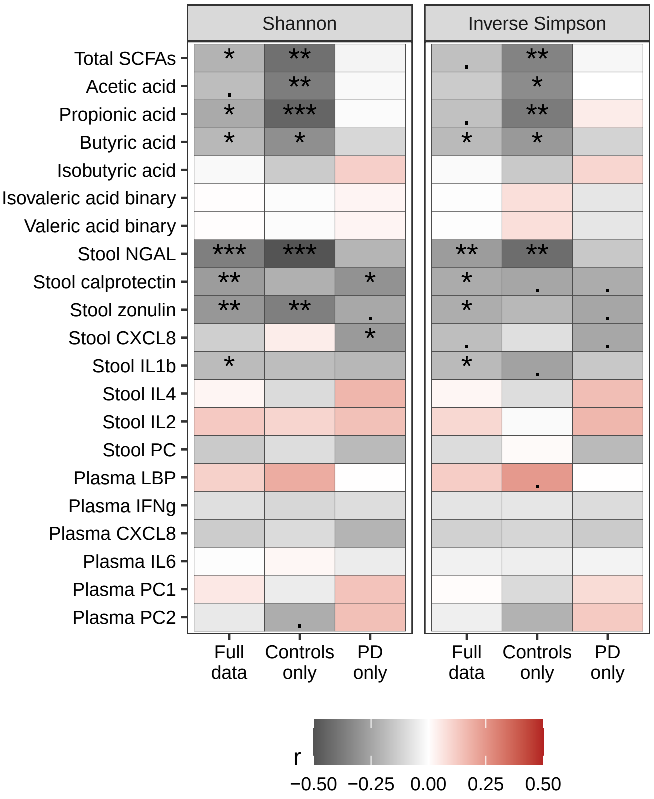


```
ggsave(adiv_cors_plot, filename = "Outputs/fig4_adiv_cors.pdf",
       width = maxwd/2, height = maxhi/2, units = "mm",
       device = cairo_pdf)
```

3.1.3 Corrected for PD vs C

To further explore the relationship of diversity, markers/SCFAs and PD, run linear regressions with the marker/SCFA as the dependent variable and diversity index + Group as explanatory variables. Test with a few example cases:

```
# Make model
lm_calpro_shan <- lm(Scalprotectin ~ Shannon + Group, pdcy_meta)

# Residual plots
autoplot(lm_calpro_shan, label.size = 2, nrow = 1) + theme_bw(base_size = 8)
```

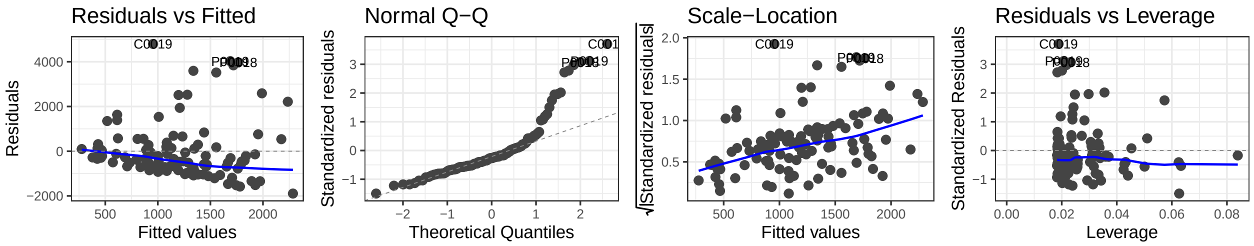

The diagnostic plots for the residuals imply heteroscedasticity. Log-transform calprotectin and plot again:

```
lm_shan_calpro_2 <- lm(log(Scalprotectin) ~ Shannon + Group, pdcy_meta)
autoplot(lm_shan_calpro_2, label.size = 2, nrow = 1) + theme_bw(base_size = 8)
```

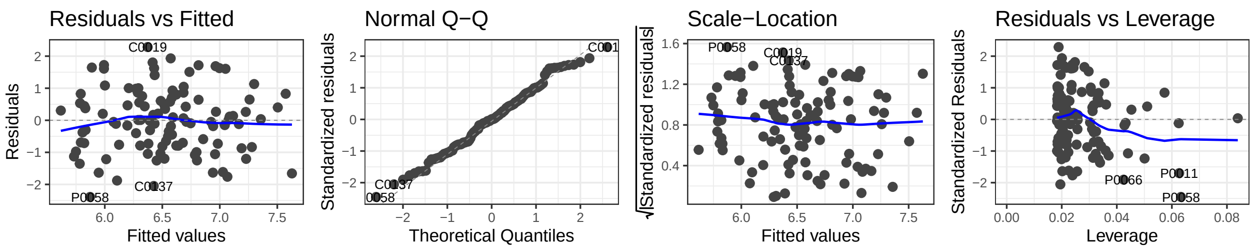

Run a similar test for Butyric acid ~ inverse Simpson + Group:

```
lm_buty_shan <- lm(butyric_acid ~ InvSimpson + Group, pdcy_meta)
autoplot(lm_buty_shan, label.size = 2, nrow = 1) + theme_bw(base_size = 8)
```

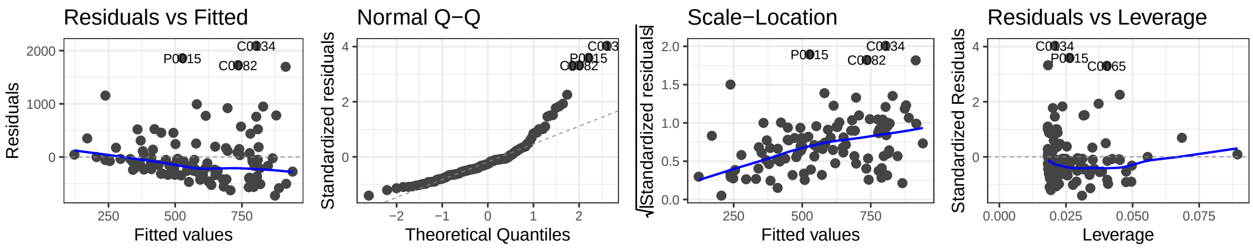

Try with log-transformed SCFA data:

```
lm_buty_shan2 <- lm(log(butyric_acid) ~ InvSimpson + Group, pdcy_meta)
autoplot(lm_buty_shan2, label.size = 2, nrow = 1) + theme_bw(base_size = 8)
```

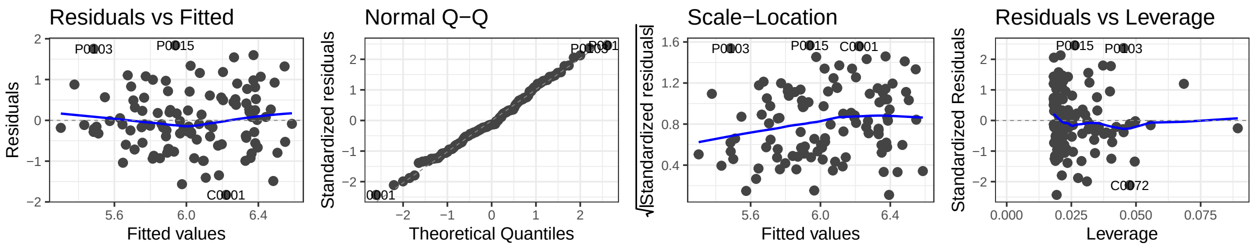

Since the log-transformed response variables seem to provide better results, run all regression testing with log-transformed response variables. Test both diversity measures for the markers that were significant for full data for at least one measure in the previous regressions. Include additional correction for sex, since it seemed to have a strong effect on the PD-differences for some SCFAs and markers.

```
lm_vars <- as.character(unique(subset(adiv_cors,
                  value < 0.05 & Value == "P" & Set == "Full")$Var))

pdc_div_lms <- as.data.frame(matrix(ncol = 4, nrow = 0))
colnames(pdc_div_lms) <- c("Model", "pValModel", "pValGroup", "pValDiv")

for(i in lm_vars){
  for(j in c("Shannon", "InvSimpson")){
    m <- as.formula(paste("log(", i, ")~sex+Group+", j))
    res <- summary(lm(m, pdcy_meta))
    pdc_div_lms <- rbind(pdc_div_lms,
                     data.frame(Model = deparse(m),
                                pValModel = unname(pf(res$fstatistic[1],
                                                      res$fstatistic[2],
                                                      res$fstatistic[3],
                                                      lower.tail=FALSE)),
```

```r
                                        pValSex = res$coefficients[2, 4],
                                        pValGroup = res$coefficients[3, 4],
                                        pValDiv = res$coefficients[4, 4]))
  }
}

# Show results
kable_styling(kable(pdc_div_lms, digits = 4, booktabs = TRUE, linesep = "",
                    row.names = FALSE), font_size = 8)
```

Model	pValModel	pValSex	pValGroup	pValDiv
log(total_SCFAs) ~ sex + Group + Shannon	0.0101	0.3113	0.0250	0.0089
log(total_SCFAs) ~ sex + Group + InvSimpson	0.0362	0.3486	0.0414	0.0434
log(propionic_acid) ~ sex + Group + Shannon	0.0057	0.5977	0.0196	0.0039
log(propionic_acid) ~ sex + Group + InvSimpson	0.0452	0.6625	0.0365	0.0501
log(butyric_acid) ~ sex + Group + Shannon	0.0002	0.2563	0.0002	0.0058
log(butyric_acid) ~ sex + Group + InvSimpson	0.0006	0.2855	0.0005	0.0184
log(SNGAL) ~ sex + Group + Shannon	0.0000	0.9947	0.3654	0.0000
log(SNGAL) ~ sex + Group + InvSimpson	0.0017	0.8792	0.2559	0.0002
log(Scalprotectin) ~ sex + Group + Shannon	0.0000	0.0208	0.0620	0.0000
log(Scalprotectin) ~ sex + Group + InvSimpson	0.0000	0.0232	0.0327	0.0000
log(Szonulin) ~ sex + Group + Shannon	0.0104	0.8834	0.8230	0.0010
log(Szonulin) ~ sex + Group + InvSimpson	0.0462	0.8295	0.6500	0.0059
log(SIL1b) ~ sex + Group + Shannon	0.4071	0.1299	0.4814	0.5642
log(SIL1b) ~ sex + Group + InvSimpson	0.2584	0.1251	0.5000	0.2287

Additional file 6A

```r
# Fix variable labels and reorganize
pdc_div_lms$Model <- sub("\\(S", "\\(Stool ", sub("_acid", " acid", pdc_div_lms$Model))
pdc_div_lms$Variable <- capitalize(sub("\\).*", "", sub("log\\(", "", pdc_div_lms$Model)))
pdc_div_lms$DivIndex <- sub(".*\\+ ", "", pdc_div_lms$Model)
pdc_div_lms <- pdc_div_lms[, c("Model", "Variable", "DivIndex", "pValModel",
                               "pValSex", "pValGroup", "pValDiv")]

# Export
write.csv(pdc_div_lms, "Outputs/add5A_div_lms.csv", row.names = FALSE)
```

Additionally, test a set of models with an interaction: log(marker or SCFA) ~ sex + Group * Diversity.

```r
pdc_div_lms2 <- as.data.frame(matrix(ncol = 5, nrow = 0))
colnames(pdc_div_lms2) <- c("Model", "pValModel",
                            "pValGroup", "pValDiv", "pValGroupDiv")

for(i in lm_vars){
  for(j in c("Shannon", "InvSimpson")){
    m <- as.formula(paste("log(", i, ")~sex + Group *", j))
    res <- summary(lm(m, pdcy_meta))
    pdc_div_lms2 <- rbind(pdc_div_lms2,
                          data.frame(Model = deparse(m),
                                     pValModel = unname(pf(res$fstatistic[1],
                                                           res$fstatistic[2],
                                                           res$fstatistic[3],
                                                           lower.tail = FALSE)),
                                     pValSex = res$coefficients[2, 4],
                                     pValGroup = res$coefficients[3, 4],
```

```
                                         pValDiv = res$coefficients[4, 4],
                                         pValGroupDiv = res$coefficients[5, 4]))
  }
}

# Trim to those significant models where group and interaction are p < 0.1
pdc_div_sig_lms2 <- subset(pdc_div_lms2, pValModel < 0.05 &
                              pValGroup < 0.1 & pValGroupDiv < 0.1)

# Show results
kable_styling(kable(pdc_div_sig_lms2, digits = 4, booktabs = TRUE,
                    linesep = "", row.names = FALSE), font_size = 8)
```

Model	pValModel	pValSex	pValGroup	pValDiv	pValGroupDiv
log(total_SCFAs) ~ sex + Group * Shannon	0.0041	0.3462	0.0291	0.0012	0.0454
log(total_SCFAs) ~ sex + Group * InvSimpson	0.0161	0.3906	0.0137	0.0060	0.0567
log(propionic_acid) ~ sex + Group * Shannon	0.0012	0.6603	0.0117	0.0002	0.0197
log(propionic_acid) ~ sex + Group * InvSimpson	0.0079	0.7396	0.0033	0.0020	0.0166
log(butyric_acid) ~ sex + Group * Shannon	0.0002	0.2829	0.0473	0.0018	0.0941
log(SNGAL) ~ sex + Group * Shannon	0.0000	0.9085	0.0925	0.0000	0.0781

Additional file 6B

```
# Fix variable labels
pdc_div_lms2$Model <- sub("\\(S", "\\(Stool ", sub("_acid", " acid", pdc_div_lms2$Model))
pdc_div_lms2$Variable <- capitalize(
  sub("\\).*", "", sub("log\\(", "", pdc_div_lms2$Model)))
pdc_div_lms2$DivIndex <- sub(".*\\* ", "", pdc_div_lms2$Model)
pdc_div_lms2 <- pdc_div_lms2[, c("Model", "Variable", "DivIndex", "pValModel",
                                 "pValSex", "pValGroup", "pValDiv", "pValGroupDiv")]

# Export
write.csv(pdc_div_lms2, "Outputs/add5B_div_lms.csv", row.names = FALSE)
```

3.1.4 Analyses with additional confounders

Finally, explore a few of the variable of interest ~ PD/control + diversity models in more detail:

Calprotectin

Previous results for calprotectin models:

```
pdc_div_lms$pValGroupDiv <- NA
pdc_div_lm_all <- rbind(pdc_div_lms, pdc_div_lms2)

kable_styling(kable(pdc_div_lm_all[grep("calpro", pdc_div_lm_all$Model), c(1, 4:8)],
      digits = 4, booktabs = TRUE, linesep = "", row.names = FALSE), font_size = 8)
```

Model	pValModel	pValSex	pValGroup	pValDiv	pValGroupDiv
log(Stool calprotectin) ~ sex + Group + Shannon	0	0.0208	0.0620	0.0000	NA
log(Stool calprotectin) ~ sex + Group + InvSimpson	0	0.0232	0.0327	0.0000	NA
log(Stool calprotectin) ~ sex + Group * Shannon	0	0.0196	0.3708	0.0133	0.4600
log(Stool calprotectin) ~ sex + Group * InvSimpson	0	0.0240	0.4905	0.0068	0.9875

Results were similar for the two indices; focus on inverse Simpson, the index with the lower p-value. The

interaction was not significant for PD * diversity. As alternatives, since there was a notable sex difference for calprotectin, test additional model with interaction:

```r
print(anova(lm(log(Scalprotectin) ~ Group + sex + InvSimpson, data = pdcy_meta),
            lm(log(Scalprotectin) ~ Group + sex * InvSimpson, data = pdcy_meta)))
```

```
## Analysis of Variance Table
##
## Model 1: log(Scalprotectin) ~ Group + sex + InvSimpson
## Model 2: log(Scalprotectin) ~ Group + sex * InvSimpson
##   Res.Df     RSS Df Sum of Sq      F    Pr(>F)
## 1    105 102.249
## 2    104  90.482  1    11.767 13.525 0.0003747 ***
## ---
## Signif. codes:  0 '***' 0.001 '**' 0.01 '*' 0.05 '.' 0.1 ' ' 1
```

In fact, it seems there could be an interaction for sex * diversity; continue with that model. Potential additional confounders with $p < 0.05$ for calprotectin based on the earlier clinical variable correlations:

```r
calp_conf <- subset(meta_cors_st_P, variable == "Scalprotectin" &
                      value == TRUE & Set == "All subjects")$Confounder
calp_conf
```

```
## [1] BPS consistency  BPS frequency    History diabetes PD relative
## [5] RBDSQ
## 67 Levels: Age at stool collection BMI BPS consistency ... UPDRS IV total
```

The two BPS variables (stool consistency vs frequency) are somewhat correlated ($r = 0.198$, but both are included nevertheless. Test a model with all the potential confounders plus sex, then repeat with only significant confounders plus Group and inverse Simpson, and collect results into a table:

```r
# Original model without any confounders
calp_m0 <- lm(log(Scalprotectin) ~ Group + sex * InvSimpson, pdcy_meta)

# All confounders
calp_m1 <- lm(log(Scalprotectin) ~ Group + sex * InvSimpson +
                history_diabetes + PD_relative + RBDSQ +
                BPS_characteristic_average +
                BPS_frequency_average, pdcy_meta)
# Check results
summary(calp_m1)
```

```
##
## Call:
## lm(formula = log(Scalprotectin) ~ Group + sex * InvSimpson +
##     history_diabetes + PD_relative + RBDSQ + BPS_characteristic_average +
##     BPS_frequency_average, data = pdcy_meta)
##
## Residuals:
##      Min       1Q   Median       3Q      Max
## -1.88917 -0.58893 -0.05144  0.66258  1.97559
##
## Coefficients:
##                             Estimate Std. Error t value Pr(>|t|)
## (Intercept)                 7.686464   0.360139  21.343  < 2e-16 ***
## GroupP                      0.006561   0.213621   0.031   0.9756
## sexM                       -1.171073   0.519552  -2.254   0.0264 *
```

```
## InvSimpson                  -0.084328   0.015744  -5.356 5.53e-07 ***
## history_diabetes1            0.560717   0.274450   2.043   0.0437 *
## PD_relative1                 0.519203   0.239135   2.171   0.0323 *
## RBDSQ                        0.050646   0.036703   1.380   0.1707
## BPS_characteristic_average -0.160593   0.077869  -2.062   0.0418 *
## BPS_frequency_average      -0.137761   0.103753  -1.328   0.1873
## sexM:InvSimpson              0.078450   0.024459   3.207   0.0018 **
## ---
## Signif. codes:  0 '***' 0.001 '**' 0.01 '*' 0.05 '.' 0.1 ' ' 1
##
## Residual standard error: 0.8756 on 99 degrees of freedom
##   (2 observations deleted due to missingness)
## Multiple R-squared:  0.4183, Adjusted R-squared:  0.3655
## F-statistic: 7.911 on 9 and 99 DF,  p-value: 1.004e-08
```

```
# History of diabetes, BPS characteristic and PD-relative are significant,
# keep them:
calp_m2 <- lm(log(Scalprotectin) ~ Group + sex * InvSimpson +
                BPS_characteristic_average +
                history_diabetes + PD_relative, pdcy_meta)

# Package for convenient table output
library("huxtable")

# Customized table function
custom_huxreg <- function(m0, m1, m2, cn){
  ht <- huxreg("Simple model" = m0,
               "All confounders" = m1,
               "Main confounders" = m2,
               coefs = cn, error_pos = "same",
               stars = c(`·` = 0.1, `*` = 0.05, `**` = 0.01, `***` = 0.001))
  position(ht)  <- "left"
  ht <- theme_plain(ht)
  return(ht)
}

calp_coefs <- c('Sex' = "sexM",
                'PD vs control' = 'GroupP',
                'Inverse Simpson index' = 'InvSimpson',
                'Diabetes' = 'history_diabetes1',
                'Relative with PD' = 'PD_relative1',
                'RBDSQ score' = 'RBDSQ',
                'BPS stool consistency' = 'BPS_characteristic_average',
                'BPS defecation frequency' = 'BPS_frequency_average',
                'sex and inverse Simpson interaction ' = 'sexM:InvSimpson')

# Show results of each model side by side
custom_huxreg(calp_m0, calp_m1, calp_m2, calp_coefs)
```

```
# Export
quick_xlsx(custom_huxreg(calp_m0, calp_m1, calp_m2, calp_coefs),
           file = "Outputs/add5C_calp_div_lms.xlsx", open = FALSE)
```

Propionic acid

Previous results for propionic acid models:

	Simple model	All confounders	Main confounders
Sex	-1.461 ** (0.546)	-1.171 * (0.520)	-1.316 * (0.521)
PD vs control	0.381 * (0.179)	0.007 (0.214)	0.211 (0.177)
Inverse Simpson index	-0.095 *** (0.016)	-0.084 *** (0.016)	-0.091 *** (0.015)
Diabetes		0.561 * (0.274)	0.596 * (0.277)
Relative with PD		0.519 * (0.239)	0.461 · (0.238)
RBDSQ score		0.051 (0.037)	
BPS stool consistency		-0.161 * (0.078)	-0.181 * (0.077)
BPS defecation frequency		-0.138 (0.104)	
sex and inverse Simpson interaction	0.093 *** (0.025)	0.078 ** (0.024)	0.087 *** (0.024)
N	109	109	109
R2	0.307	0.418	0.393
logLik	-144.517	-134.940	-137.258
AIC	301.033	291.880	292.517

*** p < 0.001; ** p < 0.01; * p < 0.05; · p < 0.1.

```
kable_styling(kable(pdc_div_lm_all[grep("propionic", pdc_div_lm_all$Model), c(1, 4:8)],
      digits = 4, booktabs = TRUE, linesep = "", row.names = FALSE), font_size = 8)
```

Model	pValModel	pValSex	pValGroup	pValDiv	pValGroupDiv
log(propionic acid) ~ sex + Group + Shannon	0.0057	0.5977	0.0196	0.0039	NA
log(propionic acid) ~ sex + Group + InvSimpson	0.0452	0.6625	0.0365	0.0501	NA
log(propionic acid) ~ sex + Group * Shannon	0.0012	0.6603	0.0117	0.0002	0.0197
log(propionic acid) ~ sex + Group * InvSimpson	0.0079	0.7396	0.0033	0.0020	0.0166

Focus on Shannon, which had the lower p-values. Which is better, model with an interaction or without?

```
print(anova(
  lm(log(propionic_acid) ~ sex + Group + Shannon, data = pdcy_meta),
  lm(log(propionic_acid) ~ sex + Group * Shannon, data = pdcy_meta)))
```

```
## Analysis of Variance Table
##
## Model 1: log(propionic_acid) ~ sex + Group + Shannon
## Model 2: log(propionic_acid) ~ sex + Group * Shannon
##   Res.Df    RSS Df Sum of Sq      F  Pr(>F)
## 1    106 35.901
## 2    105 34.080  1    1.8212 5.6112 0.01967 *
## ---
## Signif. codes:  0 '***' 0.001 '**' 0.01 '*' 0.05 '.' 0.1 ' ' 1
```

The model with an interaction was better, so continue with that, and add correction for confounding variables. Potential confounders with $p < 0.05$ for propionic acid from the clinical variable correlations:

```
prop_conf <- subset(meta_cors_sc_P, variable == "propionic_acid" &
                      value == TRUE & Set == "All subjects")$Confounder
prop_conf
```

```
## [1] GDS15                  Medication ACE AT1     Medication statin
## [4] NMSS total             Rome III 9-15 sum score
## 67 Levels: Age at stool collection BMI BPS consistency ... UPDRS IV total
```

Test a model with all potential confounders plus sex, then repeat with significant confounders plus Group and inverse Simpson:

```
# Original model
prop_m0 <- lm(log(propionic_acid) ~ sex + Group * Shannon, pdcy_meta)

# All confounders
prop_m1 <- lm(log(propionic_acid) ~ sex + Group * Shannon + GDS15 + meds_ACE_AT1 +
                meds_statin + NMSS_total + Rome_III_constip_defec_sumscore_9.15,
              pdcy_meta)
# Check results
summary(prop_m1)
```

```
##
## Call:
## lm(formula = log(propionic_acid) ~ sex + Group * Shannon + GDS15 +
##     meds_ACE_AT1 + meds_statin + NMSS_total + Rome_III_constip_defec_sumscore_9.15,
##     data = pdcy_meta)
##
## Residuals:
##      Min       1Q   Median       3Q      Max
## -1.39621 -0.33566  0.03743  0.30615  1.34008
##
## Coefficients:
##                                       Estimate Std. Error t value Pr(>|t|)
## (Intercept)                           9.727701   0.911298  10.675  < 2e-16 ***
## sexM                                 -0.025213   0.111227  -0.227 0.821136
## GroupP                               -2.792932   1.299102  -2.150 0.033975 *
## Shannon                              -0.854215   0.238488  -3.582 0.000529 ***
## GDS15                                -0.029178   0.022658  -1.288 0.200804
## meds_ACE_AT11                         0.165356   0.116188   1.423 0.157798
## meds_statin1                          0.163215   0.127530   1.280 0.203573
## NMSS_total                            0.002473   0.002634   0.939 0.350221
## Rome_III_constip_defec_sumscore_9.15 -0.030991   0.013985  -2.216 0.028960 *
## GroupP:Shannon                        0.736296   0.347652   2.118 0.036662 *
## ---
## Signif. codes:  0 '***' 0.001 '**' 0.01 '*' 0.05 '.' 0.1 ' ' 1
##
## Residual standard error: 0.5551 on 100 degrees of freedom
##   (1 observation deleted due to missingness)
## Multiple R-squared:  0.2372, Adjusted R-squared:  0.1685
## F-statistic: 3.454 on 9 and 100 DF,  p-value: 0.0009582
```

```
# Significant confounders
prop_m2 <- lm(log(propionic_acid) ~ Group * Shannon +
```

```
                Rome_III_constip_defec_sumscore_9.15, pdcy_meta)

prop_coefs <- c('Sex' = "sexM",
                'PD vs control' = 'GroupP',
                'Shannon index' = 'Shannon',
                'GDS15 score' = 'GDS15',
                'ACE-AT1 medication' = 'meds_ACE_AT11',
                'Statin medication' = 'meds_statin1',
                'NMSS total score' = 'NMSS_total',
                'Rome III 9-15 sum score' =
                   'Rome_III_constip_defec_sumscore_9.15',
                'PD/C and Shannon interaction' = 'GroupP:Shannon')

# Show results
custom_huxreg(prop_m0, prop_m1, prop_m2, prop_coefs)
```

	Simple model	All confounders	Main confounders
Sex	0.048 (0.109)	-0.025 (0.111)	
PD vs control	-3.313 * (1.292)	-2.793 * (1.299)	-3.332 ** (1.266)
Shannon index	-0.925 *** (0.243)	-0.854 *** (0.238)	-0.898 *** (0.239)
GDS15 score		-0.029 (0.023)	
ACE-AT1 medication		0.165 (0.116)	
Statin medication		0.163 (0.128)	
NMSS total score		0.002 (0.003)	
Rome III 9-15 sum score		-0.031 * (0.014)	-0.026 * (0.013)
PD/C and Shannon interaction	0.812 * (0.343)	0.736 * (0.348)	0.853 * (0.336)
N	110	110	110
R2	0.156	0.237	0.188
logLik	-91.635	-86.098	-89.501
AIC	195.271	194.196	191.003
*** p < 0.001; ** p < 0.01; * p < 0.05; · p < 0.1.			

```
# Export
quick_xlsx(custom_huxreg(prop_m0, prop_m1, prop_m2, prop_coefs),
           file = "Outputs/add5D_prop_div_lms.xlsx", open = FALSE)
```

Fig 5

Plots for the SCFA/markers ~ diversity linear regressions:

```
# Custom scatterplot function
custom_lm_pd_s <- function(df, xvar, yvar){
  ggplot(df, aes_string(x = xvar, y = yvar,
```

```r
                        color = "Group", shape = "Group", fill = "Group")) +
    geom_point(size = 1.5) +
    facet_grid(~sex,
               labeller = labeller(sex = setNames(c("Female", "Male"), c("F", "M")))) +
    scale_color_manual(values = c("gray50", "black"), label = pdc_labels) +
    scale_shape_manual(values = c(16, 17), label = pdc_labels) +
    scale_fill_manual(values = c("gray75", "gray25"), label = pdc_labels) +
    theme_bw(base_size = 10) +
    geom_smooth(method = "lm", size = 0.75, alpha = 0.3) +
    theme(panel.grid = element_blank(),
          axis.text = element_text(color = "black"),
          legend.position = "bottom",
          legend.key.height = unit(4, "mm"),
          legend.title = element_blank(),
          plot.title = element_text(face = "bold"),
          strip.background = element_rect(fill = "white"),
          legend.box.background = element_rect(color = "black"))
}

# Put together figure
pdc_lm_plots <- arrangeGrob(
  custom_lm_pd_s(pdcy_meta, "InvSimpson", "log(Scalprotectin)") +
    ylab("log(Stool calprotectin)") +
    xlab("Inverse Simpson index") +
    ggtitle("A"),
  custom_lm_pd_s(pdcy_meta, "Shannon", "log(propionic_acid)") +
    ylab("log(propionic acid)") +
    xlab("Shannon index") +
    ggtitle("B"),
  ncol = 1)
grid.arrange(pdc_lm_plots)
```

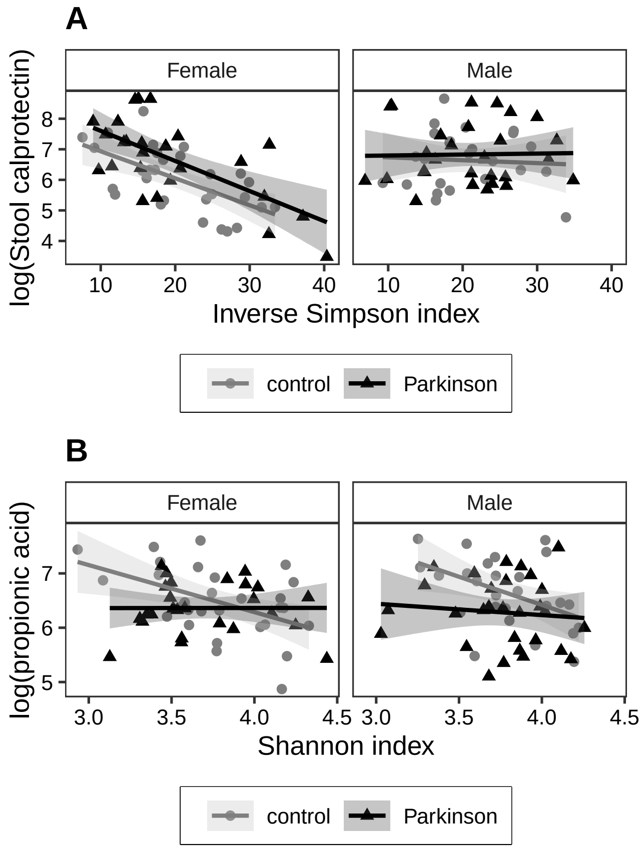


```
# Export
ggsave(pdc_lm_plots, filename = "Outputs/fig5_adiv_lms.pdf",
       width = maxwd/2, height = 125, units = "mm",
       device = cairo_pdf)
```

3.2 Beta diversity

Test beta diversity differences based on Bray-Curtis dissimilarities of subsampled data with adonis (PERMANOVA). This test does not accept missing values, so drop subjects with NAs from the comparisons for each variable.

3.2.1 Full data (PD + C together)

```
# Run adonis for each variable separately
bdiv_all <- list()

for(i in inf_scfa_vars){
  phy <- subset_samples(pdcy_phy, !is.na(sample_data(pdcy_phy)[[i]]))
  rar <- rarefy_even_depth(phy, rngseed = 1942890)
  dis <- vegdist(as.data.frame(t(as.matrix(otu_table(rar)))))
  bdiv_all[[i]] <- adonis(as.formula(paste("dis ~", i)),
                          data = as(sample_data(rar), "data.frame"),
                          permutations = 9999)$aov.tab
}

# Grab p-values
bdiv_all_p <- sapply(inf_scfa_vars, function(x) bdiv_all[[x]][["Pr(>F)"]][1])
```

```r
# Make data frame
bdiv_all_p <- data.frame(Variable = names(bdiv_all_p),
                         pVar = bdiv_all_p)
```

Significant results for these comparisons:

```r
kable(subset(bdiv_all_p, pVar < 0.05), row.names = FALSE, booktabs = TRUE,
      linesep = "", digits = 3)
```

Variable	pVar
total_SCFAs	0.000
acetic_acid	0.000
propionic_acid	0.000
butyric_acid	0.000
SNGAL	0.008
Szonulin	0.001
SIL2	0.030
StoolPC	0.016

3.2.2 Corrected for PD vs control

Run adonis2 models with the immune marker/SCFA variables corrected for Group, since based on previous analyses, there is a significant beta diversity difference between PD and control.

```r
# Run adonis for each variable
bdiv_pdc <- list()

for(i in inf_scfa_vars){
  phy <- subset_samples(pdcy_phy, !is.na(sample_data(pdcy_phy)[[i]]))
  rar <- rarefy_even_depth(phy, rngseed = 1942890)
  dis <- vegdist(as.data.frame(t(as.matrix(otu_table(rar)))))
  bdiv_pdc[[i]] <- adonis2(as.formula(paste("dis ~ Group + ", i)),
                           data = as(sample_data(rar), "data.frame"),
                           permutations = 9999, by = "margin")["Pr(>F)"]
}

# Grab p-values into data frame
bdiv_pdc_p <- data.frame(
  Var = inf_scfa_vars,
  pGroup = sapply(inf_scfa_vars, function(x) bdiv_pdc[[x]][1,]),
  pVar = sapply(inf_scfa_vars, function(x) bdiv_pdc[[x]][2,]))
```

Variables that were significant when corrected for Group:

```r
kable(subset(bdiv_pdc_p, pVar < 0.05), row.names = FALSE, booktabs = TRUE,
      linesep = "", digits = 3)
```

Var	pGroup	pVar
total_SCFAs	0.001	0.000
acetic_acid	0.000	0.000
propionic_acid	0.001	0.000
butyric_acid	0.002	0.000
SNGAL	0.001	0.007
Szonulin	0.000	0.000
SIL2	0.000	0.030
StoolPC	0.000	0.021

3.2.3 Control only / PD only

As an alternative PD/control comparison, run tests separately for PD-only and control-only subsets.

C-only

```
bdiv_c <- list()

for(i in inf_scfa_vars){
  phy <- subset_samples(pdcy_phy_C, !is.na(sample_data(pdcy_phy_C)[[i]]))
  rar <- rarefy_even_depth(phy, rngseed = 3656154)
  dis <- vegdist(as.data.frame(t(as.matrix(otu_table(rar)))))
  bdiv_c[[i]] <- adonis(as.formula(paste("dis ~", i)),
                        data = as(sample_data(rar), "data.frame"),
                        permutations = 9999)$aov.tab
}

# Grab p-values
bdiv_c_p <- sapply(inf_scfa_vars, function(x) bdiv_c[[x]][["Pr(>F)"]][1])

# Make data frame
bdiv_c_p <- data.frame(Var = names(bdiv_c_p),
                       pVar = bdiv_c_p)
```

PD-only

```
bdiv_pd <- list()

for(i in inf_scfa_vars){
  phy <- subset_samples(pdcy_phy_P, !is.na(sample_data(pdcy_phy_P)[[i]]))
  rar <- rarefy_even_depth(phy, rngseed = 2160957)
  dis <- vegdist(as.data.frame(t(as.matrix(otu_table(rar)))))
  bdiv_pd[[i]] <- adonis(as.formula(paste("dis ~", i)),
                         data = as(sample_data(rar), "data.frame"),
                         permutations = 9999)$aov.tab
}

# Grab p-values
bdiv_pd_p <- sapply(inf_scfa_vars, function(x) bdiv_pd[[x]][["Pr(>F)"]][1])

# Make data frame
bdiv_pd_p <- data.frame(Var = names(bdiv_pd_p),
                        pVar = bdiv_pd_p)
```

Results with a significant uncorrected p-value in either the C-only or PD-only comparisons:

```r
bdiv_pdc_separated <- data.frame(Var = rownames(bdiv_c_p),
                                 pVar_C = bdiv_c_p[,2],
                                 pVar_P = bdiv_pd_p[, 2])

kable(bdiv_pdc_separated[rowSums(bdiv_pdc_separated[, 2:3] < 0.05) > 0,],
      booktabs = TRUE, linesep = "", digits = 3, row.names = FALSE)
```

Var	pVar_C	pVar_P
total_SCFAs	0.000	0.066
acetic_acid	0.000	0.275
propionic_acid	0.001	0.217
butyric_acid	0.000	0.004
SNGAL	0.004	0.307
Szonulin	0.008	0.021

3.2.4 Analyses with additional confounders

Run the tests with additional correction for sex and the main beta diversity confounders from the follow-up microbiome article (Aho et al. 2019).

```r
# Run adonis for each variable
bdiv_pdc_conf <- list()

for(i in inf_scfa_vars){
  phy <- subset_samples(pdcy_phy, !is.na(sample_data(pdcy_phy)[[i]]))
  rar <- rarefy_even_depth(phy, rngseed = 1942890)
  dis <- vegdist(as.data.frame(t(as.matrix(otu_table(rar)))))
  bdiv_pdc_conf[[i]] <- adonis2(as.formula(
  paste("dis ~ Group + sex + Rome_III_constip_defec_sumscore_9.15 + BMI +", i)),
                             data = as(sample_data(rar), "data.frame"),
                             permutations = 9999, by = "margin")["Pr(>F)"]
}

# Grab p-values into data frame
bdiv_pdc_conf_p <- data.frame(
  Var = inf_scfa_vars,
  pGroup = sapply(inf_scfa_vars, function(x) bdiv_pdc_conf[[x]][1,]),
  pVar = sapply(inf_scfa_vars, function(x) bdiv_pdc_conf[[x]][5,]))
```

Significant results:

```r
kable(subset(bdiv_pdc_conf_p, pVar < 0.05),
     row.names = FALSE, booktabs = TRUE, linesep = "", digits = 3)
```

Var	pGroup	pVar
total_SCFAs	0.009	0.000
acetic_acid	0.008	0.001
propionic_acid	0.007	0.000
butyric_acid	0.016	0.000
SNGAL	0.031	0.005
Szonulin	0.008	0.002
SIL2	0.006	0.030
StoolPC	0.008	0.023

Additional file 7A

Collect all the results from the above comparisons into one data frame and export:

```
bdiv_combo <- data.frame(
  Variable = bdiv_all_p$Variable,
  'p (full data)' = bdiv_all_p$pVar,
  'p (control only)' = bdiv_pdc_separated$pVar_C,
  'p (PD only)' = bdiv_pdc_separated$pVar_P,
  'p (PD/C corrected)' = bdiv_pdc_p$pVar,
  'p (PD/C + confounder corrected)' = bdiv_pdc_conf_p$pVar,
  check.names = FALSE
)

# Variables with anything significant?
kable_styling(kable(bdiv_combo[rowSums(bdiv_combo[, 2:5] < 0.05) > 0,], booktabs = TRUE,
                    linesep = "", digits = 3, row.names = FALSE), font_size = 8)
```

Variable	p (full data)	p (control only)	p (PD only)	p (PD/C corrected)	p (PD/C + confounder corrected)
total_SCFAs	0.000	0.000	0.066	0.000	0.000
acetic_acid	0.000	0.000	0.275	0.000	0.001
propionic_acid	0.000	0.001	0.217	0.000	0.000
butyric_acid	0.000	0.000	0.004	0.000	0.000
SNGAL	0.008	0.004	0.307	0.007	0.005
Szonulin	0.001	0.008	0.021	0.000	0.002
SIL2	0.030	0.479	0.076	0.030	0.030
StoolPC	0.016	0.598	0.054	0.021	0.023

```
# Fix variable labels
bdiv_combo$Variable <- sub(" tool", " ", varLabelFix(bdiv_combo$Variable))

# Export the full combined table
write.csv(bdiv_combo, "Outputs/add6A_bdivs.csv", row.names = FALSE)
```

Additional file 8

Plot the most interesting cases with variable values split into two categories by median for better visualization:

```
# Subsample to the same number of reads
pdcy_phy_R <- rarefy_even_depth(pdcy_phy, rngseed = 1942890)

# Calculate ordination
mds_all <- metaMDS(as.data.frame(t(as.matrix(otu_table(pdcy_phy_R)))),
                   try = 500, trace = FALSE)
# Get points
mds_all_df <- data.frame(NMDS1 = mds_all$points[,1],
                         NMDS2 = mds_all$points[,2],
                         as(sample_data(pdcy_phy_R), "data.frame"))

# Custom plot function
bdiv_plot_2cat <- function(df, var, u){
  var_bin <- factor(df[[var]] > median(df[[var]], na.rm = TRUE))
  levels(var_bin) <- substitute(c(
    paste(var, '\u2264', round(median(df[[var]], na.rm = TRUE), digits = 1), u),
    paste(var, ">", round(median(df[[var]], na.rm = TRUE), digits = 1), u)))
  df_temp <- data.frame(df, var_bin = var_bin)

  p <- ggplot(subset(df_temp, !is.na(var_bin)),
```

```r
              aes(x = NMDS1, y = NMDS2,
                  color = var_bin, linetype = var_bin, shape = var_bin)) +
    geom_point(size = 0.8) +
    theme_bw(base_size = 7) +
    coord_fixed() +
    facet_grid(~Group) +
    ggtitle(var) +
    stat_ellipse(level = 0.95, lwd = 0.5, aes(color = var_bin)) +
    scale_color_manual(values = c("gray60", "forestgreen"), name = NULL) +
    scale_linetype_manual(values = c(2, 1), name = NULL) +
    scale_shape_manual(values = c(16, 17), name = NULL) +
    guides(color = guide_legend(nrow = 2),
           linetype = guide_legend(nrow = 2),
           shape = guide_legend(nrow = 2)) +
    theme(panel.grid = element_blank(),
          legend.position = "bottom",
          legend.margin = margin(t = -2, b = -3, unit = "mm"),
          legend.key.height = unit(2, "mm"),
          plot.margin = unit(c(-6, 1, -5, 1), "mm"),
          plot.title = element_text(size = 8))
  rm(df_temp)
  return(p)
}

# Draw plots
bdiv_plots <- arrangeGrob(
  bdiv_plot_2cat(mds_all_df, "acetic_acid",
                 c('\u00B5mol/l')) + ggtitle("Acetic acid"),
  bdiv_plot_2cat(mds_all_df, "butyric_acid",
                  c('\u00B5mol/l')) + ggtitle("Butyric acid"),
  bdiv_plot_2cat(mds_all_df, "total_SCFAs",
                  c('\u00B5mol/l')) + ggtitle("Total SCFAs"),
  bdiv_plot_2cat(mds_all_df, "SNGAL",
                 "pg/mg total protein") + ggtitle("Stool NGAL"),
  bdiv_plot_2cat(mds_all_df, "SIL2",
                 "pg/mg total protein") + ggtitle("Stool IL2"),
  bdiv_plot_2cat(mds_all_df, "Szonulin",
                 "pg/mg total protein") + ggtitle("Stool zonulin"),
  ncol = 2)
grid.arrange(bdiv_plots)
```

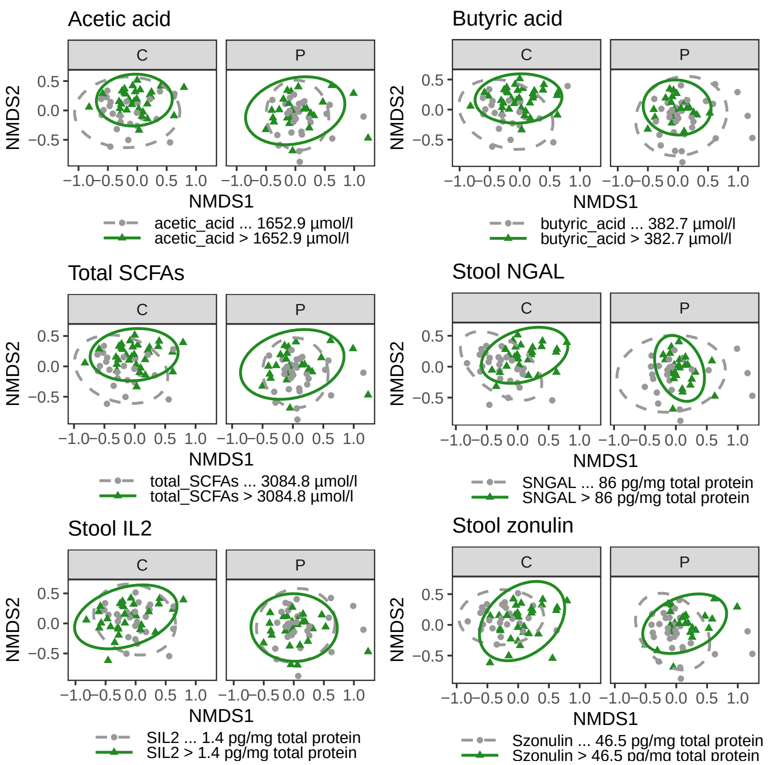


```
ggsave(bdiv_plots, filename = "Outputs/add7_bdiv_plots.pdf",
       device = cairo_pdf, width = 100, height = 120, units = "mm")
```

3.2.5 Interaction effects

Test for interactions between Group and markers/SCFAs:

```
bdiv_pdc_i <- list()

for(i in inf_scfa_vars){
  phy <- subset_samples(pdcy_phy, !is.na(sample_data(pdcy_phy)[[i]]))
  rar <- rarefy_even_depth(phy, rngseed = 1942890)
  dis <- vegdist(as.data.frame(t(as.matrix(otu_table(rar)))))
  bdiv_pdc_i[[i]] <- adonis2(as.formula(paste("dis ~ Group * ", i)),
                             data = as(sample_data(rar), "data.frame"),
                             permutations = 9999)["Pr(>F)"]
}

# Grab p-values into data frame
bdiv_pdc_i_p <- data.frame(
  Var = inf_scfa_vars,
  pGroup = sapply(inf_scfa_vars, function(x) bdiv_pdc_i[[x]][1,]),
  pVar = sapply(inf_scfa_vars, function(x) bdiv_pdc_i[[x]][2,]),
  pInteraction = sapply(inf_scfa_vars, function(x) bdiv_pdc_i[[x]][3,]))
```

Any significant results?

```
kable(subset(bdiv_pdc_i_p, pInteraction < 0.1),
     row.names = FALSE, booktabs = TRUE, linesep = "", digits = 3)
```

Var	pGroup	pVar	pInteraction
pIL6	0	0.409	0.035

Same, but with variables of interest split to binary by median:

```
# Make a df of binary variables (with new versions of the two already binary SCFA vars)
bd_meta_bin <- sapply(
  sub("_binary", "", inf_scfa_vars),
       function (x) sample_data(pdcy_phy)[[x]] >
    median(sample_data(pdcy_phy)[[x]], na.rm = TRUE))
colnames(bd_meta_bin) <- paste(colnames(bd_meta_bin), "2cat", sep = "_")

pdcy_phy_bd <- pdcy_phy
sample_data(pdcy_phy_bd) <- cbind(sample_data(pdcy_phy_bd), bd_meta_bin)

bdiv_pdc_i2 <- list()

for(i in colnames(bd_meta_bin)){
  phy <- subset_samples(pdcy_phy_bd, !is.na(sample_data(pdcy_phy_bd)[[i]]))
  rar <- rarefy_even_depth(phy, rngseed = 1942890)
  dis <- vegdist(as.data.frame(t(as.matrix(otu_table(rar)))))
  bdiv_pdc_i2[[i]] <- adonis2(as.formula(paste("dis ~ Group * ", i)),
                              data = as(sample_data(rar), "data.frame"),
                              permutations = 9999)["Pr(>F)"]
}

# Grab p-values into data.frame form
bdiv_pdc_i2_p <- data.frame(
  Var = colnames(bd_meta_bin),
  pGroup = sapply(colnames(bd_meta_bin), function(x) bdiv_pdc_i2[[x]][1,]),
  pVar = sapply(colnames(bd_meta_bin), function(x) bdiv_pdc_i2[[x]][2,]),
  pInteraction = sapply(colnames(bd_meta_bin), function(x) bdiv_pdc_i2[[x]][3,]))
```

Any significant results?

```
kable(subset(bdiv_pdc_i2_p, pInteraction < 0.1),
     row.names = FALSE, booktabs = TRUE, linesep = "", digits = 3)
```

Var	pGroup	pVar	pInteraction
butyric_acid_2cat	0	0.000	0.040
SNGAL_2cat	0	0.000	0.062
Szonulin_2cat	0	0.001	0.038

Additional file 7B

Combine and export the interaction results.

```
# Combine result data frames
bdiv_interact_res <- cbind(bdiv_pdc_i_p, bdiv_pdc_i2_p)

# Fix variable labels
bdiv_interact_res$Var <- sub(" tool", " ", varLabelFix(bdiv_interact_res$Var))

# Export
write.csv(bdiv_interact_res, "Outputs/add6B_bdiv_interactions.csv", row.names = FALSE)
```

3.3 Revision: Additional comparisons of PD-related variables and diversity

Alpha diversity

```
# List of PD-only, binary or continuous variables from earlier clinical correlations
conf_vars_pd <- setdiff(conf_vars, conf_vars_no_pd)
# Drop DUODOPA since only two patients are on this
conf_vars_pd <- conf_vars_pd[-grep("DUODOPA", conf_vars_pd)]
# Drop mg medication variables for simplicity
conf_vars_pd <- conf_vars_pd[-grep("_mg", conf_vars_pd)]

# Set up data
pd_divcor_df <- subset(pdcy_meta,
                       Group == "P")[, c("Shannon",
                                         "InvSimpson",
                                         conf_vars_pd)]

# Convert binary factors to numeric
for(i in conf_vars_pd){
  if(is.factor(pd_divcor_df[,i])){
    pd_divcor_df[,i] <- as.numeric(pd_divcor_df[,i])
  }
}

# Calculate correlations with diversity measures
adiv_cors_PD_clin <- divCors(pd_divcor_df, conf_vars_pd)

# Anything significant?
kable_styling(kable(subset(adiv_cors_PD_clin, ShanP < 0.1 | InvSP < 0.1),
                    booktabs = TRUE, linesep = "", digits = 4,
                    row.names = FALSE), font_size = 8)
```

Var	ShanCor	ShanP	InvSCor	InvSP
akinetic_rigid_score_poletti_OFF	0.3616	0.0078	0.4134	0.0021
Hoehn_and_Yahr_OFF	0.3118	0.0230	0.3705	0.0063
meds_dopamine_agonist	-0.2476	0.0684	-0.2520	0.0634
pigd_score_jankovic_OFF	0.2949	0.0321	0.3720	0.0061
tremor_by_pigd_jankovic_OFF	0.2329	0.1329	0.3233	0.0344
tremor_score_jankovic_OFF	0.2715	0.0492	0.3743	0.0058
tremor_score_poletti_OFF	0.2724	0.0485	0.3906	0.0038
UPDRS_I_total	0.2796	0.0387	0.2265	0.0963
UPDRS_II_total	0.3468	0.0095	0.4169	0.0015
UPDRS_III_total_OFF	0.4212	0.0017	0.4912	0.0002
UPDRS_IV_total	0.4070	0.0020	0.5730	0.0000

```
# Rearrange df for plotting
pd_divcor_df_p <- melt(pd_divcor_df, id.vars = conf_vars_pd)
```

Scatterplots for variables that are significant for both diversity measures, showing only inverse Simpson which tends to have lower p-values.

```
adiv_cors_scatter_pd <- lapply(subset(adiv_cors_PD_clin,
                                      ShanP < 0.05 &
                                        InvSP < 0.05)$Var,
               function(x) ggplot(subset(pd_divcor_df_p,
                                         variable == "InvSimpson"),
```

```
                              aes_string(x = x, y = "value")) +
                  geom_point(shape = 17, size = 1) +
                  theme_bw(base_size = 6) +
                  ylab("Inverse Simpson") +
                  xlab(sub(" OFF", "", varLabelFix(x))) +
                  theme(panel.grid = element_blank(),
                        axis.text = element_text(color = "black"),
                        aspect.ratio = 1))

adiv_cors_scatter_pd[[1]] <- adiv_cors_scatter_pd[[1]] +
  ggtitle("B") +
  theme(plot.title = element_text(size = 12, face = "bold"))
adiv_cors_scatter_pd[[2]] <- adiv_cors_scatter_pd[[2]] +
  ggtitle("")
adiv_cors_scatter_pd[[3]] <- adiv_cors_scatter_pd[[3]] +
  ggtitle("")

adiv_cors_scatters_pd <- do.call("arrangeGrob",
                                list(grobs = adiv_cors_scatter_pd,
                                  ncol = 3,
                                  heights = c(1, 0.85, 0.85)))
grid.arrange(adiv_cors_scatters_pd)
```

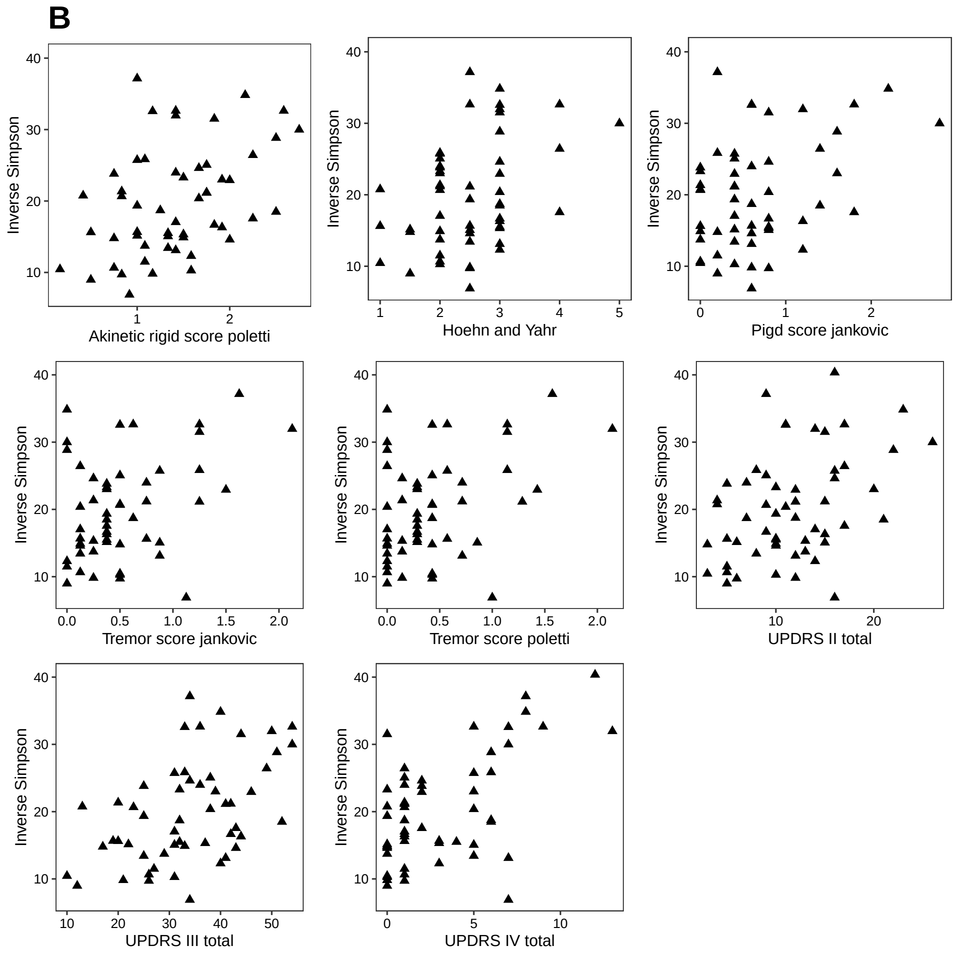

Beta diversity

```
bdiv_pd_clin <- list()

for(i in conf_vars_pd){
  phy <- subset_samples(pdcy_phy_P, !is.na(sample_data(pdcy_phy_P)[[i]]))
  rar <- rarefy_even_depth(phy, rngseed = 9377916)
  dis <- vegdist(as.data.frame(t(as.matrix(otu_table(rar)))))
  bdiv_pd_clin[[i]] <- adonis(as.formula(paste("dis ~", i)),
                              data = as(sample_data(rar), "data.frame"),
                              permutations = 9999)$aov.tab
}

# Grab p-values
bdiv_pd_clin_p <- sapply(conf_vars_pd, function(x)
  bdiv_pd_clin[[x]][["Pr(>F)"]][1])

# Make data frame
bdiv_pd_clin_p <- data.frame(Var = names(bdiv_pd_clin_p),
                             pVar = bdiv_pd_clin_p)

# What is significant or close?
kable_styling(kable(subset(bdiv_pd_clin_p, pVar < 0.1),
                    booktabs = TRUE, linesep = "", digits = 4,
                    row.names = FALSE), font_size = 8)
```

Var	pVar
age_NMS_onset	0.0934
akinetic_rigid_score_poletti_OFF	0.0049
calculated_time_from_nms_onset	0.0676
Hoehn_and_Yahr_OFF	0.0817
meds_COMT_inhibitor	0.0001
meds_dopa	0.0259
meds_dopamine_agonist	0.0230
pigd_score_jankovic_OFF	0.0103
tremor_score_poletti_OFF	0.0576
UPDRS_II_total	0.0007
UPDRS_III_total_OFF	0.0003
UPDRS_IV_total	0.0007

Combined summary table of alpha- and beta diversity p-values:

```
divs_PD_clin <- adiv_cors_PD_clin[,c("Var", "ShanP", "InvSP")]
colnames(divs_PD_clin) <- c("Var",
                            "Pearson for\nShannon",
                            "Pearson for\ninverse\nSimpson")
divs_PD_clin$adonis <- bdiv_pd_clin_p[rownames(divs_PD_clin),
                                      "pVar"]
divs_PD_clin$Var <- sub("calculated_", "",
                        sub("_OFF", "", divs_PD_clin$Var))
divs_PD_clin <- melt(divs_PD_clin)
divs_PD_clin$Type <- ifelse(grepl("adonis", divs_PD_clin$variable),
                            "Beta\ndiversity", "Alpha\ndiversity")
divs_PD_clin$P_label <- sub("0.000", "<0.001",
      sprintf("%.3f", divs_PD_clin$value))
```

```r
divs_PD_clin$VarLabel <- varLabelFix(divs_PD_clin$Var)

divs_PD_summary_plot <- ggplot(divs_PD_clin,
                               aes(x = variable, y = VarLabel,
                                   fill = value < 0.05)) +
  geom_tile(color = "gray30") +
  scale_fill_manual(values = c("gray70", "#ffd8c8")) +
  theme_bw(base_size = 7) +
  geom_text(aes(label = P_label), size = 2) +
  ggtitle("A") +
  facet_grid(~Type, scale = "free", space = "free") +
  theme(legend.position = "none",
        axis.text = element_text(color = "black"),
        axis.title = element_blank(),
        panel.grid = element_blank(),
        plot.title = element_text(size = 12, face = "bold"))
divs_PD_summary_plot
```

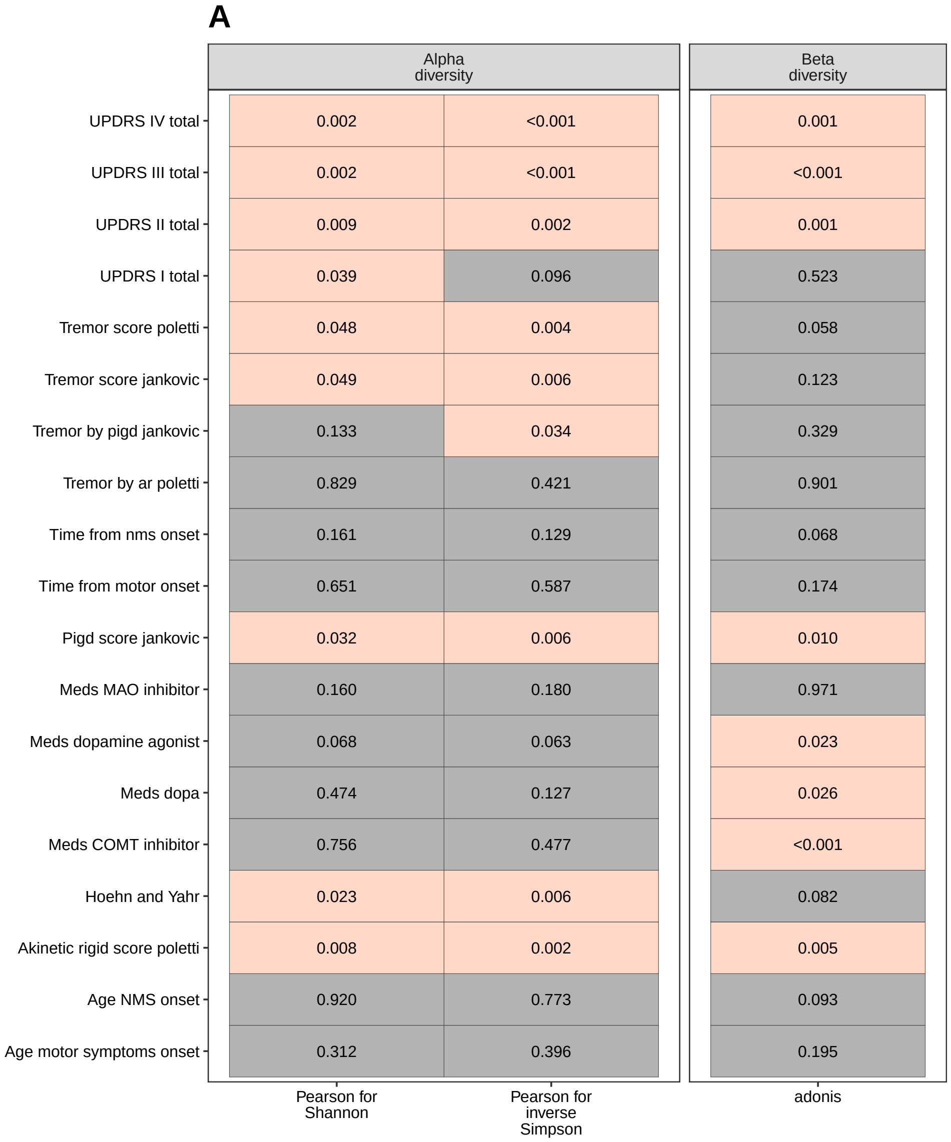

Plots for individual significant variables:

```
pdcy_phy_P_r <- rarefy_even_depth(pdcy_phy_P, rngseed = 9377916)
pdcy_phy_P_ord <- ordinate(pdcy_phy_P_r, method = "NMDS",
                           distance = "bray",
                           trace = FALSE, trymax = 999)

bdiv_pd_clin_df <- data.frame(
  pdcy_phy_P_ord$points[,c("MDS1", "MDS2")],
  sample_data(pdcy_phy_P_r))

library("viridis")

# Plot variables with p < 0.05
bdiv_pd_clin_plots <- do.call(
  "arrangeGrob",
  c(lapply(subset(bdiv_pd_clin_p, pVar < 0.05)$Var, function(x)
    if(length(unique(bdiv_pd_clin_df[,x])) == 2){
      ggplot(bdiv_pd_clin_df, aes_string(x = "MDS1", y = "MDS2",
                                          fill = x)) +
        geom_point(color = "black", shape = 21) +
        theme_bw(base_size = 7) +
        coord_fixed() +
        ggtitle(varLabelFix(x)) +
        scale_fill_manual(values = c("#000004FF", "#FE9F6DFF"),
                          labels = c("no", "yes")) +
        theme(panel.grid = element_blank(),
              plot.title = element_text(size = 8),
              legend.key.size = unit(3, "mm"),
              legend.title = element_blank())
    }
    else{
      ggplot(subset(bdiv_pd_clin_df,
                    !is.na(bdiv_pd_clin_df[,x])),
             aes_string(x = "MDS1", y = "MDS2",
                        fill = x)) +
        geom_point(color = "black", shape = 21) +
        theme_bw(base_size = 7) +
        coord_fixed() +
        ggtitle(sub(" OFF", "", varLabelFix(x))) +
        scale_fill_viridis_b(option = "A") +
        theme(panel.grid = element_blank(),
              plot.title = element_text(size = 8),
              legend.key.size = unit(3, "mm"),
              legend.title = element_blank())}),
    ncol = 3))

grid.arrange(bdiv_pd_clin_plots)
```

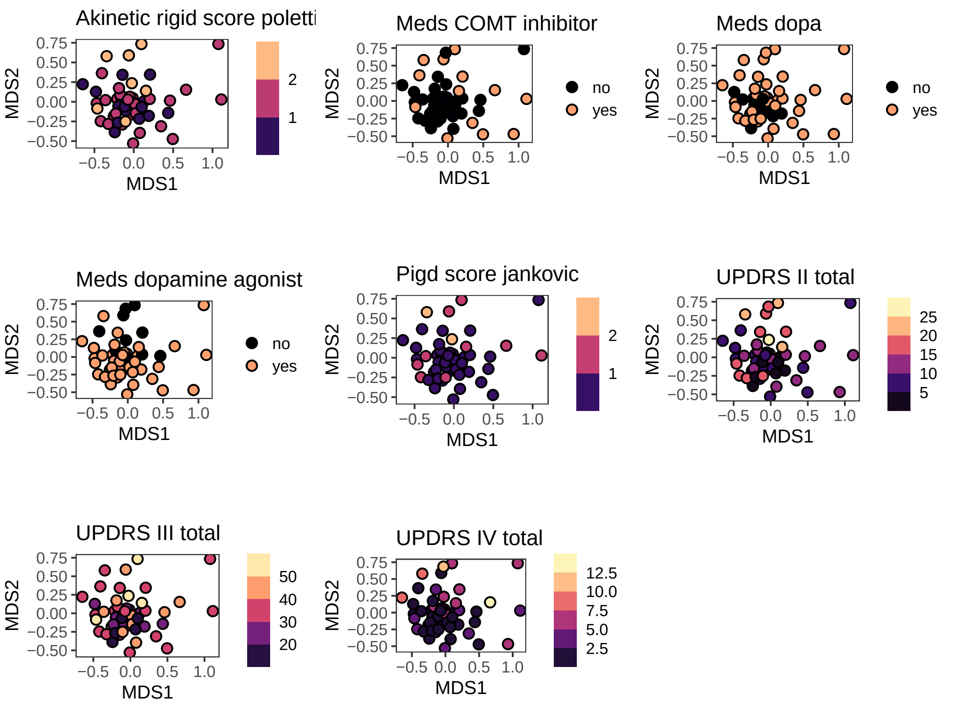

Additional file 5

Combine the diversity/severity plots for export:

```
div_pd_combo_plot <- arrangeGrob(
  arrangeGrob(
    divs_PD_summary_plot,
    adiv_cors_scatters_pd,
    nrow = 1, widths = c(0.45, 0.55)),
  ggplot() + theme_void() + ggtitle("C") +
    theme(plot.title = element_text(size = 12, face = "bold",
                                    hjust = 0.05)),
  bdiv_pd_clin_plots,
  ncol = 1, heights = c(0.485, 0.03, 0.485))

ggsave(div_pd_combo_plot,
       filename = "Outputs/add8_diversity_pd_severity.pdf",
       width = maxwd, height = maxhi, units = "mm")
```

3.4 Enterotypes

Look for differences in levels of variables of interest in relation to enterotypes. Based on the previous publication on this data, the distributions of enterotypes in the PD and control groups were slightly different, mainly with more PD subjects in the Firmicutes and less in the Prevotella enterotype. Three samples did not have an enterotype classification due to being excluded from the follow-up analyses (Aho et al. 2019), and are therefore missing from these comparisons.

In this specific subset, the numbers were the following:

```
kable(table(pdcy_meta$Enterotype, pdcy_meta$Group), booktabs = TRUE)
```

	C	P
ET_B	8	8
ET_F	25	35
ET_P	22	10

The difference in distributions of enterotypes between groups was statistically significant (Fisher's exact test p = 0.045).

Additional file 9A

Export n of enterotypes per group:

```
write.csv(table(pdcy_meta$Enterotype, pdcy_meta$Group),
          "Outputs/add9A_et_summary.csv")
```

Run tests (Kruskal-Wallis rank sum test) for significant differences in concentrations of stool and plasma markers and SCFAs between enterotypes for

1. full data
2. control subjects only
3. PD patients only,

and collect the results into one data frame:

```
# Generic testing function with Kruskal-Wallis
kwTestET <- function(df){
  res <- data.frame(Variable = inf_scfa_vars,
                    pVal = sapply(inf_scfa_vars, function(x)
                      kruskal.test(as.formula(paste(x, "~Enterotype")), df)$p.value))
  res$pAdj <- c(
    p.adjust(res[scfa_vars, "pVal"], method = "fdr"),
    p.adjust(res[infs_vars, "pVal"], method = "fdr"),
    p.adjust(res[infp_vars, "pVal"], method = "fdr"))
  return(res)
}

# Full data
kw_et <- kwTestET(pdcy_meta)
# Only control subjects
kw_et_c <- kwTestET(subset(pdcy_meta, Group == "C"))
# Only PD
kw_et_p <- kwTestET(subset(pdcy_meta, Group == "P"))

# Combine results
kw_et_combo <- data.frame(Variable = kw_et$Variable,
                          pVal_all = kw_et$pVal,
                          pAdj_all = kw_et$pAdj,
                          pVal_C = kw_et_c$pVal,
                          pAdj_C = kw_et_c$pAdj,
                          pVal_PD = kw_et_p$pVal,
                          pAdj_PD = kw_et_p$pAdj)
```

Additional file 9B

Significant results from the above comparisons:

```r
# Variables with p < 0.05 for at least one of the three datasets
kable(kw_et_combo[rowSums(kw_et_combo[, c(2, 4, 6)] < 0.05) > 0, ],
      booktabs = TRUE, linesep = "", digits = 3, row.names = FALSE)
```

Variable	pVal_all	pAdj_all	pVal_C	pAdj_C	pVal_PD	pAdj_PD
propionic_acid	0.044	0.159	0.348	0.770	0.161	0.377
butyric_acid	0.045	0.159	0.423	0.770	0.013	0.093
SNGAL	0.001	0.008	0.002	0.008	0.419	0.936
Szonulin	0.114	0.376	0.000	0.003	0.634	0.936

```r
# Fix variable names for export
kw_et_combo$Variable <- varLabelFix(kw_et_combo$Variable)

# Export the full table
write.csv(kw_et_combo, "Outputs/add9B_enterotype_pvals.csv", row.names = FALSE)
```

Run post-hoc tests (pairwise Wilcoxon rank sum test) to see which pairs of enterotypes differ:

NGAL

```r
et_ngal_c <- as.data.frame(
  pairwise.wilcox.test(x = subset(pdcy_meta, Group == "C")$SNGAL,
                       g = subset(pdcy_meta, Group == "C")$Enterotype)$p.value)
et_ngal_c$Comp <- rownames(et_ngal_c)
et_ngal_c <- melt(et_ngal_c)
colnames(et_ngal_c) <- c("ET1", "ET2", "pVal")

kable(subset(et_ngal_c, !is.na(pVal)), booktabs = TRUE, digits = 3, row.names = FALSE)
```

ET1	ET2	pVal
ET_F	ET_B	0.204
ET_P	ET_B	0.001
ET_P	ET_F	0.027

Zonulin

```r
et_zon_c <- as.data.frame(
  pairwise.wilcox.test(x = subset(pdcy_meta, Group == "C")$Szonulin,
                       g = subset(pdcy_meta, Group == "C")$Enterotype)$p.value)
et_zon_c$Comp <- rownames(et_zon_c)
et_zon_c <- melt(et_zon_c)
colnames(et_zon_c) <- c("ET1", "ET2", "pVal")

kable(subset(et_zon_c, !is.na(pVal)), booktabs = TRUE, digits = 3, row.names = FALSE)
```

ET1	ET2	pVal
ET_F	ET_B	0.374
ET_P	ET_B	0.002
ET_P	ET_F	0.002

Butyric acid

```r
et_but_p <- as.data.frame(
  pairwise.wilcox.test(x = subset(pdcy_meta, Group == "P")$butyric_acid,
                       g = subset(pdcy_meta, Group == "P")$Enterotype)$p.value)
et_but_p$Comp <- rownames(et_but_p)
```

```r
et_but_p <- melt(et_but_p)
colnames(et_but_p) <- c("ET1", "ET2", "pVal")
kable(subset(et_but_p, !is.na(pVal)), booktabs = TRUE, digits = 3, row.names = FALSE)
```

ET1	ET2	pVal
ET_F	ET_B	0.939
ET_P	ET_B	0.017
ET_P	ET_F	0.017

Fig 6

Combine the results, make a plot and export:

```r
# Handmade DF for p-value annotations
et_pvals <- rbind(
  data.frame(subset(et_but_p, pVal < 0.05),
             Group = "P",
             variable = "butyric_acid"),
  data.frame(subset(et_ngal_c, pVal < 0.05),
             Group = "C",
             variable = "SNGAL"),
  data.frame(subset(et_zon_c, pVal < 0.05),
             Group = "C",
             variable = "Szonulin"))
et_pvals$stars <- stars.pval(et_pvals$pVal)
et_pvals$xtext <- c(2, 2.5)
et_pvals$x1 <- c(1, 2)
et_pvals$y <- c(2.6, 3.1, 325, 375, 190, 225)
et_pvals$ytext <- c( 2.7, 3.2, 330, 380, 195, 230)

et_df <- melt(pdcy_meta[,c("Group", "Enterotype", "butyric_acid", "SNGAL", "Szonulin")])
et_df <- subset(et_df, !is.na(Enterotype))
et_df[et_df$variable == "butyric_acid", "value"] <-
  et_df[et_df$variable == "butyric_acid", "value"]/1000

et_plots <- ggplot(et_df, aes(x = Enterotype, y = value)) +
  geom_boxplot(width = 0.7, outlier.size = 0.5) +
  theme_bw(base_size = 10) +
  xlab("Enterotype") +
  ylab(NULL) +
  scale_x_discrete(labels = c("Bacteroides", "Firmicutes", "Prevotella")) +
  scale_y_continuous(expand = expansion(mult = 0.1)) +
  facet_grid(variable ~ Group, scales = "free",
             labeller = labeller(
               variable = setNames(c("Butyric acid\n(mmol/l)",
                                     "Stool NGAL\n(pg/mg total protein)",
                                     "Stool zonulin\n(pg/mg total protein)"),
                                   c("butyric_acid", "SNGAL", "Szonulin")),
               Group = pdc_labels)) +
  geom_text(aes(x = xtext, y = ytext, label = stars),
            et_pvals, inherit.aes = FALSE, size = 4) +
  geom_segment(aes(x = x1, xend = 3, y = y, yend = y),
               et_pvals, inherit.aes = FALSE) +
    theme(panel.grid = element_blank(),
```

```
           axis.text = element_text(color = "black"),
           strip.background = element_rect(fill = "white"),
           axis.text.x = element_text(angle = 25, vjust = 1, hjust = 1, face = "italic"))
et_plots
```

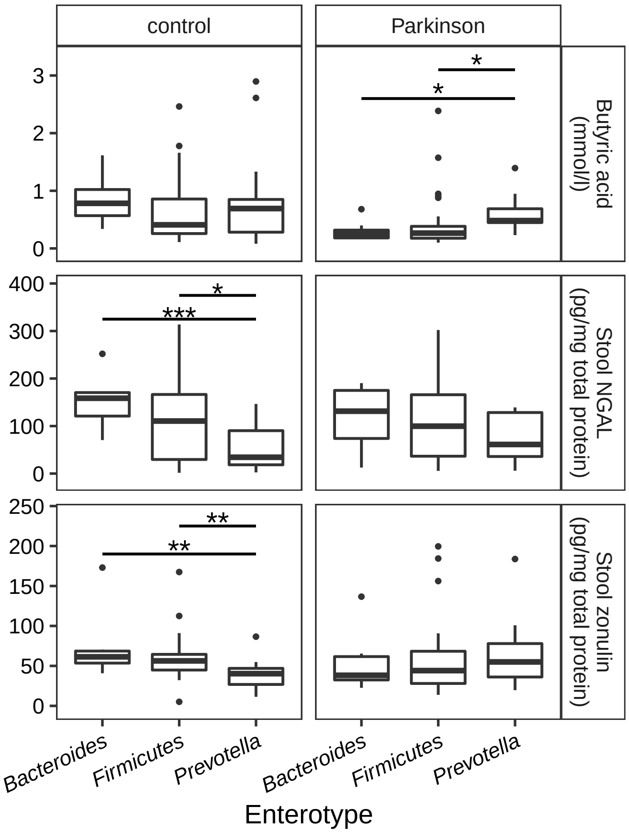


```
ggsave(et_plots, filename = "Outputs/fig6_enterotypes.pdf",
       width = maxwd/2, height = maxhi/2, units = "mm")
embedFonts("Outputs/fig6_enterotypes.pdf")
```

3.5 Differential abundance with DESeq2

Look for bacterial genera and families associated with specific markers/SCFAs with DESeq2, focusing on variables that were particularly interesting based on the other comparisons: stool calprotectin, NGAL, zonulin, and the three most common SCFAs. Run the comparisons in four different ways:

1. Taxon ~ variable, full data
2. Taxon ~ variable + sex + Rome III 9-15 score + PD/C, full data
3. Taxon ~ variable, control subjects only
4. Taxon ~ variable, PD patients only

Set up data and functions for testing:

```
# Functions

# One variable, Taxon ~ variable of interest
ds2_1var <- function(phyloObj, v){
  phyloObj <- prune_samples(as.vector(!is.na(sample_data(phyloObj)[,v])), phyloObj)
  ds <- phyloseq_to_deseq2(phyloObj, as.formula(paste("~", v)))
  ds <- DESeq(ds, fitType = "parametric", sfType = "poscounts")
```

```r
  dsres <- cbind(data.frame(results(ds)), as(tax_table(phyloObj), "matrix"))

  return(dsres)
}

# Confounder-corrected:
# Taxon ~ variable of interest + sex + Rome III score + PD/control
# (grabbing results for the variable of interest only!)
ds2_1var_pdc <- function(phyloObj, v){
  phyloObj <- prune_samples(as.vector(!is.na(sample_data(phyloObj)[,v])), phyloObj)
  ds <- phyloseq_to_deseq2(phyloObj, as.formula(
    paste("~", v, "+ sex + Rome_III_constip_defec_sumscore_9.15 + Group")))
  ds <- DESeq(ds, fitType = "parametric", sfType = "poscounts")
  dsres <- cbind(data.frame(results(ds, name = v)),
                 as(tax_table(phyloObj), "matrix"))

  return(dsres)
}

# Phyloseq objects

# Function for trimming rare taxa and unclassifieds from the phyloseq objects
ds2trim <- function(phyloIn){
  lv <- tail(colnames(tax_table(phyloIn)), n = 1)
  phyloIn <- filter_taxa(phyloIn, function(x) sum(x > 5) > nsamples(pdcy_phy)/2,
                         prune = TRUE)
  prune_taxa(as.vector(tax_table(phyloIn)[, lv] != "unclassified"), phyloIn)
}

# Full data
pdcy_phy_gen_trim <- ds2trim(pdcy_phy_gen)
pdcy_phy_fam_trim <- ds2trim(pdcy_phy_fam)

# Make the PD-only and C-only subsets by subsetting these objects,
# so that all comparisons have the same set of taxa:

# Controls only
pdcy_phy_C_gen_trim <- subset_samples(pdcy_phy_gen_trim, Group == "C")
pdcy_phy_C_fam_trim <- subset_samples(pdcy_phy_fam_trim, Group == "C")

# PD only
pdcy_phy_P_gen_trim <- subset_samples(pdcy_phy_gen_trim, Group == "P")
pdcy_phy_P_fam_trim <- subset_samples(pdcy_phy_fam_trim, Group == "P")
```

3.5.1 SCFAs

Run comparisons for SCFAs:

```r
# Variables of interest
da_scfa_vars <- c("acetic_acid", "butyric_acid", "propionic_acid")

# Family level comparisons
fam_scfa <- do.call("rbind",
                    c(lapply(da_scfa_vars,
```

```
                              function(x) data.frame(ds2_1var(pdcy_phy_fam_trim, x),
                                                     SCFA = x,
                                                     Subset = "all")),
                       lapply(da_scfa_vars,
                              function(x) data.frame(ds2_1var_pdc(pdcy_phy_fam_trim, x),
                                                     SCFA = x,
                                                     Subset = "pdc")),
                       lapply(da_scfa_vars,
                              function(x) data.frame(ds2_1var(pdcy_phy_C_fam_trim, x),
                                                     SCFA = x,
                                                     Subset = "C")),
                       lapply(da_scfa_vars,
                              function(x) data.frame(ds2_1var(pdcy_phy_P_fam_trim, x),
                                                     SCFA = x,
                                                     Subset = "P"))))

# Genus level comparisons
gen_scfa <- do.call("rbind",
                    c(lapply(da_scfa_vars,
                              function(x) data.frame(ds2_1var(pdcy_phy_gen_trim, x),
                                                     SCFA = x,
                                                     Subset = "all")),
                      lapply(da_scfa_vars,
                              function(x) data.frame(ds2_1var_pdc(pdcy_phy_gen_trim, x),
                                                     SCFA = x,
                                                     Subset = "pdc")),
                      lapply(da_scfa_vars,
                              function(x) data.frame(ds2_1var(pdcy_phy_C_gen_trim, x),
                                                     SCFA = x,
                                                     Subset = "C")),
                      lapply(da_scfa_vars,
                              function(x) data.frame(ds2_1var(pdcy_phy_P_gen_trim, x),
                                                     SCFA = x,
                                                     Subset = "P"))))
```

Collect and reorganize results:

```
# List taxa significant in any comparison
fam_scfa_res_df <- subset(fam_scfa,
                          Family %in% unique(subset(fam_scfa, padj < 0.05)$Family))
fam_scfa_res_df$Taxon <- fam_scfa_res_df$Family
fam_scfa_res_df$Genus <- NA

gen_scfa_res_df <- subset(gen_scfa,
                          Genus %in% unique(subset(gen_scfa, padj < 0.05)$Genus))
gen_scfa_res_df$Taxon <- gen_scfa_res_df$Genus

# Combined data frame
scfa_res_df <- rbind(data.frame(fam_scfa_res_df, Level = "Family"),
                     data.frame(gen_scfa_res_df, Level = "Genus"))

# Reorganize for plotting
scfa_res_df_m <- melt(scfa_res_df)
scfa_res_df_c <- dcast(scfa_res_df_m, SCFA + Taxon + Subset + Level ~ variable)
```

```
scfa_res_df_c$pStar <- stars.pval(scfa_res_df_c$padj)
```

Check for outlierish log2 fold changes:

```
# Check the spreads of values for the log2 fc
do.call("grid.arrange",
        c(lapply(da_scfa_vars, function(x)
                     ggplot(subset(scfa_res_df_c, SCFA == x),
                            aes(x = SCFA, y = log2FoldChange)) +
                     geom_boxplot() +
                     xlab(NULL) +
                     theme_bw(base_size = 10)), nrow = 1))
```

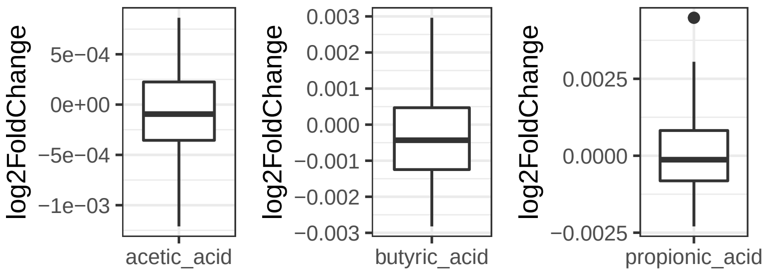

No major outliers. Export the final table:

Additional file 11A

```
# Rename the group levels
scfa_res_df_c$Subset <- factor(scfa_res_df_c$Subset, levels = c("all", "pdc", "C", "P"))
levels(scfa_res_df_c$Subset) <- c("full data",
                                  "full data, corrected for PD/C and confounders",
                                  "control only", "PD only")
# Nicer names for the SCFA var
scfa_res_df_c$SCFA <- capitalize(sub("_", " ", sub("", "", scfa_res_df_c$SCFA)))

# Export full results
write.csv(scfa_res_df_c, "Outputs/add11A_ds2_scfa.csv", row.names = FALSE)
```

Fig 7: SCFAs

Summary plot of the results:

```
# Plotting function
inf_heatmap <- function(df, v){
  ggplot(subset(df, df[, 1] == v),
            aes(x = Subset, y = Taxon, fill = log2FoldChange)) +
    geom_tile(color = "gray30") +
    ggtitle(v) +
    theme_bw(base_size = 9) +
    scale_fill_gradient2(low = "black", mid = "white", high = "firebrick4",
                         name = "log2\nfold\nchange") +
    geom_text(aes(label = pStar), size = 5) +
    theme(legend.position = "bottom",
          axis.title = element_blank(),
          panel.grid = element_blank(),
          axis.text.y = element_blank(),
          axis.text.x = element_text(color = "black"),
          axis.ticks = element_blank(),
```

```r
          legend.key.width = unit(0.55, "cm"),
          legend.title = element_blank())
}

# Genus-level df for plotting
scfa_res_df_c_gen <- subset(scfa_res_df_c, Level == "Genus")

# Rename the group levels for plotting
levels(scfa_res_df_c_gen$Subset) <- c("full\ndata",
                                 "full\ndata,\ncorrected\nfor PD/C and\nconfounders",
                                 "control\nonly", "PD\nonly")

# Nicer names & reordering for the taxon list
scfa_res_df_c_gen$Taxon <- gsub("_", " ", scfa_res_df_c_gen$Taxon)
scfa_res_df_c_gen$Taxon <- factor(scfa_res_df_c_gen$Taxon,
                              levels = rev(sort(unique(scfa_res_df_c_gen$Taxon))))

# Workaround to create y-axis labels + legend title
scfa_y <- ggplot(subset(scfa_res_df_c_gen,
                        Subset == levels(scfa_res_df_c_gen$Subset)[2]),
                 aes(x = Subset, y = Taxon, fill = log2FoldChange)) +
  geom_tile(color = "white", width = 0) +
  theme_bw(base_size = 9) +
  ggtitle(" ") +
  scale_fill_gradient2(low = "white", mid = "white", high = "white",
                       name = "log2\nfold\nchange") +
  theme(legend.position = "bottom",
        legend.key.size = unit(0, "mm"),
        legend.text = element_blank(),
        legend.margin = margin(r = 30, b = 10),
        axis.title = element_blank(),
        panel.grid = element_blank(),
        panel.background = element_rect(color = "white"),
        panel.border = element_rect(color = "white"),
        axis.text.x = element_text(color = "white"),
        axis.text.y = element_text(color = "black", face = "italic",
                                   margin = margin(r = -4)),
        axis.ticks = element_blank())

# Put together plots
da_scfa_plots <- arrangeGrob(
  scfa_y,
  inf_heatmap(scfa_res_df_c_gen, "Acetic acid") +
    scale_fill_gradient2(low = "gray25", mid = "white", high = "firebrick4",
                         name = "log2\nfold\nchange", breaks = c(-0.001, 0, 0.0008),
                         labels = function(x) format(x, scientific = FALSE)),
  inf_heatmap(scfa_res_df_c_gen, "Butyric acid") +
    scale_fill_gradient2(low = "gray20", mid = "white", high = "firebrick4",
                         name = "log2\nfold\nchange", breaks = c(-0.002, 0, 0.002)),
  inf_heatmap(scfa_res_df_c_gen, "Propionic acid"),
  nrow = 1, widths = c(0.95, 1.5, 1.5, 1.5))

grid.arrange(da_scfa_plots)
```

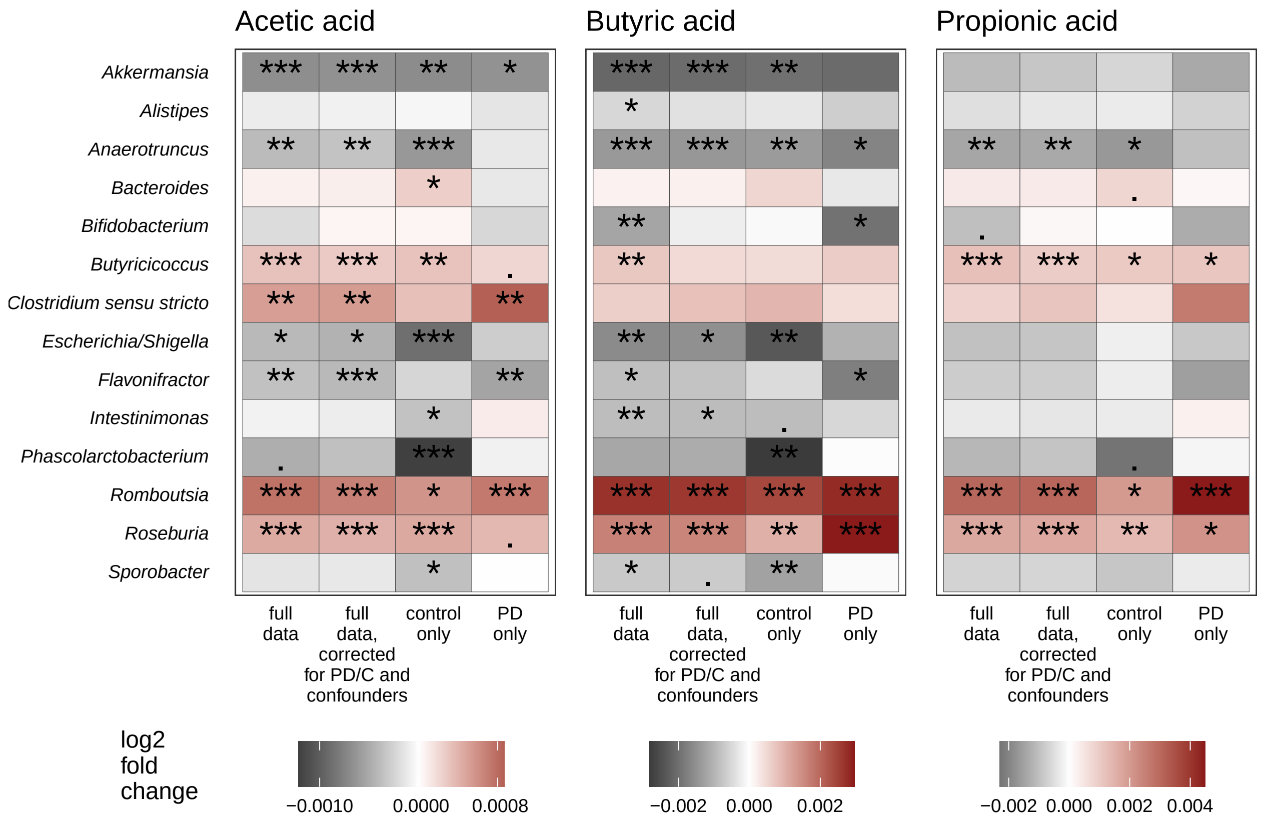

Additional file 10

Scatterplots of the most interesting taxa from the results.

```
# Set up a relative abundance data.frame
ra_df_gen <- t(prop.table(as(otu_table(pdcy_phy_gen), "matrix"), 2)*100)
ra_df <- cbind(ra_df_gen, sample_data(pdcy_phy_gen)[, c("Group", inf_scfa_vars)])

# Remove the doubled "unclassified" column
ra_df <- ra_df[, -(grep("unclassified", colnames(ra_df)))]

# Scatterplot function for relative abundances
ra_scatter <- function(df, xvar, taxon){
  ggplot(df, aes_string(x = xvar, y = taxon)) +
    geom_point(size = 0.75) +
    theme_bw(base_size = 8) +
    facet_grid(~Group) +
    ylab("Relative abundance (%)") +
    ggtitle(taxon) +
    theme(panel.grid = element_blank(),
          axis.text = element_text(color = "black"),
          plot.title = element_text(face = "italic"))
}
```

Since the three SCFAs had overall similar patterns for the taxa, only plot each taxon once:

```
# Significant taxa for butyric acid
buty_sigs <- as.character(unique(
  subset(scfa_res_df_c_gen, SCFA == "Butyric acid" & padj < 0.1)$Taxon))
```

```r
# Significant taxa for acetic acid that are not on the butyric acid list
acet_sigs <- setdiff(as.character(unique(
  subset(scfa_res_df_c_gen, SCFA == "Acetic acid" & padj < 0.1)$Taxon)), buty_sigs)

# Any significant taxa for propionic acid not on the butyric acid or acetic acid list?
setdiff(setdiff(as.character(unique(
  subset(scfa_res_df_c_gen, SCFA == "Propionic acid" & padj < 0.1)$Taxon)),
  buty_sigs), acet_sigs)
```

```
## character(0)
```

```r
# Fix the problematically named Escherichia/Shigella
buty_sigs[grep("Escher", buty_sigs)] <- "`Escherichia/Shigella`"
# Fix the spaces in Clostridium sensu stricto
acet_sigs[grep("Clost", acet_sigs)] <- gsub(" ", "_", acet_sigs[grep("Clost", acet_sigs)])

# Plot
scfa_da_scatters <- c(
  lapply(buty_sigs, function(x)
    ra_scatter(ra_df, "butyric_acid/1000", x) +
      xlab("Butyric acid (mmol/l)")),
  lapply(acet_sigs, function(x)
    ra_scatter(ra_df, "acetic_acid/1000", x) +
      xlab("Acetic acid (mmol/l)")))
# Fix the title for 'Escherichia/Shigella'
scfa_da_scatters[[grep("Escher", buty_sigs)]]  <-
  scfa_da_scatters[[grep("Escher", buty_sigs)]] +
  ggtitle("Escherichia/Shigella")

# Arrange plots
scfa_da_scatters <- do.call(arrangeGrob, c(scfa_da_scatters, ncol = 3))
grid.arrange(scfa_da_scatters)
```

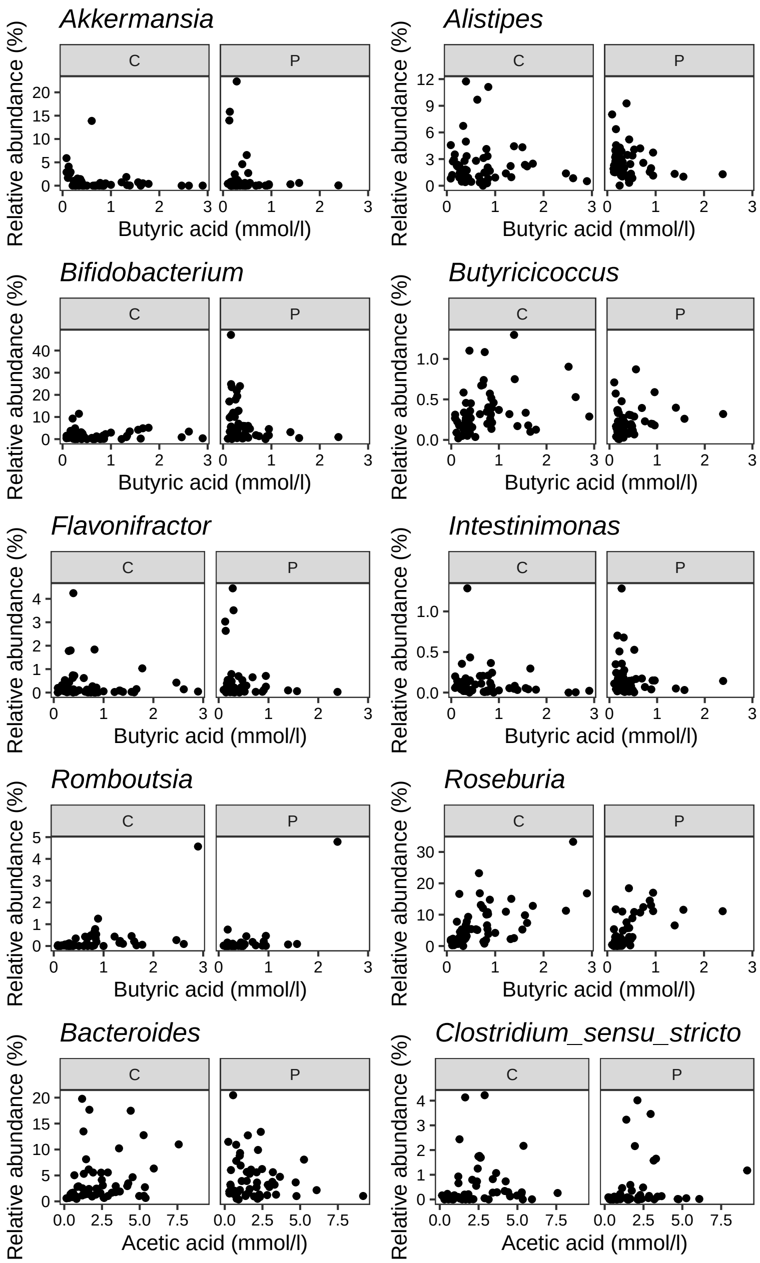


```r
# Export
ggsave(scfa_da_scatters,
       filename = "Outputs/add10_diffabund_scatters_scfas.pdf",
       width = maxwd, height = maxhi*0.9, units = "mm")
```

3.5.2 Inflammatory markers

Run comparisons for stool markers:

```r
# Variables of interest
da_inf_vars <- c("SNGAL", "Szonulin", "Scalprotectin")

# Family level comparisons
fam_inf <- do.call("rbind",
                   c(lapply(da_inf_vars,
```

```
                         function(x) data.frame(ds2_1var(pdcy_phy_fam_trim, x),
                                                Marker = x,
                                                Subset = "all")),
                   lapply(da_inf_vars,
                         function(x) data.frame(ds2_1var_pdc(pdcy_phy_fam_trim, x),
                                                Marker = x,
                                                Subset = "pdc")),
                   lapply(da_inf_vars,
                         function(x) data.frame(ds2_1var(pdcy_phy_C_fam_trim, x),
                                                Marker = x,
                                                Subset = "C")),
                   lapply(da_inf_vars,
                         function(x) data.frame(ds2_1var(pdcy_phy_P_fam_trim, x),
                                                Marker = x,
                                                Subset = "P"))))
# Genus level comparisons:
gen_inf <- do.call("rbind",
                  c(lapply(da_inf_vars,
                         function(x) data.frame(ds2_1var(pdcy_phy_gen_trim, x),
                                                Marker = x,
                                                Subset = "all")),
                   lapply(da_inf_vars,
                         function(x) data.frame(ds2_1var_pdc(pdcy_phy_gen_trim, x),
                                                Marker = x,
                                                Subset = "pdc")),
                   lapply(da_inf_vars,
                         function(x) data.frame(ds2_1var(pdcy_phy_C_gen_trim, x),
                                                Marker = x,
                                                Subset = "C")),
                   lapply(da_inf_vars,
                         function(x) data.frame(ds2_1var(pdcy_phy_P_gen_trim, x),
                                                Marker = x,
                                                Subset = "P"))))
```

Collect and reorganize results:

```
# List taxa significant in any comparison
fam_inf_res_df <- subset(fam_inf, Family %in% unique(subset(fam_inf, padj < 0.05)$Family))
fam_inf_res_df$Taxon <- fam_inf_res_df$Family
fam_inf_res_df$Genus <- NA

gen_inf_res_df <- subset(gen_inf, Genus %in% unique(subset(gen_inf, padj < 0.05)$Genus))
gen_inf_res_df$Taxon <- gen_inf_res_df$Genus

# Combined data frame
inf_res_df <- rbind(data.frame(fam_inf_res_df, Level = "Family"),
                    data.frame(gen_inf_res_df, Level = "Genus"))

# Reorganize for plotting
inf_res_df_m <- melt(inf_res_df)
inf_res_df_c <- dcast(inf_res_df_m, Marker + Taxon + Subset + Level ~ variable)
inf_res_df_c$pStar <- stars.pval(inf_res_df_c$padj)
inf_res_df_c$Taxon <- factor(inf_res_df_c$Taxon)
```

Check the spreads of values for the log2 fold changes:

```
# Check the spreads of values for the log2 fc
do.call("grid.arrange",
        c(lapply(da_inf_vars, function(x)
                  ggplot(subset(inf_res_df_c, Marker == x),
                         aes(x = Marker, y = log2FoldChange)) +
                  geom_boxplot() +
                  xlab(NULL) +
                  theme_bw(base_size = 10)), nrow = 1))
```

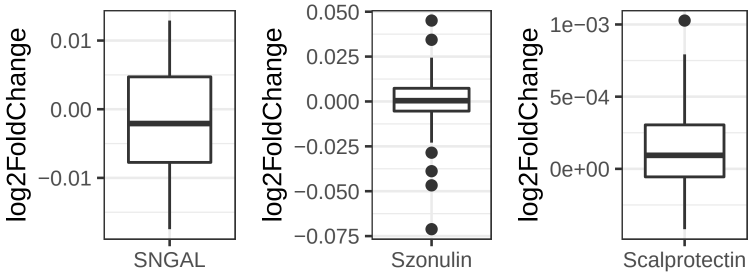

No major outliers. Export and plot the final results.

Additional file 11B

```
# Rename the group levels
inf_res_df_c$Subset <- factor(inf_res_df_c$Subset, levels = c("all", "pdc", "C", "P"))
levels(inf_res_df_c$Subset) <- c("full data",
                                 "full data, corrected for PD/C and confounders",
                                 "control only", "PD only")
# Nicer names for the Marker variable
inf_res_df_c$Marker <- sub("^S", "Stool ", inf_res_df_c$Marker)

# Export full results
write.csv(inf_res_df_c, "Outputs/add11B_ds2_inf.csv", row.names = FALSE)
```

Fig 7: markers

```
# Add line breaks to group levels
levels(inf_res_df_c$Subset) <- c("full\ndata",
                                 "full\ndata,\ncorrected\nfor PD/C and\nconfounders",
                                 "control\nonly", "PD\nonly")

# Reverse taxon list to have the alphabetical order from top to bottom
inf_res_df_c$Taxon <- factor(inf_res_df_c$Taxon,
                             levels = rev(sort(levels(inf_res_df_c$Taxon))))

# Trim to genera for plot
inf_res_df_c_gen <- subset(inf_res_df_c, Level == "Genus")

# Italic labels as an expression
da_marker_taxa <- rev(unique(subset(inf_res_df_c_gen,
                                Subset == levels(inf_res_df_c$Subset)[2])$Taxon))

da_marker_tax_labels <- parse(text = c(
  paste("italic('", gsub(".*:", "", da_marker_taxa),"')", sep = "")))

# Workaround to create only y-axis labels + legend title
```

```r
marker_y <- ggplot(subset(inf_res_df_c_gen,
                          Subset == levels(inf_res_df_c$Subset)[2]),
                   aes(x = Subset, y = Taxon, fill = log2FoldChange)) +
  geom_tile(color = "white", width = 0) +
  theme_bw(base_size = 9) +
  ggtitle(" ") +
  scale_fill_gradient2(low = "white", mid = "white", high = "white",
                       name = "log2\nfold\nchange") +
  scale_y_discrete(labels = da_marker_tax_labels) +
  theme(legend.position = "bottom",
        legend.key.size = unit(0, "mm"),
        legend.text = element_blank(),
        legend.margin = margin(r = 30, b = 10),
        axis.title = element_blank(),
        panel.grid = element_blank(),
        panel.background = element_rect(color = "white"),
        panel.border = element_rect(color = "white"),
        axis.text.x = element_text(color = "white"),
        axis.text.y = element_text(color = "black", margin = margin(r = -5, "mm")),
        axis.ticks = element_blank())

# Put together plots
da_marker_plots <- arrangeGrob(
  marker_y,
  inf_heatmap(inf_res_df_c_gen, "Stool NGAL") +
    scale_fill_gradient2(low = "gray25", mid = "white", high = "firebrick4",
                         name = "log2\nfold\nchange", breaks = c(-0.01, 0, 0.01)),
  inf_heatmap(inf_res_df_c_gen, "Stool zonulin") +
     scale_fill_gradient2(low = "gray25", mid = "white", high = "firebrick4",
                         name = "log2\nfold\nchange", breaks = c(-0.05, 0, 0.03)),
  inf_heatmap(inf_res_df_c_gen, "Stool calprotectin") +
        scale_fill_gradient2(low = "black", mid = "white", high = "firebrick4",
                         name = "log2\nfold\nchange", breaks = c(0, 0.0005, 0.0010),
                         labels = function(x) format(x, scientific = FALSE)),
  nrow = 1, widths = c(0.65, 1.5, 1.5, 1.5))

grid.arrange(da_marker_plots)
```

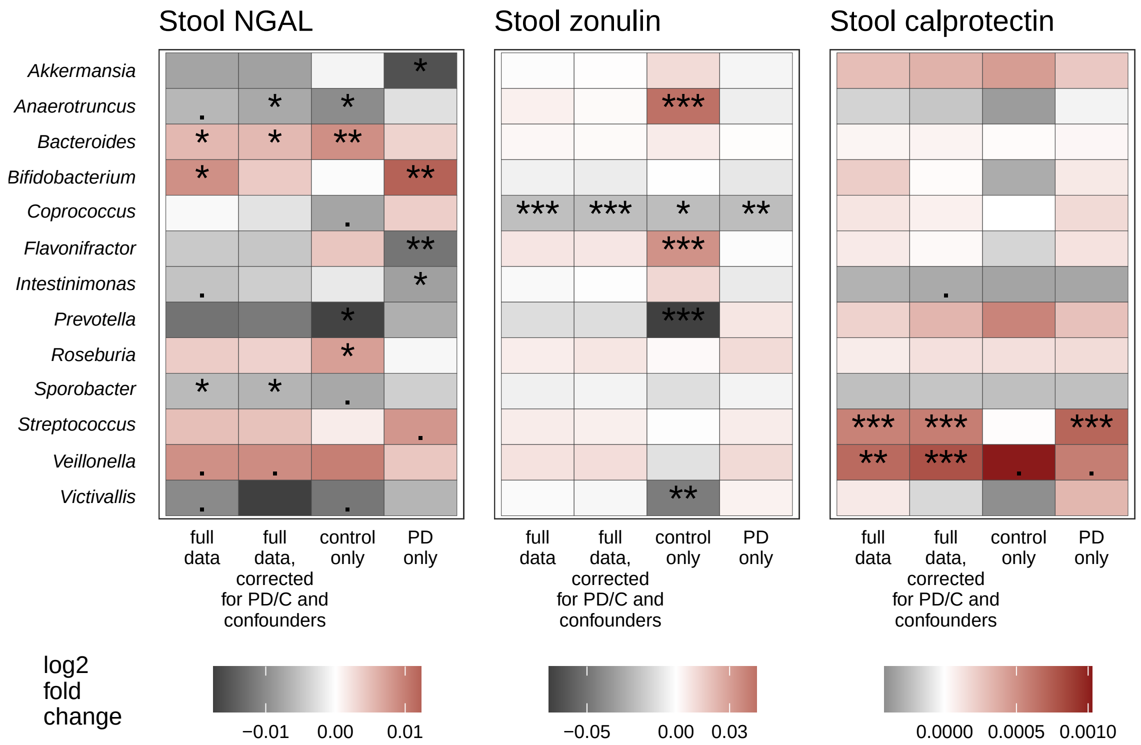

Fig 7

Combine and export the plots for differentially abundant genera for SCFAs and markers:

```r
# Blank panel for plotting
library("grid")
blankPanel <- grid.rect(gp = gpar(col = "white"), draw = FALSE)

da_plots <- arrangeGrob(da_scfa_plots,
  arrangeGrob(blankPanel, da_marker_plots, nrow = 1, widths = c(0.1, 1)),
  heights = c(length(unique(scfa_res_df_c_gen$Taxon)),
              length(unique(inf_res_df_c_gen$Taxon))))

# Export combined figure
ggsave(da_plots, filename = "Outputs/fig7_da_plots.pdf",
       width = maxwd, height = maxhi*0.9, units = "mm")
embedFonts("Outputs/fig7_da_plots.pdf")
```

Additional file 12

Separate plots for the most interesting taxa for each stool inflammatory marker:

NGAL

```r
# List of significant taxa
ngal_sigs <- as.character(unique(
  subset(inf_res_df_c_gen, Marker == "Stool NGAL" & padj < 0.05)$Taxon))

# Plot
ngal_da_scatters <- do.call(
  arrangeGrob, c(lapply(ngal_sigs, function(x)
    ra_scatter(ra_df, "SNGAL", x) +
      xlab("Stool NGAL (pg/mg)")), ncol = 3))
```

```
grid.arrange(ngal_da_scatters)
```

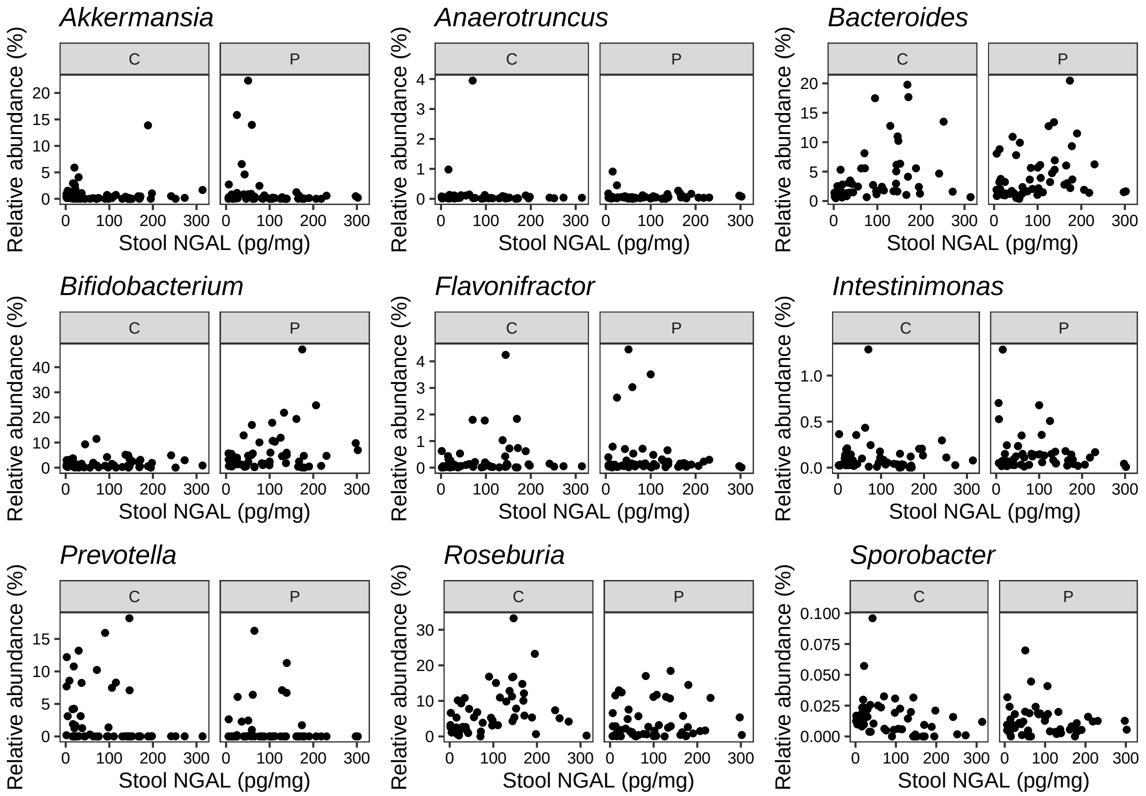

Zonulin

```
zonu_sigs <- as.character(unique(
  subset(inf_res_df_c_gen, Marker == "Stool zonulin" & padj < 0.05)$Taxon))

zonu_da_scatters <- do.call(
  arrangeGrob, c(lapply(zonu_sigs, function(x)
    ra_scatter(ra_df, "Szonulin", x) +
      xlab("Stool zonulin (pg/mg)")), ncol = 3))
grid.arrange(zonu_da_scatters)
```

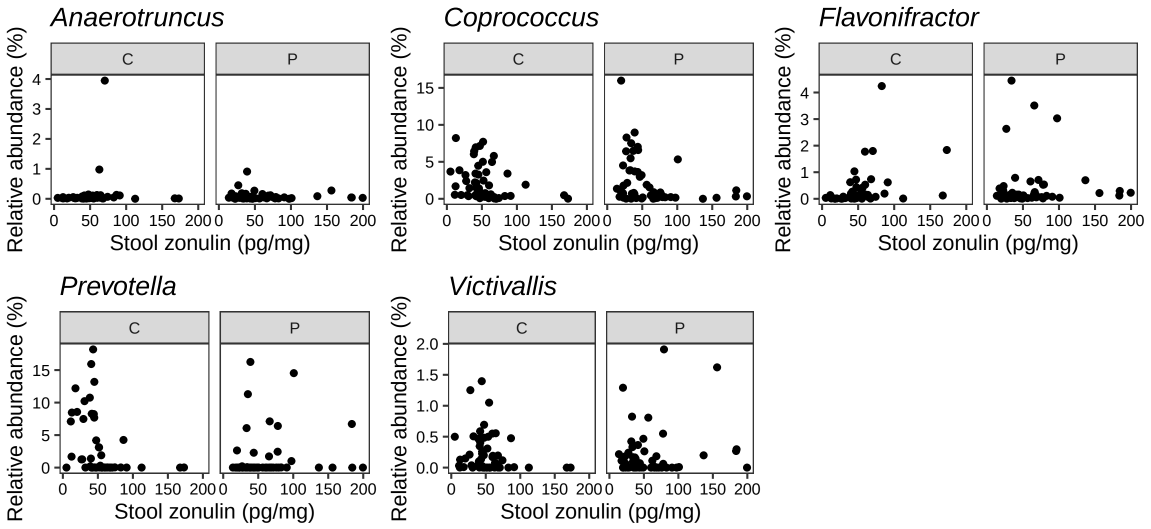

Calprotectin

```
calp_sigs <- as.character(unique(
  subset(inf_res_df_c_gen, Marker == "Stool calprotectin" & padj < 0.05)$Taxon))

calp_da_scatters <- do.call(
  arrangeGrob, c(lapply(calp_sigs, function(x)
    ra_scatter(ra_df, "Scalprotectin/1000", x) +
      xlab("Stool calprotectin (ng/mg)")), ncol = 3))
grid.arrange(calp_da_scatters)
```

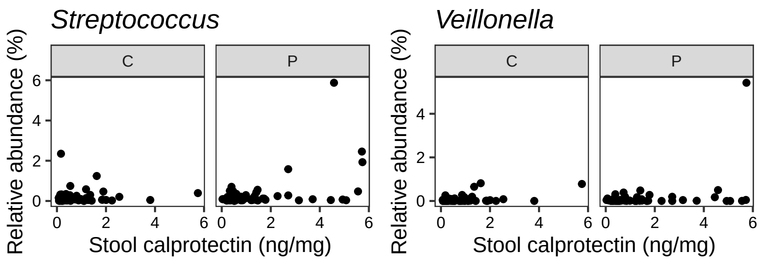

Export the figures together:

```
library("grid") # (repeated because knitr seems to forget it)

pdf("Outputs/add12_diffabund_scatters_markers.pdf",
    width = 7.5, height = 10)
grid.arrange(
  textGrob("A", x = unit(0.025, "npc"), y = unit(0.4, "npc"),
           gp = gpar(fontface = "bold", fontsize = 12)),
  ngal_da_scatters,
  textGrob("B", x = unit(0.025, "npc"), y = unit(0.4, "npc"),
           gp = gpar(fontface = "bold", fontsize = 12)),
  zonu_da_scatters,
    textGrob("C", x = unit(0.025, "npc"), y = unit(0.4, "npc"),
           gp = gpar(fontface = "bold", fontsize = 12)),
  calp_da_scatters,
  ncol = 1, heights = c(0.15, 3, 0.15, 2, 0.15, 1))
dev.off()
```

4 Revision: Summary table of key results / Additional file 13

Collect the main results of the analysis into one big summary table:

```
# Set up data frame
summary_main_vars <- sub(" tool", " ", varLabelFix(inf_scfa_vars))
names(summary_main_vars) <- inf_scfa_vars
res_summary <- data.frame(Variable = summary_main_vars)

# Basic differences in variables for PD vs C
basic_summary <- rbind(
  data.frame(
    Variable = subset(table_basic_pdc, pVal < 0.05)$var,
    Result = ifelse(
      as.numeric(sub(" .*", "", subset(table_basic_pdc, pVal < 0.05)$P)) >
        as.numeric(sub(" .*", "", subset(table_basic_pdc, pVal < 0.05)$C)),
```

```
      "PD: +", "PD: -"),
    Type = "A"),
  data.frame(
    Variable = subset(table_basic_pdc_sex, pVal_female < 0.05)$var,
    Result = ifelse(
      as.numeric(
        sub(" .*", "", subset(table_basic_pdc_sex, pVal_female < 0.05)$P_female)) >
        as.numeric(
          sub(" .*", "", subset(table_basic_pdc_sex, pVal_female < 0.05)$C_female)),
      "PD: +", "PD: -"),
    Type = "F"),
    data.frame(
    Variable = subset(table_basic_pdc_sex, pVal_male < 0.05)$var,
    Result = ifelse(
      as.numeric(
        sub(" .*", "", subset(table_basic_pdc_sex, pVal_male < 0.05)$P_male)) >
        as.numeric(sub(" .*", "", subset(table_basic_pdc_sex, pVal_male < 0.05)$C_male)),
      "PD: +", "PD: -"),
    Type = "M"))
basic_summary <- aggregate(list(Type = basic_summary$Type, Result = basic_summary$Result),
                           list(Variable = basic_summary$Variable),
                           paste)
basic_summary$Concentration_PD_vs_C <- paste0(
  unlist(lapply(basic_summary$Result, unique)), " (",
  unlist(lapply(basic_summary$Type, paste, collapse = ", ")), ")")
res_summary <- merge(res_summary,
                     basic_summary[,c("Variable", "Concentration_PD_vs_C")],
                     all = TRUE)

# Significant correlations with clinical variables
clin_cors_P <- meta_cors_P[,1:4]
colnames(clin_cors_P) <- c("Confounder", "Subset", "Variable", "p")
colnames(meta_cors_r) <- c("Confounder", "Subset", "Variable", "r")
clin_summary <- merge(clin_cors_P, meta_cors_r)
clin_summary <- subset(clin_summary, p < 0.05 & Variable != "Group")
clin_summary$Direction <- ifelse(clin_summary$r > 0, "cor", "inv")
clin_summary$Subset <- sub("PD patients", "P",
                           sub("Control.*", "C",
                               sub("All.*", "A", clin_summary$Subset)))
clin_summary <- aggregate(Subset ~ Confounder + Variable + Direction, clin_summary,
                          function(x) paste(sort(x), collapse = ", "))
# Simplifying some confounder names
clin_summary$Confounder <- capitalize(sub("History ", "",
                                          sub("Medication ", "",
                                              clin_summary$Confounder)))
clin_summary$Confounder <- mgsub(clin_summary$Confounder,
                                 c("stool collection", "motor symptoms",
                                   "blocker", "Calculated t", "Diuretic",
                                   "inhibitor", " IBS criteria fulfilled",
                                   "Rome III 9-15 sum score",
                                   "Statin", "TIA ischemic stroke", " total"),
                                 c("sampling", "motor",
                                   "blockers", "T", "Diuretics",
```

```
                                   "inhibitors", ": IBS+", "Rome III: 9-15",
                                   "Statins", "TIA\\/stroke", ""))
clin_summary$Res <- paste0(clin_summary$Confounder,
                           " (", clin_summary$Subset, ")")
# Fix target variable names
clin_summary$Variable <- summary_main_vars[as.character(clin_summary$Variable)]


# Separate for correlations / inverse correlations
clin_cor_summary <- aggregate(
  list(Clin_cor = subset(clin_summary, Direction == "cor")$Res),
  list(Variable = subset(clin_summary, Direction == "cor")$Variable),
  paste, collapse = ", ")

clin_inv_summary <- aggregate(
  list(Clin_invcor = subset(clin_summary, Direction == "inv")$Res),
  list(Variable = subset(clin_summary, Direction == "inv")$Variable),
  paste, collapse = ", ")

# Add to main table
res_summary <- merge(res_summary,
                     merge(clin_cor_summary, clin_inv_summary, all = TRUE),
                     all = TRUE)

# Alpha diversity
# (significant for either of the two indices, not specified separately)
adiv_summary <- unique(data.frame(
    Variable = subset(adiv_cors_df, P < 0.05)$Var,
    Result = ifelse(subset(adiv_cors_df, P < 0.05)$Cor > 0,
                    "yes; +", "yes; -"),
    Notes = sub("\n", " ", subset(adiv_cors_df, P < 0.05)$Set)))
adiv_summary <- aggregate(list(Notes = adiv_summary$Notes, Result = adiv_summary$Result),
                          list(Variable = adiv_summary$Variable),
                          paste)
adiv_summary$Alpha_diversity <- paste0(
  unlist(lapply(adiv_summary$Result, unique)), " (",
  unlist(lapply(adiv_summary$Notes, paste, collapse = ", ")), ")")
adiv_summary$Alpha_diversity  <- sub("Full data", "A",
                                     sub("PD only", "P",
                                         sub("Controls only", "C",
                                             adiv_summary$Alpha_diversity)))
res_summary <- merge(res_summary,
                     adiv_summary[,c("Variable", "Alpha_diversity")],
                     all = TRUE)

# Beta diversity
bdiv_summary <- data.frame(
  Variable = subset(melt(bdiv_combo), value < 0.05)$Variable,
  Subset = capitalize(
    sub("\\)", "", sub("p \\(", "",
             subset(melt(bdiv_combo), value < 0.05)$variable))))
bdiv_summary$Subset <- sub("Full data", "A",
                          sub("PD only", "P",
```

```r
                              sub("Control only", "C",
                                  bdiv_summary$Subset)))
bdiv_summary <- aggregate(Subset ~ Variable,
                          bdiv_summary, paste, collapse = ", ")
# Since all of these are significant with full data or with corrections,
# drop the mentions of corrections
bdiv_summary$Beta_diversity <- paste0("yes (",
                                      sub(", PD/C corrected, PD/C \\+ confounder corrected",
                                          "", bdiv_summary$Subset), ")")
res_summary <- merge(res_summary,
                     bdiv_summary[,c("Variable", "Beta_diversity")],
                     all = TRUE)

# Enterotypes
# (considering unadjusted p-values as everywhere else)
kw_et_combo_unadj <- kw_et_combo[,-grep("pAdj", colnames(kw_et_combo))]
et_summary <- data.frame(
  Variable = subset(melt(kw_et_combo_unadj), value < 0.05)$Variable,
  Notes = sub("pVal_PD", "P",
              sub("pVal_C", "C",
                  sub("pVal_all", "A",
                      subset(melt(kw_et_combo_unadj),
                             value < 0.05)$variable))))
et_summary <- aggregate(Notes ~ Variable, et_summary, paste, collapse = ", ")
et_summary$Enterotypes <- paste0("yes (",
                                 et_summary$Notes, ")")
res_summary <- merge(res_summary,
                     et_summary[, c("Variable", "Enterotypes")],
                     all = TRUE)

# Diffabunds
# (Due to the table getting too complex otherwise; trimmed to
# those taxa that were significant in > 2 comparisons and the
# subset information left out)

colnames(scfa_res_df_c)[1] <- "Variable"
colnames(inf_res_df_c)[1] <- "Variable"
da_combo_df_gen <- rbind(
  subset(scfa_res_df_c, Level == "Genus"),
  subset(inf_res_df_c, Level == "Genus"))
da_combo_df_gen$Subset <- sub("control only", "C",
                              sub("PD only", "P",
                                  sub("full data", "A\\*",
                                      sub(".*confounders", "A",
                              gsub("\n", " ", da_combo_df_gen$Subset)))))
da_summary <- data.frame(
  subset(da_combo_df_gen, padj < 0.05)[,c("Variable", "Taxon", "Subset")],
  Direction = ifelse(subset(da_combo_df_gen, padj < 0.05)$log2FoldChange > 0,
                     "inc", "dec"))
da_summary <- aggregate(Subset ~ Taxon + Variable + Direction, da_summary,
                        function(x) paste(sort(x), collapse = ", "))
da_summary$Subset <- sub("A, A\\*", "A", da_summary$Subset)
da_summary$Res <- paste0(da_summary$Taxon, " (", da_summary$Subset, ")")
```

```
da_up_summary <- aggregate(list(DA_Up = subset(da_summary, Direction == "inc")$Res),
                           list(Variable = subset(da_summary, Direction == "inc")$Variable),
                           paste, collapse = ", ")
da_down_summary <- aggregate(list(DA_Down = subset(da_summary, Direction == "dec")$Res),
                           list(Variable = subset(da_summary, Direction == "dec")$Variable),
                           paste, collapse = ", ")

res_summary <- merge(res_summary,
                     merge(da_up_summary, da_down_summary, all = TRUE),
                     all = TRUE)

# Trim variables with nothing significant or
# only clinical variable correlations and nothing else
res_summary <- subset(res_summary, rowSums(!is.na(res_summary[c(2, 5:9)])) > 0)

# Trim variables that ended up in the PCs
res_summary <- subset(res_summary, Variable %in% summary_main_vars)

# Fix NAs for variables that weren't tested with DS2
res_summary[!(res_summary$Variable %in% unique(da_summary$Variable)),
            "DA_Up"] <- "not tested"
res_summary[!(res_summary$Variable %in% unique(da_summary$Variable)),
            "DA_Down"] <- "not tested"

# Reorder variables
res_summary <- res_summary[na.omit(match(summary_main_vars,
                                         res_summary$Variable)),]

# Export
write.csv(res_summary, "Outputs/add13_summary_table.csv", row.names = FALSE,
          qmethod = "escape")
```

5 Additional analyses

5.1 Sample storage time

An additional test added based on reviewer feedback from an early submission: effects of stool sample storage time on the SCFA measurements.

```
# Read in data
date_data <- read.csv("Inputs/pdfu_sampling_dates.csv", row.names = 1)

# Does this have all the samples?
setdiff(rownames(pdcy_meta), rownames(date_data))
```

```
## [1] "C0044" "P0083"
```

```
# two are missing, but unfortunately this data is not easily available at
# present, so these two are excluded

# Match dates to actual data that is included in the analyses
date_data <- date_data[rownames(pdcy_meta),]
date_data <- subset(date_data, !is.na(GROUP))
```

```r
# Fix dates to the right format
date_colnames <- colnames(date_data)[4:ncol(date_data)]
for(i in date_colnames){
  date_data[[i]] <- as.Date(date_data[[i]], "%d/%m/%y")
}

# Calculate differences in dates
date_data$followup_storage_days <- date_data$appointment_followup -
  date_data$stool_date_followup

# There are patients whose appointment is marked as having been before
# stool sampling; these are cases where there was some kind of a problem
# with the original stool sample.
table(date_data$followup_storage_days)
```

```
##
## -77 -54 -15 -14  -5  -1   0   1   2   3   4
##   1   1   1   1   2   2  12  50  34   4   1
```

```r
# Again, since getting this information would be challenging at present,
# these are also treated as missing data.
date_data$followup_storage_days[date_data$followup_storage_days < 0] <- NA

# Make a factor and a numeric version for easier handling
date_data$Storage_days_cat <- factor(date_data$followup_storage_days)
date_data$Storage_days_num <- as.numeric(date_data$followup_storage_days)

# Merge with main metadata
date_data$Subject <- rownames(date_data)
pdcy_storage <- merge(pdcy_meta, date_data, by = "Subject")

# Drop the NA subjects
pdcy_storage <- subset(pdcy_storage, !is.na(followup_storage_days))

# Plot SCFA concentrations by storage time
do.call("grid.arrange", lapply(scfa_vars[1:4], function(x)
  ggplot(pdcy_storage, aes_string(x = "Storage_days_cat", y = x)) +
    geom_boxplot(width = 0.3, outlier.shape = NA) +
    geom_jitter(width = 0.2, alpha = 0.75, size = 0.5) +
  theme_bw(base_size = 8) +
  theme(panel.grid.minor = element_blank(),
        panel.grid.major.x = element_blank())))
```

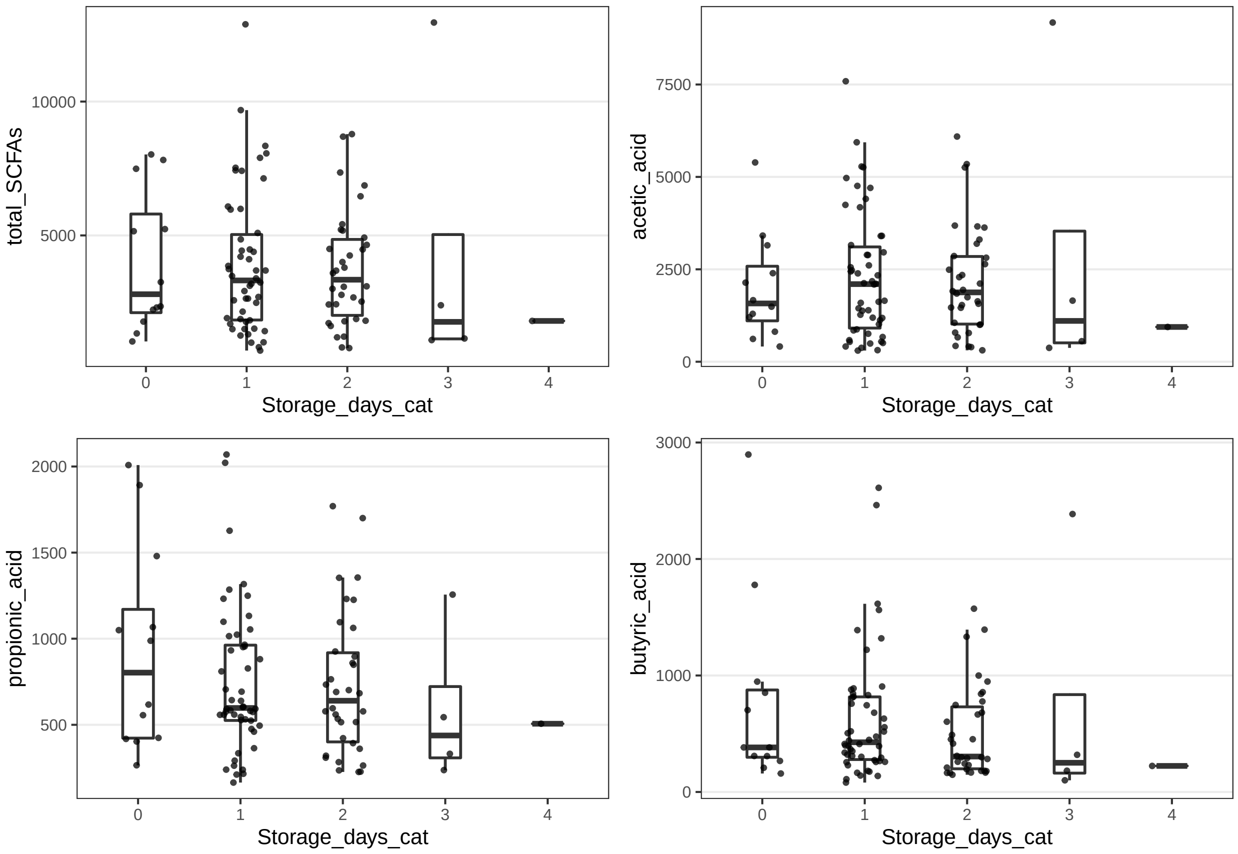


```r
# Test for statistically significant differences

# As categorical
sapply(scfa_vars[1:4], function(x)
  kruskal.test(pdcy_storage[,x], pdcy_storage$Storage_days_cat)$p.value)
```

```
##    total_SCFAs    acetic_acid propionic_acid   butyric_acid
##      0.8049453      0.8749020      0.6911163      0.5408469
```

```r
# As numeric
sapply(scfa_vars[1:4], function(x)
  cor.test(x = pdcy_storage[,x], y = pdcy_storage$Storage_days_num)$p.value)
```

```
##    total_SCFAs    acetic_acid propionic_acid   butyric_acid
##      0.6784743      0.8669362      0.1468434      0.2295269
```

On visual inspection, it does seem that samples stored for 3 or 4 days have lower median SCFA concentrations, but there aren't many of such samples (5/101 included in this comparison).

6 Session info

List all R packages used for these analyses:

```r
devtools::session_info()
```

```
## - Session info ---------------------------------------------------------------
##  setting  value
##  version  R version 4.0.2 (2020-06-22)
##  os       macOS Catalina 10.15.7
```

```
##  system   x86_64, darwin17.0
##  ui       X11
##  language (EN)
##  collate  en_US.UTF-8
##  ctype    en_US.UTF-8
##  tz       Europe/Luxembourg
##  date     2020-12-08
##
## - Packages -------------------------------------------------------------------
##  package              * version  date       lib source
##  abind                  1.4-5    2016-07-21 [1] CRAN (R 4.0.0)
##  ade4                   1.7-15   2020-02-13 [1] CRAN (R 4.0.0)
##  annotate               1.66.0   2020-04-28 [1] Bioconductor
##  AnnotationDbi          1.50.3   2020-07-25 [1] Bioconductor
##  ape                    5.4-1    2020-08-13 [1] CRAN (R 4.0.2)
##  assertthat             0.2.1    2019-03-21 [1] CRAN (R 4.0.0)
##  backports              1.1.10   2020-09-15 [1] CRAN (R 4.0.2)
##  base64enc              0.1-3    2015-07-28 [1] CRAN (R 4.0.0)
##  Biobase              * 2.48.0   2020-04-27 [1] Bioconductor
##  BiocGenerics         * 0.34.0   2020-04-27 [1] Bioconductor
##  BiocParallel           1.22.0   2020-04-27 [1] Bioconductor
##  biomformat             1.16.0   2020-04-27 [1] Bioconductor
##  Biostrings             2.56.0   2020-04-27 [1] Bioconductor
##  bit                    4.0.4    2020-08-04 [1] CRAN (R 4.0.2)
##  bit64                  4.0.5    2020-08-30 [1] CRAN (R 4.0.2)
##  bitops                 1.0-6    2013-08-17 [1] CRAN (R 4.0.0)
##  blob                   1.2.1    2020-01-20 [1] CRAN (R 4.0.0)
##  broom                  0.7.0    2020-07-09 [1] CRAN (R 4.0.2)
##  callr                  3.4.4    2020-09-07 [1] CRAN (R 4.0.2)
##  car                    3.0-9    2020-08-11 [1] CRAN (R 4.0.2)
##  carData                3.0-4    2020-05-22 [1] CRAN (R 4.0.0)
##  cellranger             1.1.0    2016-07-27 [1] CRAN (R 4.0.0)
##  checkmate              2.0.0    2020-02-06 [1] CRAN (R 4.0.0)
##  cli                    2.0.2    2020-02-28 [1] CRAN (R 4.0.0)
##  cluster                2.1.0    2019-06-19 [1] CRAN (R 4.0.2)
##  codetools              0.2-16   2018-12-24 [1] CRAN (R 4.0.2)
##  colorspace             1.4-1    2019-03-18 [1] CRAN (R 4.0.0)
##  commonmark             1.7      2018-12-01 [1] CRAN (R 4.0.0)
##  crayon                 1.3.4    2017-09-16 [1] CRAN (R 4.0.0)
##  curl                   4.3      2019-12-02 [1] CRAN (R 4.0.0)
##  data.table             1.13.0   2020-07-24 [1] CRAN (R 4.0.2)
##  DBI                    1.1.0    2019-12-15 [1] CRAN (R 4.0.0)
##  DelayedArray         * 0.14.1   2020-07-14 [1] Bioconductor
##  desc                   1.2.0    2018-05-01 [1] CRAN (R 4.0.0)
##  DESeq2               * 1.28.1   2020-05-12 [1] Bioconductor
##  devtools               2.3.2    2020-09-18 [1] CRAN (R 4.0.2)
##  digest                 0.6.25   2020-02-23 [1] CRAN (R 4.0.0)
##  dplyr                  1.0.2    2020-08-18 [1] CRAN (R 4.0.2)
##  ellipsis               0.3.1    2020-05-15 [1] CRAN (R 4.0.0)
##  evaluate               0.14     2019-05-28 [1] CRAN (R 4.0.0)
##  factoextra           * 1.0.7    2020-04-01 [1] CRAN (R 4.0.0)
##  fansi                  0.4.1    2020-01-08 [1] CRAN (R 4.0.0)
##  farver                 2.0.3    2020-01-16 [1] CRAN (R 4.0.0)
##  forcats                0.5.0    2020-03-01 [1] CRAN (R 4.0.0)
```

```
##  foreach                1.5.0    2020-03-30 [1] CRAN (R 4.0.0)
##  foreign                0.8-80   2020-05-24 [1] CRAN (R 4.0.2)
##  Formula              * 1.2-3    2018-05-03 [1] CRAN (R 4.0.0)
##  fs                     1.5.0    2020-07-31 [1] CRAN (R 4.0.2)
##  genefilter             1.70.0   2020-04-27 [1] Bioconductor
##  geneplotter            1.66.0   2020-04-27 [1] Bioconductor
##  generics               0.0.2    2018-11-29 [1] CRAN (R 4.0.0)
##  GenomeInfoDb         * 1.24.2   2020-06-15 [1] Bioconductor
##  GenomeInfoDbData       1.2.3    2020-06-25 [1] Bioconductor
##  GenomicRanges        * 1.40.0   2020-04-27 [1] Bioconductor
##  GGally               * 2.0.0    2020-06-06 [1] CRAN (R 4.0.2)
##  ggcorrplot           * 0.1.3    2019-05-19 [1] CRAN (R 4.0.0)
##  ggfortify            * 0.4.10   2020-04-26 [1] CRAN (R 4.0.0)
##  ggplot2              * 3.3.2    2020-06-19 [1] CRAN (R 4.0.2)
##  ggpubr                 0.4.0    2020-06-27 [1] CRAN (R 4.0.2)
##  ggrepel                0.8.2    2020-03-08 [1] CRAN (R 4.0.0)
##  ggsignif               0.6.0    2019-08-08 [1] CRAN (R 4.0.0)
##  glue                   1.4.2    2020-08-27 [1] CRAN (R 4.0.2)
##  gridExtra            * 2.3      2017-09-09 [1] CRAN (R 4.0.0)
##  gtable                 0.3.0    2019-03-25 [1] CRAN (R 4.0.0)
##  gtools               * 3.8.2    2020-03-31 [1] CRAN (R 4.0.0)
##  haven                  2.3.1    2020-06-01 [1] CRAN (R 4.0.0)
##  Hmisc                * 4.4-1    2020-08-10 [1] CRAN (R 4.0.2)
##  hms                    0.5.3    2020-01-08 [1] CRAN (R 4.0.0)
##  htmlTable              2.1.0    2020-09-16 [1] CRAN (R 4.0.2)
##  htmltools              0.5.0    2020-06-16 [1] CRAN (R 4.0.0)
##  htmlwidgets            1.5.1    2019-10-08 [1] CRAN (R 4.0.0)
##  httr                   1.4.2    2020-07-20 [1] CRAN (R 4.0.2)
##  huxtable             * 5.1.0    2020-09-18 [1] CRAN (R 4.0.2)
##  igraph                 1.2.5    2020-03-19 [1] CRAN (R 4.0.0)
##  IRanges              * 2.22.2   2020-05-21 [1] Bioconductor
##  iterators              1.0.12   2019-07-26 [1] CRAN (R 4.0.0)
##  jpeg                   0.1-8.1  2019-10-24 [1] CRAN (R 4.0.0)
##  jsonlite               1.7.1    2020-09-07 [1] CRAN (R 4.0.2)
##  kableExtra           * 1.2.1    2020-08-27 [1] CRAN (R 4.0.2)
##  knitr                * 1.30     2020-09-22 [1] CRAN (R 4.0.2)
##  labeling               0.3      2014-08-23 [1] CRAN (R 4.0.0)
##  lattice              * 0.20-41  2020-04-02 [1] CRAN (R 4.0.2)
##  latticeExtra           0.6-29   2019-12-19 [1] CRAN (R 4.0.0)
##  lifecycle              0.2.0    2020-03-06 [1] CRAN (R 4.0.0)
##  locfit                 1.5-9.4  2020-03-25 [1] CRAN (R 4.0.0)
##  magrittr               1.5      2014-11-22 [1] CRAN (R 4.0.0)
##  MASS                   7.3-53   2020-09-09 [1] CRAN (R 4.0.2)
##  Matrix                 1.2-18   2019-11-27 [1] CRAN (R 4.0.2)
##  matrixStats          * 0.56.0   2020-03-13 [1] CRAN (R 4.0.0)
##  memoise                1.1.0    2017-04-21 [1] CRAN (R 4.0.0)
##  mgcv                   1.8-33   2020-08-27 [1] CRAN (R 4.0.2)
##  mgsub                * 1.7.2    2020-07-22 [1] CRAN (R 4.0.2)
##  multtest               2.44.0   2020-04-27 [1] Bioconductor
##  munsell                0.5.0    2018-06-12 [1] CRAN (R 4.0.0)
##  nlme                   3.1-149  2020-08-23 [1] CRAN (R 4.0.2)
##  nnet                   7.3-14   2020-04-26 [1] CRAN (R 4.0.2)
##  openxlsx               4.2.2    2020-09-17 [1] CRAN (R 4.0.2)
##  permute              * 0.9-5    2019-03-12 [1] CRAN (R 4.0.0)
```

```
##  phyloseq             * 1.32.0   2020-04-27 [1] Bioconductor
##  pillar                 1.4.6    2020-07-10 [1] CRAN (R 4.0.2)
##  pkgbuild               1.1.0    2020-07-13 [1] CRAN (R 4.0.2)
##  pkgconfig              2.0.3    2019-09-22 [1] CRAN (R 4.0.0)
##  pkgload                1.1.0    2020-05-29 [1] CRAN (R 4.0.0)
##  plyr                   1.8.6    2020-03-03 [1] CRAN (R 4.0.0)
##  png                    0.1-7    2013-12-03 [1] CRAN (R 4.0.0)
##  prettyunits            1.1.1    2020-01-24 [1] CRAN (R 4.0.0)
##  processx               3.4.4    2020-09-03 [1] CRAN (R 4.0.2)
##  ps                     1.3.4    2020-08-11 [1] CRAN (R 4.0.2)
##  purrr                  0.3.4    2020-04-17 [1] CRAN (R 4.0.0)
##  R6                     2.4.1    2019-11-12 [1] CRAN (R 4.0.0)
##  RColorBrewer           1.1-2    2014-12-07 [1] CRAN (R 4.0.0)
##  Rcpp                   1.0.5    2020-07-06 [1] CRAN (R 4.0.2)
##  RCurl                  1.98-1.2 2020-04-18 [1] CRAN (R 4.0.0)
##  readxl                 1.3.1    2019-03-13 [1] CRAN (R 4.0.0)
##  remotes                2.2.0    2020-07-21 [1] CRAN (R 4.0.2)
##  reshape                0.8.8    2018-10-23 [1] CRAN (R 4.0.2)
##  reshape2             * 1.4.4    2020-04-09 [1] CRAN (R 4.0.0)
##  rhdf5                  2.32.2   2020-07-03 [1] Bioconductor
##  Rhdf5lib               1.10.1   2020-07-09 [1] Bioconductor
##  rio                    0.5.16   2018-11-26 [1] CRAN (R 4.0.0)
##  rlang                  0.4.7    2020-07-09 [1] CRAN (R 4.0.2)
##  rmarkdown              2.3      2020-06-18 [1] CRAN (R 4.0.0)
##  rpart                  4.1-15   2019-04-12 [1] CRAN (R 4.0.2)
##  rprojroot              1.3-2    2018-01-03 [1] CRAN (R 4.0.0)
##  RSQLite                2.2.0    2020-01-07 [1] CRAN (R 4.0.0)
##  rstatix                0.6.0    2020-06-18 [1] CRAN (R 4.0.2)
##  rstudioapi             0.11     2020-02-07 [1] CRAN (R 4.0.0)
##  rvest                  0.3.6    2020-07-25 [1] CRAN (R 4.0.2)
##  S4Vectors            * 0.26.1   2020-05-16 [1] Bioconductor
##  scales                 1.1.1    2020-05-11 [1] CRAN (R 4.0.0)
##  sessioninfo            1.1.1    2018-11-05 [1] CRAN (R 4.0.0)
##  stringi                1.5.3    2020-09-09 [1] CRAN (R 4.0.2)
##  stringr                1.4.0    2019-02-10 [1] CRAN (R 4.0.0)
##  SummarizedExperiment * 1.18.2   2020-07-14 [1] Bioconductor
##  survival             * 3.2-3    2020-06-13 [1] CRAN (R 4.0.0)
##  testthat               2.3.2    2020-03-02 [1] CRAN (R 4.0.0)
##  tibble                 3.0.3    2020-07-10 [1] CRAN (R 4.0.2)
##  tidyr                  1.1.2    2020-08-27 [1] CRAN (R 4.0.2)
##  tidyselect             1.1.0    2020-05-11 [1] CRAN (R 4.0.0)
##  usethis                1.6.3    2020-09-17 [1] CRAN (R 4.0.2)
##  vctrs                  0.3.4    2020-08-29 [1] CRAN (R 4.0.2)
##  vegan                * 2.5-6    2019-09-01 [1] CRAN (R 4.0.2)
##  viridis              * 0.5.1    2018-03-29 [1] CRAN (R 4.0.0)
##  viridisLite          * 0.3.0    2018-02-01 [1] CRAN (R 4.0.0)
##  webshot                0.5.2    2019-11-22 [1] CRAN (R 4.0.0)
##  withr                  2.3.0    2020-09-22 [1] CRAN (R 4.0.2)
##  xfun                   0.17     2020-09-09 [1] CRAN (R 4.0.2)
##  XML                    3.99-0.5 2020-07-23 [1] CRAN (R 4.0.2)
##  xml2                   1.3.2    2020-04-23 [1] CRAN (R 4.0.0)
##  xtable                 1.8-4    2019-04-21 [1] CRAN (R 4.0.0)
##  XVector                0.28.0   2020-04-27 [1] Bioconductor
##  yaml                   2.2.1    2020-02-01 [1] CRAN (R 4.0.0)
```

```
##  zip                     2.1.1    2020-08-27 [1] CRAN (R 4.0.2)
##  zlibbioc                1.34.0   2020-04-27 [1] Bioconductor
##
## [1] /Library/Frameworks/R.framework/Versions/4.0/Resources/library
```
