# Supplementary figures and images for: Relationships of gut microbiota, short-chain fatty acids, inflammation, and the gut barrier in Parkinson’s disease
Source: Mol Neurodegener. 2021 Feb 8;16:6. doi: 10.1186/s13024-021-00427-6 (PMC7869249; doi:10.1186/s13024-021-00427-6)

**A**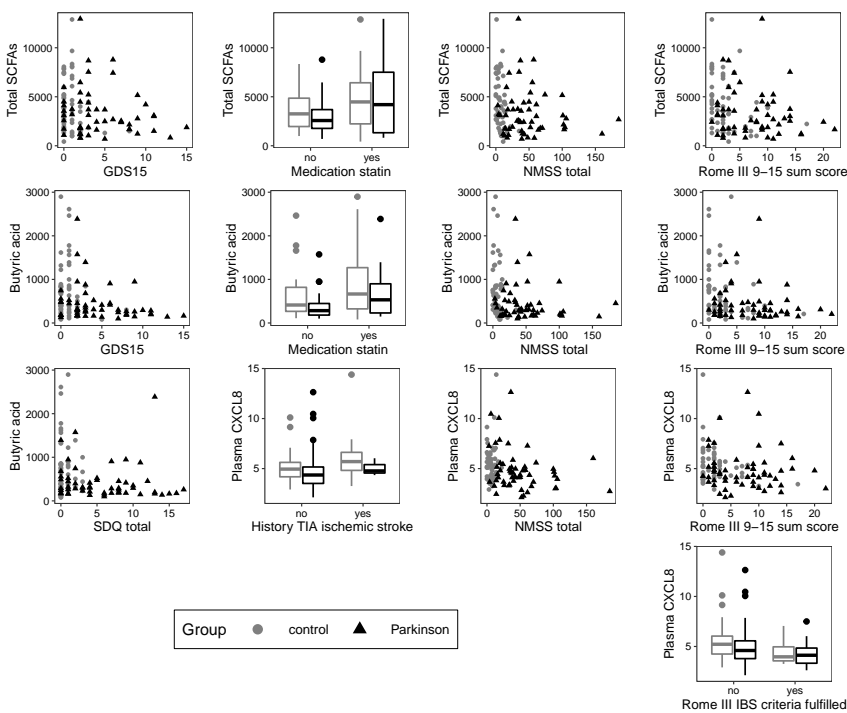**B**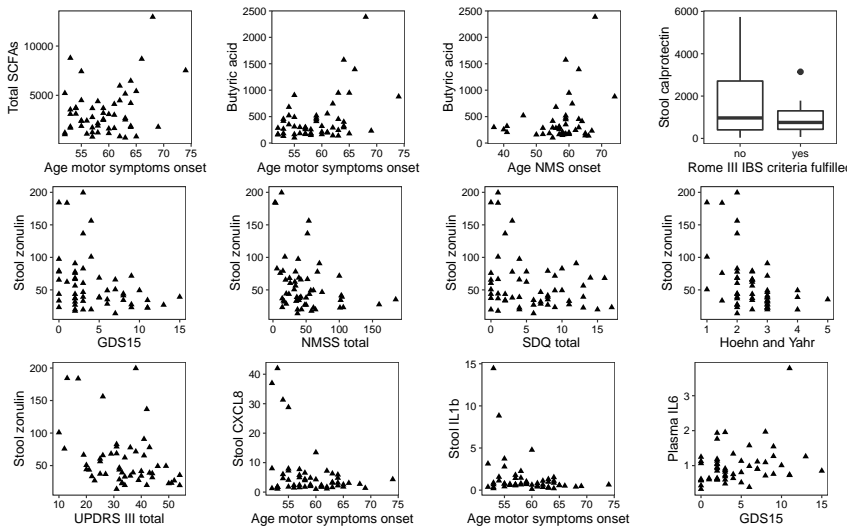

Supplement: Supplementary file 4 — Additional file 4: Scatterplots for significantly correlated clinical variables and SCFAs or inflammatory or permeability markers. Scatterplots visualizing relationships of significantly (p< 0.05, Pearson) correlated analytes and clinical variables A) measured in all subjects and B) measured only in PD patients. [file 13024_2021_427_MOESM4_ESM.pdf]

### Acetic acid

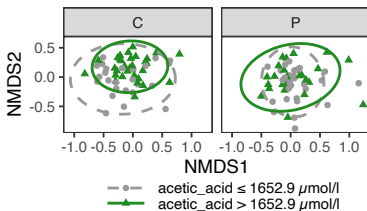

### Butyric acid

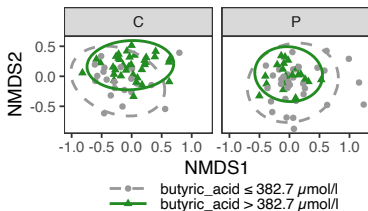

### Total SCFAs

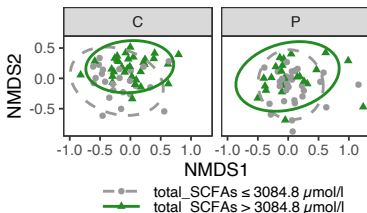

### Stool NGAL

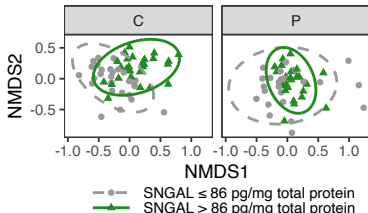

### Stool IL2

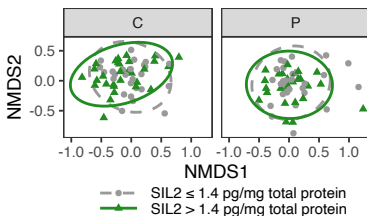

### Stool zonulin

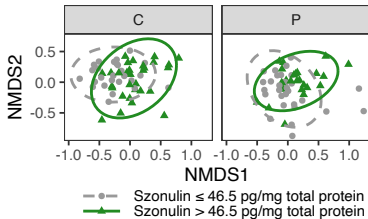

Supplement: Supplementary file 8 — Additional file 8: NMDS ordination plots for beta diversity, PD/control status, and select analytes. NMDS ordination plots for the stool markers and SCFAs associated with the most notable beta diversity difference. Variables were split into two categories by median. [file 13024_2021_427_MOESM8_ESM.pdf]

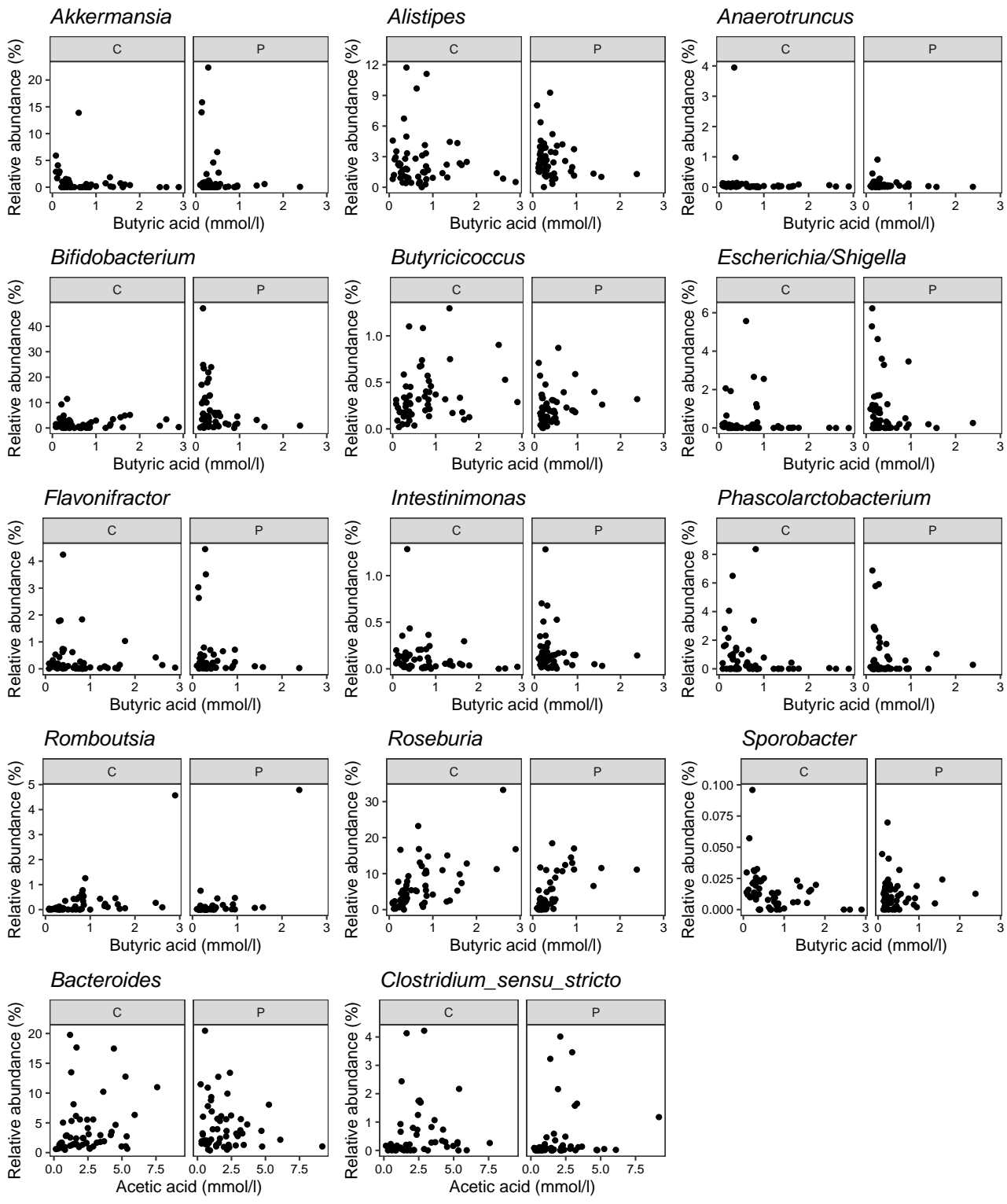

Supplement: Supplementary file 10 — Additional file 10: Scatterplots of genera relative abundances by SCFA levels. Scatterplots visualizing significant (p< 0.05, Pearson correlation) relationships between SCFAs and relative abundance of most differentially abundant bacterial genera as determined by differential expression analysis for sequence count data. [file 13024_2021_427_MOESM10_ESM.pdf]

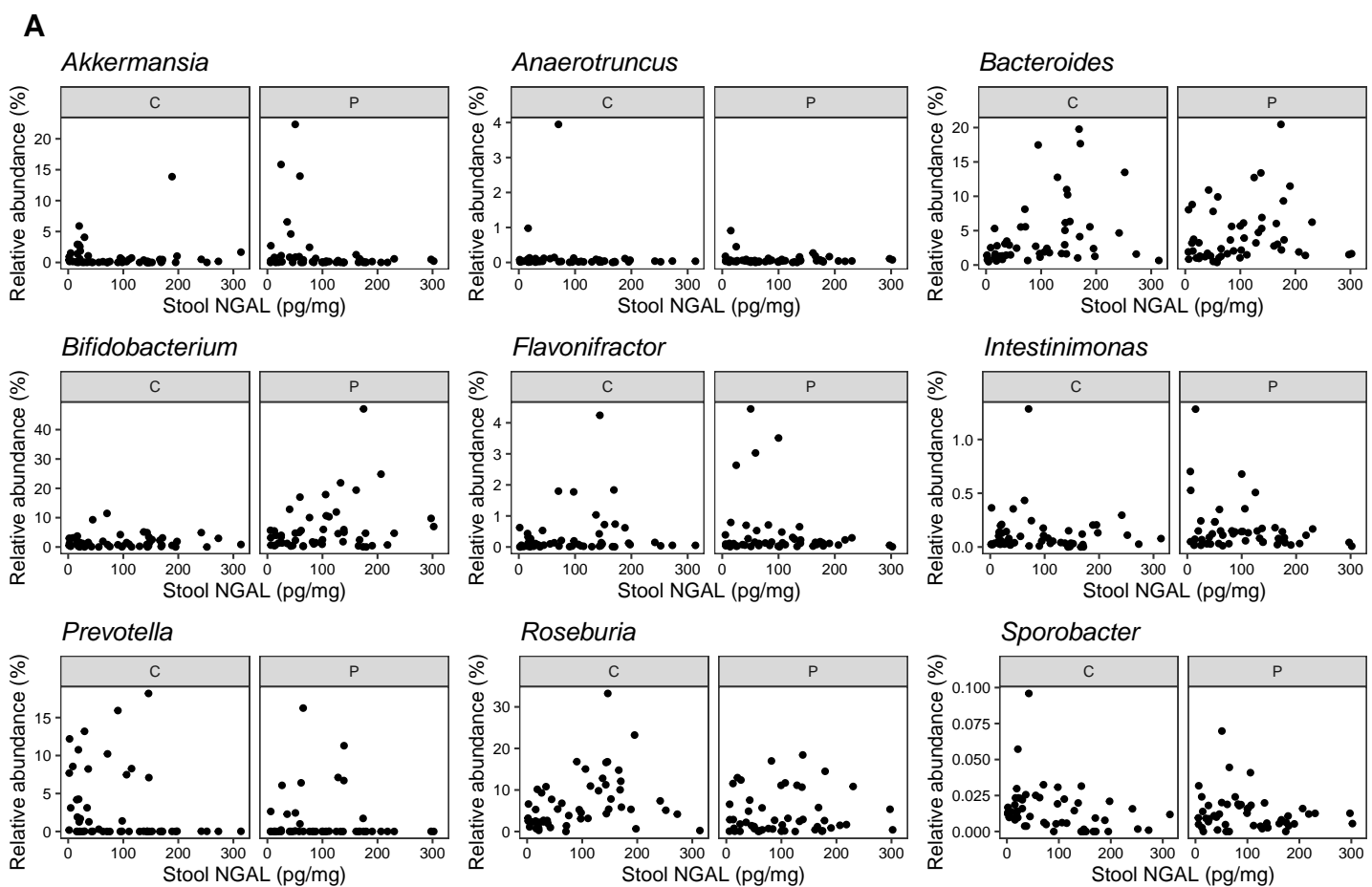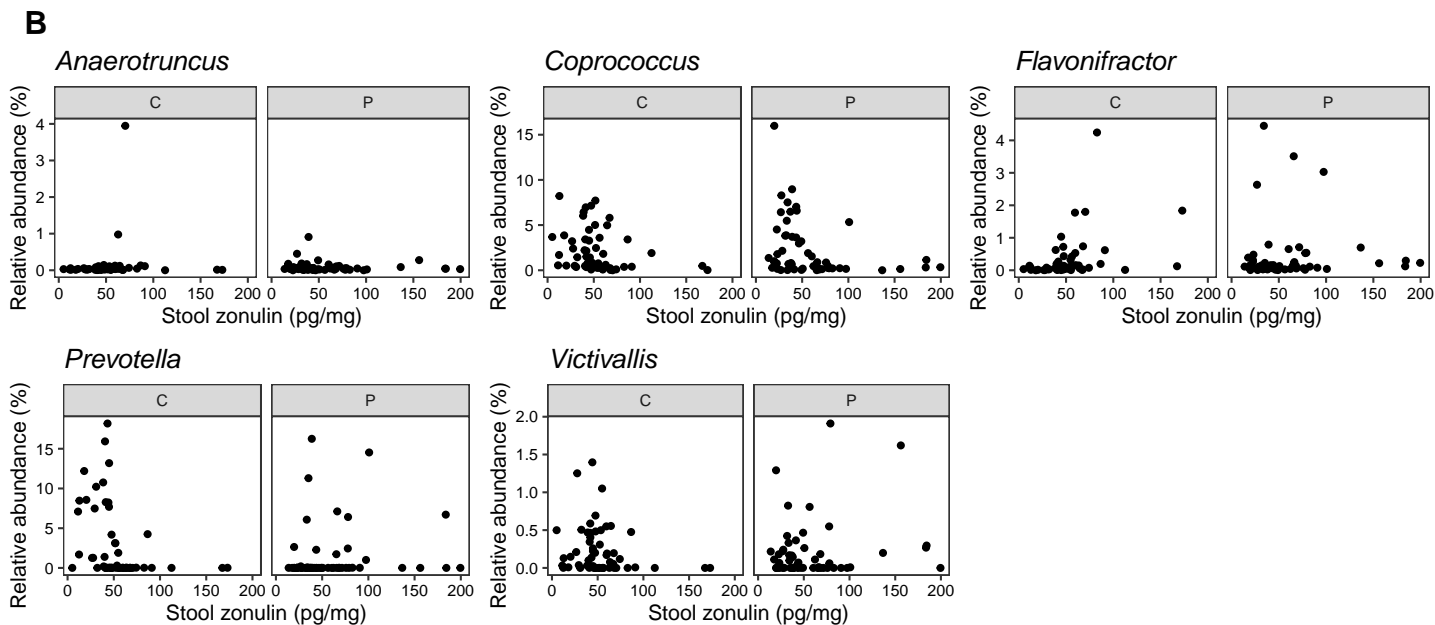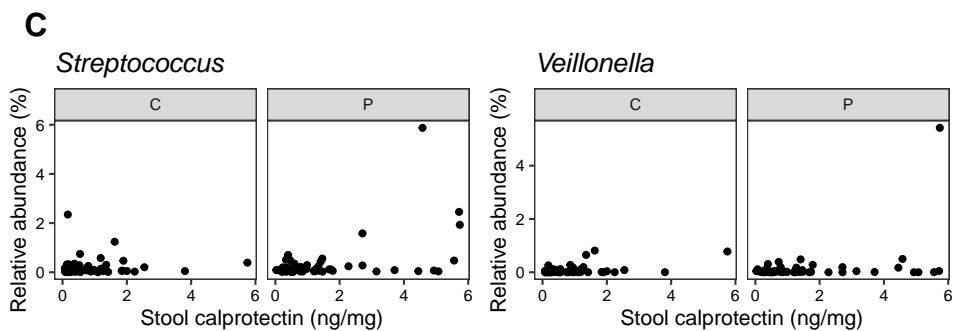

Supplement: Supplementary file 12 — Additional file 12: Scatter plots of genera relative abundances by stool inflammatory and permeability marker levels. Scatterplots visualizing significant (p< 0.05, Pearson correlation) relationships between A) NGAL, B) zonulin, and C) calprotectin and relative abundance of most differentially abundant bacterial genera as determined by differential expression analysis for sequence count data. [file 13024_2021_427_MOESM12_ESM.pdf]
