# Supplementary material for: Relationships of gut microbiota, short-chain fatty acids, inflammation, and the gut barrier in Parkinson’s disease
Source: Mol Neurodegener. 2021 Feb 8;16:6. doi: 10.1186/s13024-021-00427-6 (PMC7869249; doi:10.1186/s13024-021-00427-6)

A

	Alpha diversity: Pearson for Shannon	Alpha diversity: Pearson for inverse Simpson	Beta diversity: adonis
UPDRS IV total	0.002	<0.001	0.001
UPDRS III total	0.002	<0.001	<0.001
UPDRS II total	0.009	0.002	0.001
UPDRS I total	0.039	0.096	0.523
Tremor score poletti	0.048	0.004	0.058
Tremor score jankovic	0.049	0.006	0.123
Tremor by pigd jankovic	0.133	0.034	0.329
Tremor by ar poletti	0.829	0.421	0.901
Time from nms onset	0.161	0.129	0.068
Time from motor onset	0.651	0.587	0.174
Pigd score jankovic	0.032	0.006	0.010
Meds MAO inhibitor	0.160	0.180	0.971
Meds dopamine agonist	0.068	0.063	0.023
Meds dopa	0.474	0.127	0.026
Meds COMT inhibitor	0.756	0.477	<0.001
Hoehn and Yahr	0.023	0.006	0.082
Akinetic rigid score poletti	0.008	0.002	0.005
Age NMS onset	0.920	0.773	0.093
Age motor symptoms onset	0.312	0.396	0.195

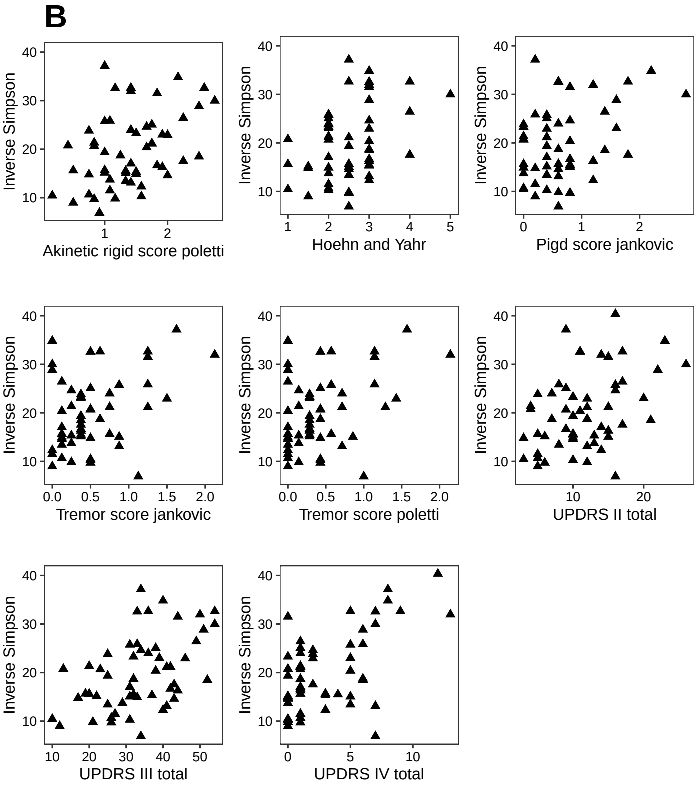

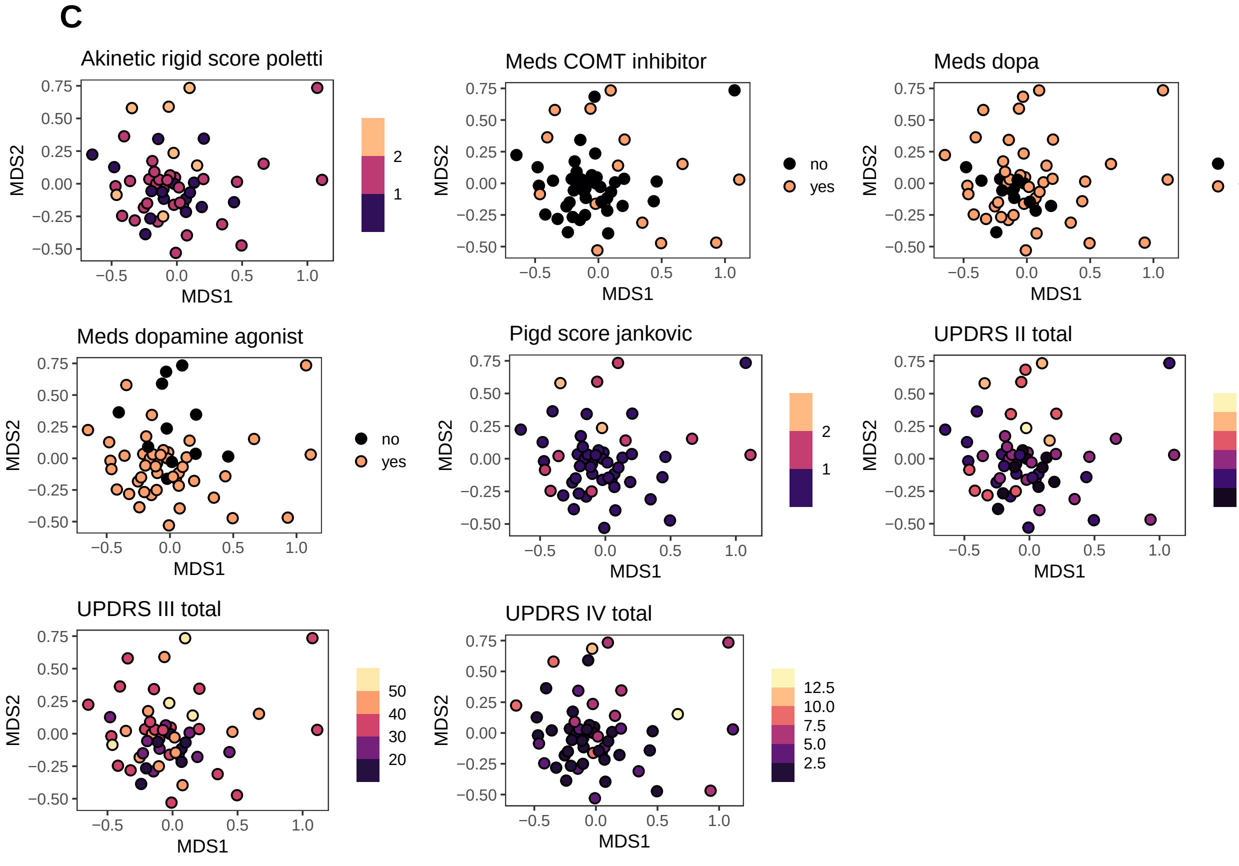

Supplement: Supplementary file 5 — Additional file 5: Microbial diversity and PD-related variables. A) Table summarizing p-values for comparisons of alpha and beta diversity; B) Scatterplots of significant correlations for alpha diversity and PD-related variables; C) NMDS ordination plots for PD-related variables. [file 13024_2021_427_MOESM5_ESM.pdf]
